# Complex regulation in a *Comamonas* platform for diverse aromatic carbon metabolism

**DOI:** 10.1038/s41589-022-01237-7

**Published:** 2023-02-06

**Authors:** Rebecca A. Wilkes, Jacob Waldbauer, Austin Carroll, Manuel Nieto-Domínguez, Darren J. Parker, Lichun Zhang, Adam M. Guss, Ludmilla Aristilde

**Affiliations:** 1 Department of Biological and Environmental Engineering, College of Agriculture and Life Sciences, Cornell University, Ithaca, NY USA; 2 Department of Civil and Environmental Engineering, McCormick School of Engineering and Applied Science, Northwestern University, Evanston, IL USA; 3 Department of the Geophysical Sciences, University of Chicago, Chicago, IL USA; 4 Biosciences Division, Oak Ridge National Laboratory, Oak Ridge, TN USA; 5 The Novo Nordisk Foundation Center for Biosustainability, Technical University of Denmark, Kongens Lyngby, Denmark; 6 Northwestern Center for Synthetic Biology, Evanston, IL USA

**Keywords:** Metabolic pathways, Metabolic engineering, Proteomics, Metabolomics

## Abstract

Critical to a sustainable energy future are microbial platforms that can process aromatic carbons from the largely untapped reservoir of lignin and plastic feedstocks. *Comamonas* species present promising bacterial candidates for such platforms because they can use a range of natural and xenobiotic aromatic compounds and often possess innate genetic constraints that avoid competition with sugars. However, the metabolic reactions of these species are underexplored, and the regulatory mechanisms are unknown. Here we identify multilevel regulation in the conversion of lignin-related natural aromatic compounds, 4-hydroxybenzoate and vanillate, and the plastics-related xenobiotic aromatic compound, terephthalate, in *Comamonas testosteroni* KF-1. Transcription-level regulation controls initial catabolism and cleavage, but metabolite-level thermodynamic regulation governs fluxes in central carbon metabolism. Quantitative ^13^C mapping of tricarboxylic acid cycle and cataplerotic reactions elucidates key carbon routing not evident from enzyme abundance changes. This scheme of transcriptional activation coupled with metabolic fine-tuning challenges outcome predictions during metabolic manipulations.

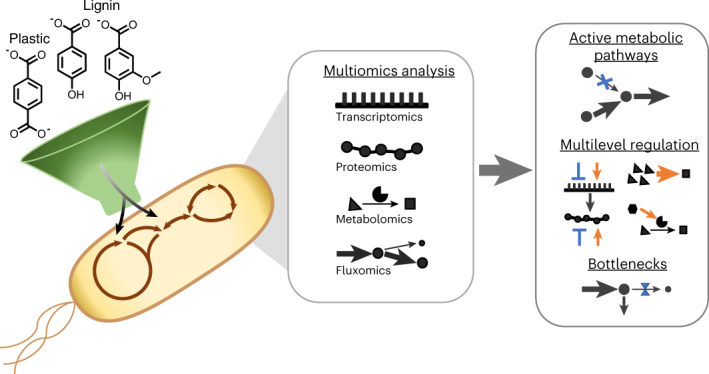

## Abstract (web summary)

Aromatic carbon fluxes for the metabolism of lignin and plastics derivatives in *Comamonas testosteroni* KF-1 are controlled by pathway-specific regulation at transcript, protein or metabolite levels. The combination of transcriptional activation and metabolic fine-tuning complicates predictions of modulated carbon and energy fluxes during metabolic engineering strategies.

## Main

**Fig. 1:**
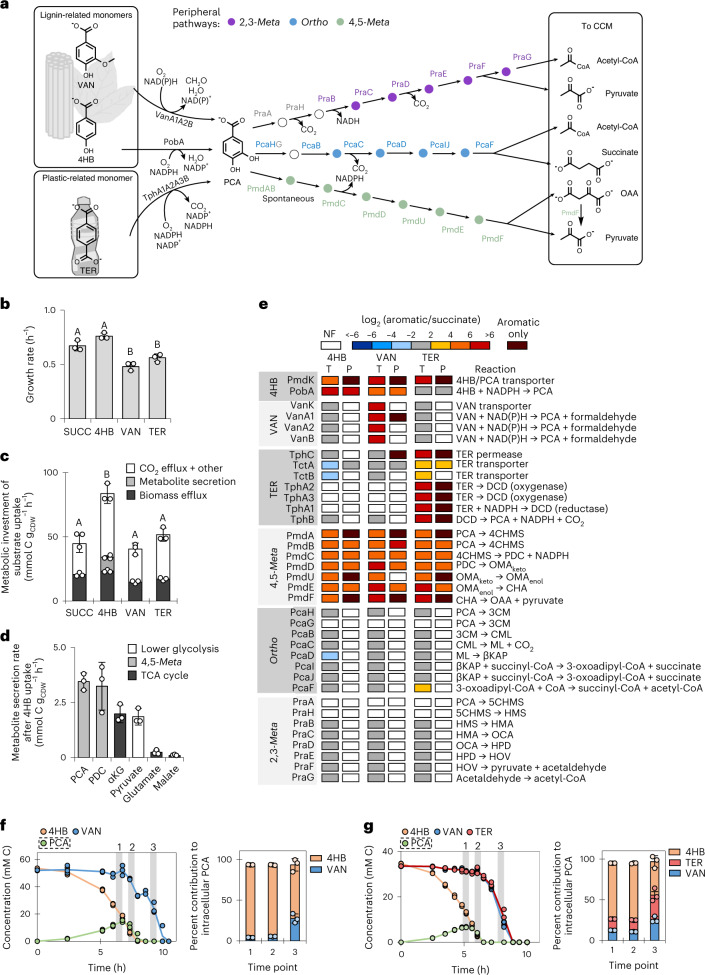
Peripheral metabolic pathways for aromatic compound catabolism. **a**, Schematic of pathways and enzymes characterized for aromatic compounds funneled through PCA and into the 4,5-*meta* (green), *ortho* (blue) or 2,3-*meta* (purple) pathways. Gray enzymes indicate that the gene was not annotated in the *C. testosteroni* KF-1 genome. **b**,**c**, Growth rate (**b**) and partitioning of total carbon uptake rates (**c**) into biomass efflux rate, total metabolite secretion rate and other efflux rates in *C. testosteroni* KF-1 during growth on succinate (SUCC), 4HB, VAN and TER (also see Supplementary Table 1). **d**, Secretion rates of metabolites during growth on 4HB. For **b** and **c**, statistically significant differences (*P* < 0.05) are denoted by a change in letter; significance was determined by one-way ANOVA followed by Tukey HSD post hoc tests. Exact *P* values can be found in Supplementary Data. **e**, Heat map of transcriptomics (T) and proteomics (P) data during growth on 4HB, VAN or TER relative to growth on succinate. The corresponding reactions for each enzyme are shown on the right. Values are expressed as the log_2_ fold change between growth conditions for transcriptomics (*n* = 3) or proteomics (*n* = 4). **f**,**g**, Extracellular substrate depletion profiles (left) and fractional contribution to the intracellular PCA pool (right) for cells grown on 4HB and VAN (**f**) or on 4HB, VAN and TER (**g**). For **b**, **c**, **d, f** and **g**, data are presented as mean values ± s.d. of three biological replicates. For **f** and **g**, assimilation of each substrate into cellular metabolites was determined by using a mixture containing ^13^C-labeled and unlabeled substrates. Details on the data and the names or identifiers are shown in Supplementary Tables 2–5 for the gene transcripts and protein abundances in the initial catabolic pathways and the three putative PCA cleavage pathways. The following are the metabolite abbreviations used in **d** and **e**: DCD, 1,2-dihydroxy-3,5-cyclohexadiene-1,4-dicarboxylic acid; 4CHMS, 4-carboxy-2-hydroxymuconate-6-semialdehyde; OMA_keto_, 4-oxalomesaconate keto form; OMA_enol_, 4-oxalomesaconate enol form; CHA, 4-carboxy-4-hydroxy-2-oxoadipate; 3CM, 3-carboxy-*cis*,*cis*-muconic acid; CML, 4-carboxymuconolactone; ML, muconolactone; βKAP, β-ketoadipate; 5CHMS, 5-carboxy-2-hydroxymuconate-6-semialdehyde; HMS, 2-hydroxymuconate-6-semialdehyde; HMA, 2-hydroxymuconate; OCA, 4-oxalocrotonate; HPD, 2-hydroxypenta-2,4-dienoate; HOV, 4-hydroxy-2-oxovalerate.

*Comamonas* species are promising cellular chassis for the bioprocessing of aromatic-containing waste streams^1–6^. Strains of *C. testosteroni* lack the genes required for carbohydrate utilization and have an innate preference for gluconeogenic substrates such as aromatic compounds^3,7^. In particular, *C. testosteroni* KF-1 was isolated from sewage sludge for its capacity to degrade aromatic synthetic laundry surfactants^3^. Metabolic regulation in *C. testosteroni* strains during assimilation of aromatic compounds remains to be elucidated, thus severely limiting the potential exploitation of these wastewater isolates to metabolize xenobiotic aromatic compounds. In this work, we investigate native mechanisms controlling carbon routing in *C. testosteroni* KF-1 grown on two lignin-associated monomers (4-hydroxybenzoate (4HB) and vanillate (VAN)) and a synthetic polymer-associated monomer (terephthalate (TER)), which are all funneled through protocatechuate (PCA) toward the central carbon metabolism (CCM; Fig. 1a)^3^.

PCA is a key intermediate in the catabolism of hydroxybenzoates, methoxybenzoates and phthalates^8,9^. There are three known catabolic pathways to initiate ring opening of PCA at the *ortho* position, the 2,3-*meta* position or the 4,5-*meta* position (Fig. 1a)^9^. Enzymes in each pathway were characterized in *Pseudomonas putida* KT2440 (*ortho* pathway)^10^, *Paenibacillus* sp. JJ-1B (2,3-*meta* pathway)^11^, *Sphingobium* sp. SYK-6 (4,5-*meta* pathway)^12,13^, and *C. testosteroni* CNB-1 (4,5-*meta* pathway)^5^, among others (Fig. 1a)^5^. Catabolism of aromatic compounds can both generate and consume cofactors before carbon influx into the tricarboxylic acid (TCA) cycle. For instance, 4HB hydroxylation into PCA requires reducing power in the form of NADPH or NADH^5,14^, VAN demethylation through VanAB (a Rieske domain ring-hydroxylating oxygenase) requires NAD(P)H and produces formaldehyde^3,15^, and TER decarboxylation to PCA requires NADPH during oxidation and produces NADPH in a dehydrogenase reaction (Fig. 1a)^16,17^. To generate NADPH in CCM, *C. testosteroni* strains rely on isocitrate dehydrogenase (IDH) in the TCA cycle and malic enzyme (ME) in the cataplerotic direction because these strains lack the genes for a complete oxidative pentose phosphate (PP) pathway^3,18^. Transhydrogenase reactions can also interconvert between NADH and NADPH pools to supply cofactors for catabolic reactions or biosynthetic demand^18^.

The presence of the complete *pmd* operon in the genome of *C. testosteroni* KF-1 implicates the 4,5-*meta* pathway for catabolism^3^, but this has not yet been verified experimentally. The 4,5-*meta* pathway consists of six enzymatic steps to convert PCA into pyruvate and oxaloacetate (OAA) without any carbon loss to CO_2_ (Fig. 1a)^5^. In this pathway, one NADPH is generated during the production of 2-pyrone-4,6-dicarboxylate (PDC; Fig. 1a)^19,20^. Potential genes for the *ortho* and 2,3-*meta* pathways are dispersed throughout the *C. testosteroni* KF-1 genome, but homologs of *pcaG* in the *ortho* pathway and *praA* and *praH* in the 2,3-*meta* pathway were not annotated (Fig. 1a)^3^. Due to possible multifunctional enzymes compensating for absent genome annotations^21^, the functionality of the *ortho* and 2,3-*meta* cleavage pathways remains to be ascertained.

Turning pathways ‘on’ or ‘off’ by regulating gene expression is a recognized strategy in bacteria to promote growth by conserving costs associated with producing unused enzymes^22,23^. Importantly, flux directionality through metabolic reactions was determined to be related to negative Gibbs free energy (∆*G*), which is log proportional to both the concentration ratio of product to reactant and the flux ratio of forward to backward flux^24,25^. Enzyme abundance was reported to be a poor predictor of carbon fluxes in *P. putida*, *Bacillus subtilis* and *Saccharomyces cerevisiae*, and, instead, additional regulation through metabolite abundances, post-translational modifications or allosteric regulation was proposed^26–28^. Metabolite concentrations in *Escherichia coli*, *Clostridium acetobutylicum* and *C. ljungdahlii* were found to drive the thermodynamic potential controlling metabolic flux directionality without altering enzyme abundance^24,29,30^. Leveraging orthogonal methods to elucidate the metabolic controls underlying aromatic carbon fluxes in *C. testosteroni* KF-1, we combined transcriptomics and proteomics measurements with targeted metabolomics data, ^13^C-kinetic profiling, ^13^C-fluxomics analysis, and ^13^C-isotopomer fragmentation analysis during feeding on 4HB, VAN and TER. Additionally, we established a toolkit for genetic manipulation of *C. testosteroni* KF-1 to validate hypotheses about flux through aromatic carbon cleavage pathways. New insights were obtained on the regulatory mechanisms during aromatic carbon utilization that involve complex relationships between transcript expression, protein abundance, metabolite levels, and metabolic fluxes.

## Results

### Transcript and enzyme levels regulate aromatic ring cleavage

**Extended Data Fig. 1:**
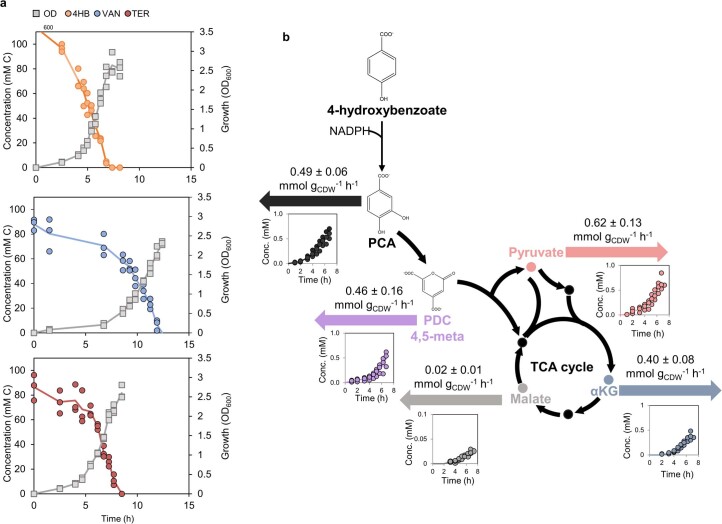
Growth profiles, substrate depletion, and metabolite secretions. **a)** Kinetics of 4HB depletion (orange), VAN depletion (blue), and TER depletion (red) throughout cellular growth (grey) in *C. testosteroni* KF-1. Biological replicates (n = 3) are shown separately with the average represented by the line through the points. **b)** Kinetics and rates of metabolite secretion during growth on 4HB. Secretion data were from biological replicates (n = 3) shown as individual data points. Metabolite abbreviations: protocatechuate, PCA; 2-pyrone-4,6-dicarboxylate, PDC; pyruvate, Pyr; α-ketoglutarate, αKG.

The *C. testosteroni* cells grew well on all three aromatic substrates and succinate (a TCA cycle intermediate used as a reference substrate), with less than 30% difference in the growth rates (Fig. 1b). However, growth on 4HB exhibited a 50% greater carbon equivalent uptake rate than growth on the other compounds (*P* < 0.001; Fig. 1b,c and Extended Data Fig. 1a). The occurrence of metabolite secretions only during growth on 4HB, representing 28% of the 4HB-derived carbon uptake, explained the discrepancy between uptake rate and growth rate (Fig. 1d). This discrepancy implied a surplus of carbon uptake beyond the enzymatic capacity to direct 4HB-derived carbons to biomass. In fact, 60% of the carbon loss from 4HB uptake stemmed from secretions of metabolites (PCA and PDC) from the aromatic catabolism pathways before CCM; the remaining 40% was from pyruvate (a product of the 4,5-*meta* pathway), α-ketoglutarate (αKG) and malate (both TCA cycle metabolites) and glutamate (an amino acid derived from αKG; Fig. 1d and Extended Data Fig. 1b).

**Extended Data Fig. 2:**
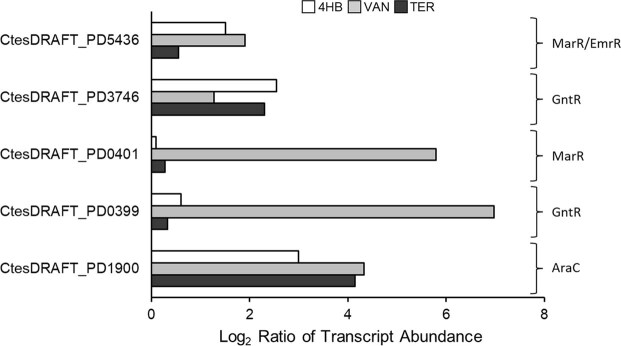
Transcriptional regulators upregulated during growth on aromatic substrates. Transcript abundance represents triplicate measurements of log2 fold changes for cells grown on the aromatic compounds relative to succinate.

**Extended Data Fig. 3:**
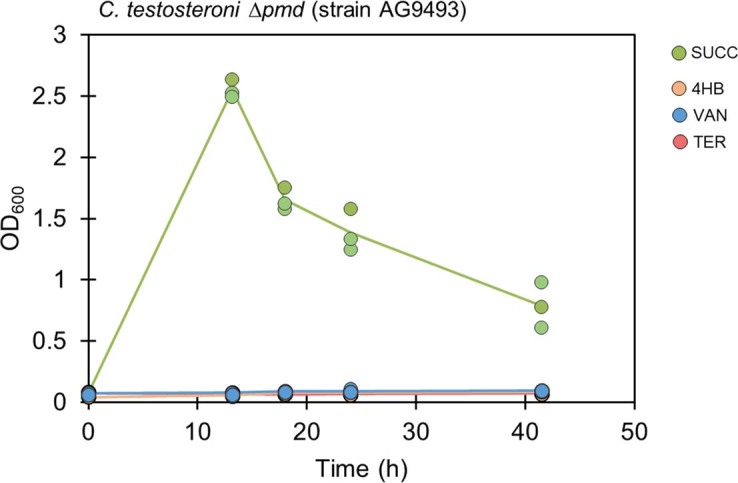
Growth profile of 4,5-*meta* knockout mutant. Strain AG9493 was grown on 100 mM C of succinate (green), 4-hydroxybenzoate (orange), vanillate (blue), or terephthalate (red). No growth was observed on the aromatic compounds after 42 h.

Consistent with the 4,5-*meta* pathway as the primary catabolic route from PCA, growth on the aromatic substrates triggered a greater than 18-fold upregulation of 4,5-*meta* pathway transcripts, transcriptional regulators, and proteins relative to growth on succinate; there was either no differential transcript expression or a lack of detectable proteins for the putative *ortho* and 2,3-*meta* pathways (Fig. 1e, Extended Data Fig. 2 and Supplementary Tables 4–8). As a confirmation, *C. testosteroni* KF-1 mutant cells without the 4,5-*meta* pathway (*C. testosteroni ∆pmd*, called strain AG9493) were incapable of growing on 4HB, VAN or TER (Extended Data Fig. 3). Gene products involved in both the transport and oxidation of VAN or TER were only detected or were differentially expressed (by up to 1,230-fold) during growth of *C. testosteroni* KF-1 on the respective substrate (Fig. 1e). However, gene products of CtesDRAFT_PD1893, a PmdK homolog with 98% identity to PmdK in *Comamonas* sp. E6 and annotated as both a 4HB and PCA transporter^6^, were detected or elevated by 34- to 72-fold during growth on all three aromatic substrates relative to growth on succinate (Fig. 1e and Supplementary Table 9). During single-substrate growth on 4HB or VAN, there was an increase (from 27- to 200-fold) in both the transcript and protein for CtesDRAFT_PD2627 (PobA homolog; responsible for the oxidation of 4HB to PCA; Fig. 1e). In sum, relative to succinate-fed cells, growth on all three aromatic substrates triggered the upregulation of gene products relevant to transport and initial catabolism of each substrate toward the 4,5-*meta* pathway as the assimilation route into CCM (Fig. 1e).

The transcriptomics data further implied the capacity for 4HB uptake in the presence of other aromatic substrates (Fig. 1e). In subsequent growth experiments of *C. testosteroni* KF-1 on a mixture of 4HB and VAN, 4HB contributed about 90% to the intracellular PCA pool until late exponential growth (Fig. 1f). During growth on a mixture of all three aromatic compounds, VAN and TER were only co-utilized at a detectable rate after 4HB was depleted (Fig. 1g, time point 3). However, even with their minimal depletion extracellularly during early exponential growth, VAN and TER each contributed about 13% each to the intracellular PCA pool (Fig. 1g). Our data seemed to indicate possible carbon catabolite repression to support preferential uptake of 4HB before VAN or TER, but the lack of a complete shutdown of the initial catabolic pathways for both VAN and TER pointed instead to a disproportionate rate utilization of the different substrates. Uptake regulation of substrate mixtures would be an important research avenue to address moving forward with *C. testosteroni* as a performance strain. Here, toward building the requisite foundation for future metabolic manipulations of *C. testosteroni*, we focus on gaining fundamental mechanistic insight on aromatic carbon assimilation into CCM.

### Aromatic carbon flux into the TCA cycle is regulated at the OAA node

**Fig. 2:**
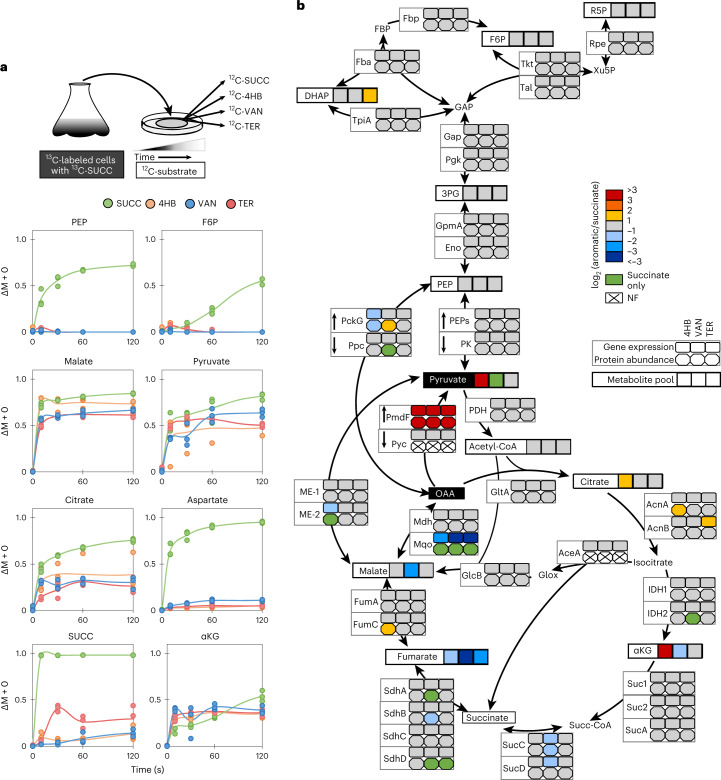
Isotope kinetics, transcriptomics, proteomics, and metabolomics analysis of CCM during assimilation of aromatic compounds. **a**, Experimental kinetic incorporation of the non-labeled fraction (M + 0) over time in seconds after carbon switch to succinate (green), 4HB (orange), VAN (blue) or TER (red). Lines represent the mean of the three independent biological replicates. Extended data up to 1,800 s are shown in Supplementary Fig. 1. Above the data plots is a schematic of the carbon switch experiment using transfer of cells grown on fully ^13^C-labeled succinate in liquid growth medium to plates containing unlabeled succinate, 4HB, VAN or TER; the switch from succinate to succinate was the positive control. **b**, Differential log_2_ fold changes in transcript abundance (rectangles), protein abundance (hexagons) and quantified metabolite pools (squares) during steady-state growth on 4HB, VAN or TER relative to growth on succinate. Transcriptomics and metabolomics data were obtained from three biological replicates; proteomics data were obtained from four biological replicates. The following is the color code for **b**: white with an X indicates that the transcript or protein was not found (NF) in the specified condition, green indicates that the transcript, protein or metabolite was only found during growth on succinate, yellow/red and light/dark blue tones indicate elevated and depleted levels, respectively, in the condition with an aromatic carbon relative to the succinate-only condition, and gray indicates no change. Details on data and the names or identifiers are shown in Supplementary Table 10 for the gene transcripts and in Supplementary Table 11 for the protein abundances in CCM. Both OAA and pyruvate (highlighted in black boxes) were entry points for 4HB-derived carbons from the 4,5-*meta* cleavage pathway. The following are the abbreviations used for metabolites: G6P, glucose-6-phosphate; F6P, fructose-6-phosphate; FBP, fructose-1,6-bisphosphate; DHAP, dihydroxyacetone phosphate; GAP, glyceraldehyde-3-phosphate; Ru5P, ribulose-5-phosphate; Xu5P, xylulose-5-phosphate; R5P, ribose-5-phosphate; S7P, sedoheptulose-7-phosphate; E4P, erythrose-4-phosphate; 3PG, 3-phosphoglycerate; 2PG, 2-phosphoglycerate; Glox, glyoxylate.

After switching cells from ^13^C-succinate to unlabeled aromatic substrates (or unlabeled succinate as a reference), we tracked the kinetic incorporation of the non-labeled fraction (M + 0) over 120 s (Fig. 2a). A rapid increase in non-labeled pyruvate and malate (up to 70–77% within 10 s) indicated minimal delay of aromatic carbon incorporation despite initial feeding on only succinate (Fig. 2a). Slow incorporation (only 2% after 10 s) into succinate confirmed an inactive *ortho* cleavage pathway (Fig. 2a). Aromatic carbon influx into OAA was retained initially in the TCA cycle due to impeded gluconeogenic flux, as revealed by a low fraction of non-labeled aspartate (an amino acid directly synthesized from OAA; only 10%) and phosphoenolpyruvate (PEP; less than 1%) after 120 s (Fig. 2a). Despite the lack of gluconeogenic flux into the Embden-Meyerhof-Parnas (EMP) and PP pathway metabolites immediately after the substrate switch, there were less than twofold changes in the abundance of gene products in these pathways in cells fed solely on the aromatic substrates relative to succinate-fed cells (Fig. 2b). Enzymes in the Entner–Doudoroff (ED) pathway were not detected here during aromatic substrate catabolism, consistent with a previous report of an active ED pathway during glycolytic growth but not during gluconeogenic growth^18^. Except for pyruvate (a product of the 4,5-*meta* pathway), pools of metabolites in the EMP and PP pathways remained relatively unchanged between growth conditions (Fig. 2b). While fluxes in the TCA cycle for aromatic-derived carbons were not substantially altered relative to growth on succinate, there were significant changes in transcripts, proteins and metabolites. During growth on 4HB compared to succinate, there was 2.5-fold higher protein abundance of both AcnA and FumC, whereas there was 2.5-fold lower protein abundance of the cataplerotic enzymes PckG and ME-2, all of which would facilitate carbon retention in the TCA cycle (Fig. 2b). Additionally, 4HB-grown cells had higher metabolite levels than succinate-grown cells, with 17.5-fold higher αKG, 11-fold higher pyruvate and nearly 2.5-fold higher citrate levels (Fig. 2b). By contrast, growth on VAN exhibited depletion in both proteins and metabolites in the TCA cycle relative to growth on succinate, including undetectable proteins (IDH2, ME-2, Ppc, SdhA and SdhD), 3.3-fold lower protein abundance (SdhB, SucC and SucD), a 2.7-fold lower αKG pool, and an undetectable pyruvate pool (Fig. 2b). However, during growth on TER, the general trend was a lack of appreciable differences in transcripts, proteins or metabolites relative to growth on succinate (Fig. 2b). Despite these substrate-specific differences, the similar kinetic isotopic flux profiling of the TCA cycle implied an underlying regulation that maintained consistent fluxes in a robust metabolic network for *C. testosteroni* KF-1 (Fig. 2a).

Across growth on the three different aromatic substrates relative to growth on succinate, there were differential changes in the expression of gene products that control the pool of OAA, which can act as an enzymatic inhibitor (Fig. 2b)^31–33^. Lack of detectable levels of Pyc protein may mean a limited carboxylation reaction flux from pyruvate to OAA (Fig. 2b). Also, a specific OAA decarboxylase was not found annotated in the *C. testosteroni* KF-1 genome, but PmdF (the last step in the 4,5-*meta* pathway) has previously been shown to decarboxylate OAA into pyruvate in vitro^5^. At the node between OAA and malate, Mqo was downregulated 50-fold at the transcript level and was not detected at the protein level during growth on the aromatic substrates relative to succinate; but, there was an increase (albeit less than twofold) in malate dehydrogenase (Mdh) across both gene products (Fig. 2b). This different regulation of Mqo and Mdh in *C. testosteroni* would support reductive flux to modulate the OAA pool based on previous works with *E. coli* and *Corynebacterium glutamicum*^33,34^ that reported Mqo operating in the direction of malate oxidation to OAA and Mdh favoring reduction of OAA to malate (Fig. 2b). Collectively, during feeding on the aromatic substrates, we found a lack of transcriptional or translational regulation for TCA cycle fluxes but gene product regulation of the OAA pool, which would in turn modulate the flux of the aromatic substrate-derived carbons into CCM (Fig. 2a,b). Using 4HB-grown cells versus succinate-grown cells, we further examined the relationships between transcripts, proteins, metabolites, and fluxes.

### Central carbon fluxes are driven by metabolite pools

**Fig. 3:**
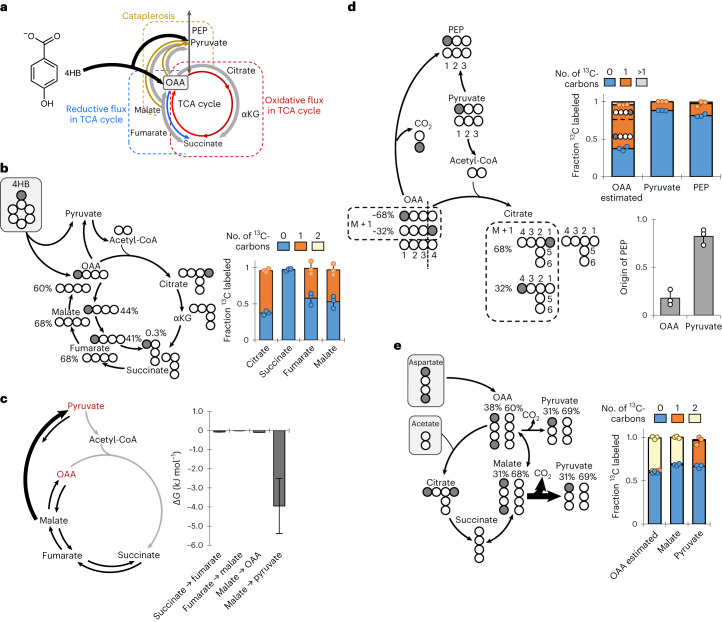
Carbon mapping and free energy elucidate metabolic routing. **a**, Schematic representation of carbon partitioning from the OAA node into irreversible reactions in the TCA cycle (oxidative direction), reversible reactions in the TCA cycle (reductive direction) or cataplerosis. **b**, Reductive flux to fumarate determined by carbon mapping (left) and experimental metabolite labeling data (right) from long-term isotopic enrichment of *C. testosteroni* KF-1 cells grown on [1-^13^C]-(carboxyl)-4HB. **c**, Free energy (∆*G*) calculated from the forward and reverse fluxes determined by ^13^C MFA (also see Supplementary Table 12). Error bars represent standard deviation for the optimized model across six sets of ^13^C-labeling data (*n* = 6). **d**, Relative contribution of OAA versus pyruvate to the PEP pool determined by carbon mapping (left) and experimental data (top) accounting for positional ^13^C labeling of the M + 1 fraction during long-term isotopic enrichment of *C. testosteroni* KF-1 cells grown on [1-^13^C]-(carboxyl)-4HB (see Supplementary Table 13). Decarboxylation of OAA to produce singly labeled PEP would only occur when the ^13^C-carbon is at the first position of OAA. Fractional (mean ± s.d.) contribution of each precursor compound to the pool of PEP was determined from flux ratio analysis of ^13^C labeling (bottom right). **e**, Cataplerotic flux into pyruvate determined by carbon mapping (left) and experimental data (right) for *C. testosteroni* KF-1 cells grown on [1,4-^13^C_2_]-aspartic acid and unlabeled acetate. The percentages shown in the diagram are from experimental data of OAA (estimated from citrate ^13^C-labeling fractions) and malate and the corresponding percentage in pyruvate expected from cataplerosis. For the diagrams in **b**, **d** and **e**, ^13^C-carbons are in gray, and ^12^C-carbons are in white. The following are the metabolite labeling patterns: M + 0 (light blue), M + 1(orange) and M + 2 (light yellow). All data (mean ± s.d.) were from biological replicates (*n* = 3).

**Fig. 4:**
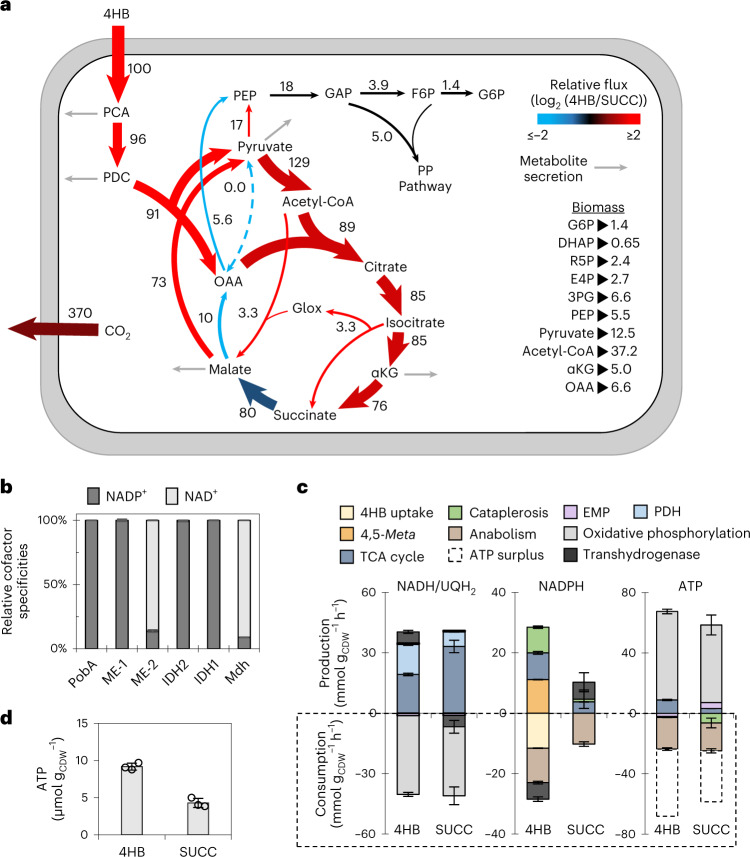
Metabolic flux distributions depict energy and reducing power yields. **a**, Optimized flux model of *C. testosteroni* KF-1 grown on 4HB using compiled ^13^C metabolomics data obtained with three biological replicates across two labeling schemes (*n* = 6; Supplementary Fig. 3 and Supplementary Table 14). All fluxes are normalized to the 4HB uptake rate of *q* = 11.9 ± 1.7 mmol g_CDW_^−1^ h^−1^. Arrows are relative to flux magnitude. Relative flux between cells grown on 4HB and succinate are denoted by a color change. Increased flux is shown in shades of red, decreased flux is shown in shades of blue, and black indicates negligible differences in flux values. **b**, Relative cofactor specificities of purified proteins as a percentage of the sum of specific activity for NAD(P)^+^/NAD(P)H. Error bars represent standard deviation of the mean (*n* = 3). Absolute specific activities of individual replicates are available in Supplementary Table 15. **c**, Modeled absolute production and consumption rates (mmol g_CDW_^−1^ h^−1^) of NADH/FADH_2_, NADPH and ATP determined from the cellular fluxes and species-specific biomass stoichiometry (Supplementary Table 17). Transhydrogenase reactions of NADH to NADPH were assumed to supply the additional NADPH needed for anabolism. The ATP production from NADH/FADH_2_ was calculated using a phosphate-to-oxygen ratio of 1.5. Error bars represent standard deviation of the best-fit solution modeled across six sets of ^13^C-labeling data (*n* = 6). **d**, Quantified intracellular pools of ATP for cells grown on 4HB or succinate. Data are presented as the mean ± s.d. of three biological replicates (*n* = 3). All succinate data or flux calculations used as a reference here were from values previously reported by Wilkes et al.^18^.

Due to the replenishment of the OAA pool by the 4,5-*meta* pathway in aromatic carbon catabolism, contribution of malate carbons to OAA as expected in a canonical TCA cycle was not required (Fig. 3a). Instead, ^13^C mapping revealed that about 40% of fumarate was derived from OAA and 60% was derived from succinate, indicating flux in the reductive direction (Fig. 3b and Supplementary Fig. 2). Near-zero negative values of ∆*G* for the reactions between succinate and OAA demonstrated that these reactions were close to equilibrium (Fig. 3c). For cataplerotic reactions, positional ^13^C mapping showed that pyruvate was the primary source (approximately 82%) of PEP; OAA contributed only 18% (Fig. 3d). Flux to pyruvate was also exclusively from malate and not OAA (Figs. 3e and 4a), which further indicated that PmdF was not involved in cataplerosis. Thus, the main route for cataplerosis was through ME, which was further supported by the nearly tenfold greater carbon flux from malate to pyruvate during 4HB metabolism than during succinate metabolism (Fig. 4a), despite only a modest driving force from malate to pyruvate (∆*G* = −3.95 ± 1.44 kJ mol^–1^) in 4HB-grown cells (Fig. 3c).

In the clockwise canonical TCA cycle, flux from OAA to succinate increased 4-fold, but flux from succinate to malate decreased by 1.6-fold, and flux from malate to OAA decreased by 12.5-fold (Fig. 4a). This substantial decrease in the flux between malate and OAA agreed with the absence of detectable levels of Mqo during growth on 4HB (Figs. 2b and 4a). Consistent with undetected or less than twofold changes in the transcripts and proteins of AceA and GlcB, only a small flux (approximately 3% of 4HB uptake) was through the glyoxylate shunt (Fig. 4a). Likewise, there were less than 50% changes in the relative flux in the EMP and PP pathways between 4HB-grown cells and succinate-grown cells, in agreement with the lack of changes in the amounts of transcripts, enzymes and metabolites in these pathways (Figs. 2b and 4a). However, the observed differences in the TCA cycle and cataplerotic reactions implied substrate-specific mechanisms for converting carbon flux into energy generation (Figs. 2b and 4a,b).

Indeed, we determined different pathways involved in maintaining cofactor balance in response to feeding on 4HB versus succinate (Fig. 4b,c). Comparison of the protein sequences to enzymes with known cofactor specificity^14,35^ suggested that NADPH was the likely cofactor for PobA and PmdC in *C. testosteroni* KF-1 (Supplementary Tables 8 and 16). Subsequent enzymatic assays of purified proteins from *C. testosteroni* KF-1 demonstrated that NADPH was the cofactor for PobA and for ME-1, IDH1 and IDH2, but NADH was the preferred cofactor for ME-2 and Mdh (Fig. 4b). Thus, a net-zero balance in NADPH was calculated from the uptake and channeling of 4HB-derived carbons through the 4,5-*meta* pathway, assuming that PmdC is exclusively NADPH dependent, as confirmed experimentally for PobA (Fig. 4c). The high carbon flux through IDH and ME-1 (ME-2 was not detected during growth on 4HB) downstream of the 4,5-*meta* pathway yielded NADPH in excess of the NADPH requirement for anabolism during growth on 4HB (Fig. 4a–c). Therefore, in contrast to succinate-grown cells that required transhydrogenase-dependent electron transfer to produce NADPH from the NADH pool, 4HB-grown cells instead may use electron transfer from excess NADPH to generate NADH in support of ATP production via oxidative phosphorylation (Fig. 4b), albeit the only transhydrogenase (PntAB) annotated in the *C. testosteroni* KF-1 genome was not differentially expressed (Supplementary Table 10). Despite a similar ATP requirement for anabolism, the flux analysis for 4HB-grown cells determined a 20% greater ATP surplus potentially available for cellular maintenance, which was corroborated by a 50% greater pool of measured intracellular ATP, presumably due to the relatively higher rate of oxidative phosphorylation and substrate-level phosphorylation than observed during growth on succinate (Fig. 4c,d).

**Fig. 5:**
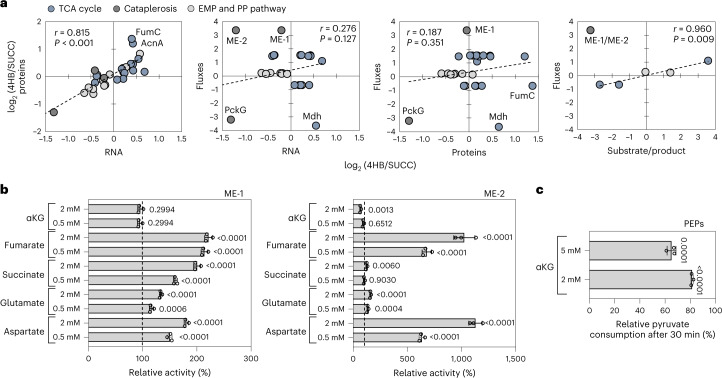
Correlation of omics levels indicates multilevel regulation. **a**, Correlation matrices of relative changes across transcript expression, protein abundances, fluxes, and metabolites (calculated from the substrate-to-product ratio of the quantified metabolite pools; see Supplementary Tables 18 and  19). Pearson correlation coefficients (*r*) and statistical significance of correlation (*P*) determined from two-sided *t*-tests are shown for each correlation matrix. For the relationship between fluxes and metabolites, the outlier ME-1 was removed from the correlation. The transcriptomics data were from three biological replicates (*n* = 3), and proteomics data were from four biological replicates (*n* = 4); the metabolite-level data were obtained with data from three biological replicates (*n* = 3); the fluxomics data were obtained from three biological replicates across two labeling schemes (*n* = 6). **b**, Allosteric regulation of ME-1 (left) and ME-2 (right) in the presence of TCA cycle intermediates and TCA cycle-derived amino acids. Data are presented as relative enzyme activity (as a percentage) in the presence of the effectors compared to the control without the effectors. Reaction profiles are depicted in Supplementary Fig. 4. Absolute specific activities of individual replicates are available in Supplementary Fig. 5 and Supplementary Tables 20 and 21. **c**, Regulation of PEPs in the presence of αKG. Data are presented as the relative pyruvate consumption (as a percentage) for the enzyme with 2 mM or 5 mM αKG compared to the control without αKG present. Pyruvate consumption data for individual replicates are available in Supplementary Fig. 5. For **b** and **c**, all data (mean ± s.d.) were from replicates (*n* = 3). Significance was determined by one-way ANOVA followed by Tukey HSD post hoc tests.

Metabolic flux regulation was further examined through correlation relationships between transcriptomics, proteomics, metabolomics, and fluxomics (Fig. 5a). There was a strong positive linear relationship between the relative levels of RNA and protein (Pearson correlation, *r* = 0.815, *n* = 31, *P* < 0.001; Fig. 5a). The two outliers from this relationship were FumC and AcnA, which were 2.5-fold higher at the protein level during 4HB feeding than during succinate feeding despite the lack of differential expression of the genes, implying potential additional regulation of translation or protein turnover (Figs. 2b and 5a). Notably, there was no correlation between the relative metabolic fluxes and either relative transcript levels (Pearson correlation, *r* = 0.276, *n* = 29, *P* = 0.147) or relative protein abundance (Pearson correlation, *r* = 0.187, *n* = 27, *P* = 0.351), primarily due to nodes in the TCA cycle and cataplerosis (Fig. 5a). Excluding the reaction through ME, there was a strong agreement (*r* = 0.960, *n* = 5, *P* = 0.009) between the central carbon fluxes and the substrate-to-product ratio of metabolite pools (Fig. 5a). These correlation analyses thus revealed that metabolite pools were strongly correlated to TCA cycle fluxes, which ultimately controlled the overall cellular redox balance and energy metabolism in *C. testosteroni*.

### Allosteric regulation influences key metabolic reactions

High flux from malate to pyruvate during growth on 4HB could not be explained at the transcript, protein or metabolite levels. In *E. coli*, favorable operation of ME in the forward decarboxylation direction was found to be regulated positively by succinate, fumarate, aspartate, and glutamate^36^. Here, both ME-1 and ME-2 from *C. testosteroni* KF-1 exhibited strong activation (up to 200% and 1,100% increases in activity, respectively) in the presence of fumarate and aspartate and moderate activation in the presence of succinate and glutamate (up to a 160% increase in activity); only high concentrations of αKG had a significant inhibitory impact on ME-2 (Fig. 5b). Flux through PEP synthetase (PEPs) appeared to be stalled during growth on the aromatic substrates despite the relatively high pool of pyruvate and ATP compared to growth on succinate (Figs. 2a,b and 4d). In the presence of αKG, the utilization of pyruvate by PEPs was decreased by up to 35% (Fig. 5c). Thus, these findings implied that the high flux through ME could be facilitated by fumarate and aspartate levels (and, moderately, by succinate and glutamate), whereas the flux through PEPs could be impeded by the αKG pool.

### Resolution of identified bottlenecks during 4HB catabolism

**Fig. 6:**
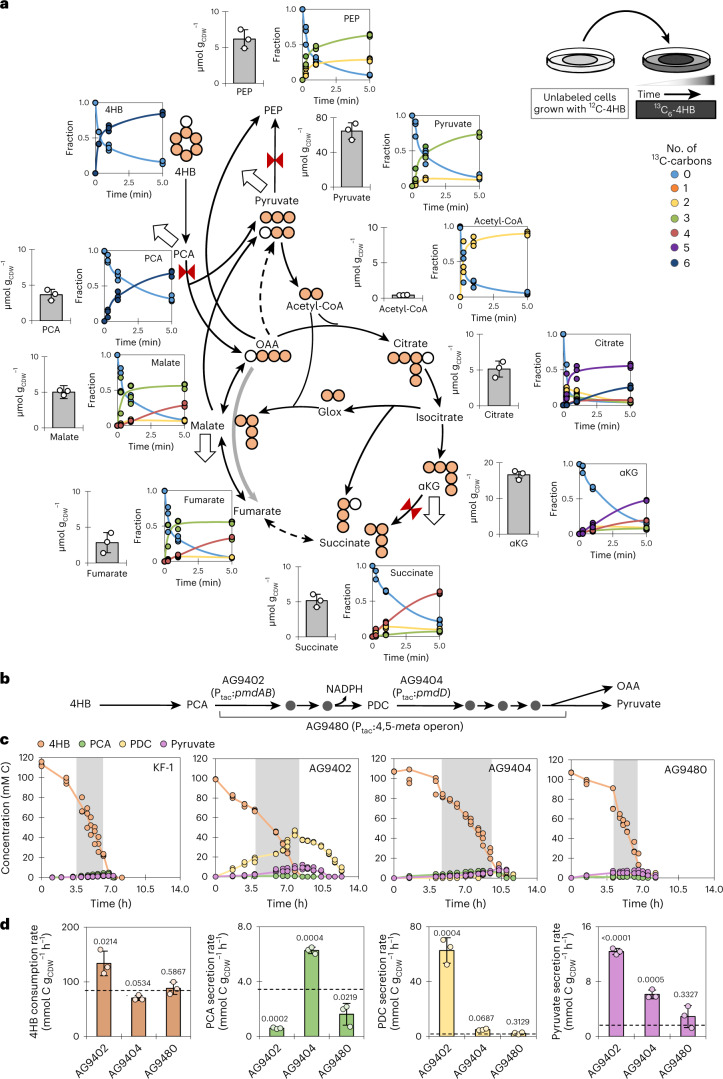
Kinetics of 4HB assimilation highlight bottlenecks in metabolism and targets for metabolic engineering. **a**, Diagram of carbon mapping of ^13^C-carbons (orange) and ^12^C-carbons (white) during the cellular incorporation of [^13^C_6_]-(phenyl)-4HB over time (*t* = 5 min). Bar graphs of quantified metabolite pools (µmol g_CDW_^−1^) are shown next to the scatter plots of the ^13^C kinetics data following 4HB incorporation. Metabolite pools (average ± s.d.) were from biological replicates (*n* = 3). Lines in the kinetic labeling data represent the average of the biological replicates (*n* = 3). Extended time points (up to 30 min) are in Supplementary Fig. 8. Flux direction in the reductive side of the TCA cycle is indicated by the light gray arrow. Potential bottlenecks in metabolic fluxes are represented by red bow ties. Metabolite secretions are denoted by the large white arrows. Low flux exchange is indicated by the dashed arrow. **b**, Schematic of the 4,5-*meta* pathway and the overexpression strains designed to overcome identified bottlenecks. **c**, Time-resolved extracellular profile of 4HB depletion (orange), PCA production (green), PDC production (yellow) and pyruvate production (purple) throughout growth until stationary phase for AG9402 (overexpression of *pmdAB*), AG9404 (overexpression of *pmdD*) and AG9480 (overexpression of the 4,5-*meta* operon) cells grown on 4HB. The time range for the exponential growth phase is highlighted by the gray box. Biological replicates (*n* = 3) are shown separately, with the average represented by the line through the points. **d**, Left to right, consumption rate of 4HB and secretion rates of PCA, PDC and pyruvate for the three overexpression strains grown on 4HB. The dashed line indicates the rate calculated for *C. testosteroni* KF-1. Data are presented as mean ± s.d. of three biological replicates (*n* = 3). Statistically significant differences from the wild-type strain of *C. testosteroni* KF-1 were determined by two-tailed unpaired *t*-tests.

Due to the 50% greater carbon uptake rate during growth on 4HB than during growth on VAN or TER (Fig. 1c), we investigated the associated in vivo cellular kinetic response using ^13^C-labeled 4HB (Fig. 6a). A slowed ^13^C incorporation into PCA (25% lower at 15 s than 4HB labeling kinetics) was consistent with PCA buildup, which led to PCA secretion at 0.49 ± 0.06 mmol per cell dry weight in grams per hour (mmol g_CDW_^−1^ h^−1^); PDC was secreted at a similar rate (0.46 ± 0.16 mmol g_CDW_^−1^ h^−1^; Fig. 1d). These data thus reflected backlogs at both PmdAB and PmdD during initial catabolism of 4HB (Fig. 1a). Transcripts and proteins in the 4,5-*meta* pathway were 1.2- to 4-fold lower during growth on 4HB than during growth on VAN or TER (Fig. 1e and Supplementary Fig. 6), suggesting that catabolism of 4HB-derived carbons was limited by gene expression.

To resolve these bottlenecks, we engineered three strains that overexpressed PmdAB (strain AG9402), PmdD (strain AG9404), or the entire 4,5-*meta* operon (strain AG9480; Fig. 6b). During growth on 4HB, AG9480 grew comparably to the wild type, whereas both AG9402 and AG9404 exhibited slower growth by about 30% (Fig. 6c and Supplementary Fig. 7). Regarding the bottleneck at PCA, there was 83% and 53% lower secretion of PCA with AG9402 and AG9480, respectively, indicating that increased expression of PmdAB facilitated higher flux through this node (Fig. 6d). Interestingly, the resolution of the bottleneck at PmdAB for strain AG9402 was accompanied by both a 60% faster consumption of 4HB and a near 20-fold increase in PDC secretion rate relative to the wild type, highlighting that targeting PmdAB overexpression increased 4HB uptake flux through the 4,5-*meta* pathway but amplified the bottleneck at PmdD (Fig. 6d). Targeting the bottleneck at PmdD by overexpression did not appreciably alter PDC secretion (Fig. 6c,d). However, overexpression of the entire operon alleviated the bottleneck at PCA while maintaining a comparable PDC secretion to the wild type (Fig. 6d). Therefore, the bottleneck at PmdAB can be overcome at the transcriptional level, whereas additional regulation likely modulates flux through PmdD.

Despite a pyruvate secretion rate of 0.62 ± 0.13 mmol g_CDW_^−1^ h^−1^, the pyruvate pool in 4HB-grown cells was the highest of the quantified intracellular metabolites (close to fourfold greater than αKG, the second highest metabolite pool; Fig. 6a), suggesting limited metabolic usage of pyruvate (Fig. 6a). This bottleneck at pyruvate was further exacerbated in AG9402, AG9404 and AG9480, which had over 500%, 200% and 50% increases in pyruvate secretion, respectively (Fig. 6d). These data thus revealed CCM metabolite buildup as a consequence of addressing the bottlenecks in the the 4,5-*meta* pathway. Despite similar ^13^C-labeling kinetics for pyruvate and PEP (reaching a plateau at about 75%), an 11-fold higher pyruvate pool than PEP pool further indicated a bottleneck in the production of PEP from pyruvate (Fig. 6a), as previously discussed (Fig. 2a). A twofold higher fraction of doubly ^13^C-labeled PEP than doubly ^13^C-labeled pyruvate confirmed the contribution of OAA decarboxylation to the PEP pool, as illustrated in Figs. 3d and 4a. The pyruvate pool was 148-fold greater than acetyl-coenzyme A (acetyl-CoA), which would support a strong thermodynamically favorable drive from pyruvate to acetyl-CoA, as demonstrated by the rapid ^13^C labeling of acetyl-CoA in the 4HB-fed cells (Fig. 5). However, comparing 4HB-grown cells to succinate-grown cells, a 12-fold greater pyruvate-to-acetyl-CoA ratio coupled with only a minimal (13%) increase in the PDH enzyme implied that pyruvate conversion to acetyl-CoA was potentially limited by enzyme abundance or the pool of free CoA (Fig. 2b and Supplementary Table 19).

The metabolite OAA represents an important partitioning node between aromatic carbon-derived influx from the 4,5-*meta* pathway, flux to the oxidative side of the TCA cycle, reductive flux to malate and cataplerotic reactions from the TCA cycle (Fig. 3a). Consistent with faster OAA reduction to malate than OAA decarboxylation to pyruvate, there was a higher rate of triply ^13^C-labeled malate (30% after 15 s) than doubly ^13^C-labeled pyruvate (less than 1% after 15 s; Fig. 6a). Furthermore, ^13^C metabolic flux analysis (MFA) indicated minimal flux from OAA to PEP (5.6%; Fig. 4a). Therefore, the OAA pool was primarily partitioned between the oxidative TCA cycle and the reductive flux to succinate. Despite comparable citrate and malate pools in 4HB-grown cells, ^13^C incorporation of OAA-derived carbons was nearly 30% greater into malate (following the M + 3 fraction) than into citrate (following the M + 5 fraction) after 15 s, indicating a higher initial flux to malate than to the oxidative TCA cycle (Fig. 6a). Consistent with this impaired carbon flux in the oxidative direction from citrate to succinate, 50% of citrate was fully ^13^C labeled, but the fully ^13^C-labeled fraction of αKG and succinate was less than 20% after 1 min (Fig. 6a). Moreover, the αKG pool was 3.2-fold greater than the succinate pool during growth on 4HB, leading to secretion of αKG (Figs. 1d and 6a). Collectively, kinetic isotopic flux profiling coupled with metabolite quantitation during aromatic carbon metabolism provided evidence of an incomplete bifurcation of the TCA cycle that culminates at succinate.

## Discussion

Biological upcycling or remediation of recalcitrant aromatic compounds from waste streams warrants systematic investigations of metabolism across model and non-model organisms^20,37–41^. Here, we showed that the wastewater isolate *C. testosteroni* KF-1 grew well on three aromatic compounds (4HB, VAN and TER) related to lignin and plastic derivatives, funneled substrate carbons through PCA into the 4,5-*meta* pathway, produced NADPH in excess of anabolic demand, and generated an ATP surplus. High 4HB uptake was accompanied by secretions of bioproduction value, including PDC, αKG, glutamate, malate, and pyruvate^20,42–44^. These inherent characteristics highlight the potential of *C. testosteroni* as an emerging cellular chassis, but it has remained a relatively untapped organism compared to model species such as *E. coli* or *P. putida*^1^. As a requisite to exploiting *C. testosteroni* and other non-model species, comprehensive multiomics studies of their cellular physiology are necessary^37^.

Discrepancies between changes in gene products and changes in metabolic fluxes were reported in *S. cerevisiae*, *Pseudomonas* species, and *Bacillus* species^26–28,45,46^. Here, relative flux changes in the TCA cycle could not be explained by changes in either transcript or protein abundance in *C. testosteroni*, in contrast to transcriptional regulation of TCA cycle flux reported for *E. coli*^47^ or *B. subtilis* fed on non-aromatic substrates^28^. The only exception was the downregulation of Mqo versus upregulation of Mdh in aromatic substrate-fed versus succinate-fed *C. testosteroni* to favor reductive flux from OAA, in accordance with previously reported differential roles of Mdh versus Mqo in *E. coli*^33^. However, the remaining fluxes in the TCA cycle were correlated positively with the substrate-to-product ratio of metabolite pools, highlighting that directionality of reversible reactions relied on mass action principles, as previously demonstrated in *S. cerevisiae*^27^. Lastly, we concluded that the constrained flux through PEPs could be facilitated by our confirmed allosteric inhibition by αKG^31,32^.

In contrast to the TCA cycle fluxes, cataplerotic fluxes that partition carbon between the TCA cycle and gluconeogenesis were not controlled by the substrate-to-product ratios. Our quantitative ^13^C flux analysis and ^13^C mapping determined low flux from OAA to PEP, no flux between OAA and pyruvate, and high flux from malate to pyruvate. The downregulation of PckG during growth on 4HB indicated that transcript-level regulation moderated flux from OAA to PEP. Despite reported in vitro decarboxylation of OAA to pyruvate by PmdF from *C. testosteroni* CNB-1 (ref. ^5^), the in vivo absence of this activity in *C. testosteroni* KF-1 suggested either a lack of decarboxylase activity for CtesDRAFT_PD1895 or a different metabolic control. Comparing aromatic substrate-fed cells to succinate-fed cells, the relative ratio of the malate to pyruvate pools was incongruent with the high flux through ME. Further, there was a discrepancy between flux changes and changes in gene product abundance for ME. In agreement with previous work with *E. coli*^36^, we found that fumarate, succinate, aspartate, and glutamate increased the activity of ME-2 and ME-1 from *C. testosteroni*. Consistent with our metabolome data and metabolic flux modeling, we concluded that the flux through ME in *C. testosteroni* may be facilitated by the reductive flux from OAA to fumarate.

Rational strain engineering requires a fundamental understanding of the regulatory mechanisms responsible for substrate catabolism, carbon fluxes in CCM, and energy metabolism^37^. A multiomics analysis of several *E. coli* strains identified those best suited for producing amino acids and non-native compounds based on strain-specific regulation of metabolic pathways^48^. Similarly, in *B. subtilis*, identification of altered fluxes due to genome reduction or glutamate presence revealed that high PP pathway flux promoted the production of cellulase^49^. Relative to the other aromatic catabolic pathways, expression of an exogenous 4,5-*meta* pathway in *P. putida* resulted in the highest production of pyruvate^50^. Here, overexpression of genes in the 4,5-*meta* pathway of *C. testosteroni* alleviated the intracellular buildup and secretion of PCA by mitigating the bottleneck at PmdAB. However, the bottleneck at PmdD was not relieved by overexpression, suggesting that post-translational modification or allosteric regulation may influence this enzyme. Alternatively, gene knockouts could be paired with targeted overexpression of PmdAB to promote secretion of value-added products such as PDC, but metabolic manipulation may result in the imbalance of cellular cofactor production. Thus, rational engineering will need to consider how to sustain the cofactor supply, for instance by maintaining or increasing fluxes through IDH and ME, both of which were identified here as critical to sustaining cofactor production.

In sum, we deciphered the following complex regulatory mechanisms in *C. testosteroni* KF-1 grown on aromatic compounds relevant to lignin and plastic feedstocks: transcriptional regulation for aromatic carbon catabolism before CCM, metabolite-level regulation through primarily mass action principles or coupled with protein abundances for the TCA cycle fluxes, and protein-level or allosteric regulation for the cataplerotic reactions partitioning TCA cycle fluxes to gluconeogenesis. These new multiomics perspectives combined with targeted ^13^C mapping of specific metabolic nodes present a framework of guiding principles to exploit metabolic reactions for aromatic carbon catabolism in *C. testosteroni* and other bacterial specialists of biotechnological relevance.

## Methods

### Culturing conditions

Cells of *C. testosteroni* KF-1 (DSMZ 14576) were obtained from Deutsche Sammlung für Mikroorganismen und Zellkulturen. Stocks of cells were made in nutrient-rich lysogeny broth (LB) and 25% glycerol for storage at −80 °C. Growth experiments of *C. testosteroni* KF-1 were performed in an incubator (model I24, New Brunswick Scientific) set at 30 °C with shaking at 220 r.p.m. All growth experiments were conducted in three replicates from the same stock culture. The minimal-nutrient medium contained the major nutrient salts (5.0 mM NaH_2_PO_4_, 20 mM K_2_HPO_4_, 37 mM NH_4_Cl, 17 mM NaCl, 0.81 mM MgSO_4_·7H_2_O and 34 μM CaCl_2_·2H_2_O) and essential trace metal nutrients (13 μmol liter^–1^ CuSO_4_·5H_2_O, 0.49 μmol liter^–1^ H_3_BO_3_, 35 μmol liter^–1^ ZnSO_4_·5H_2_O, 2.9 μmol liter^–1^ MnSO_4_·5H_2_O, 0.11 μmol liter^–1^ NiCl_2_·5H_2_O, 0.6 μmol liter^–1^ Na_2_MoO_4_·5H_2_O and 30 μmol liter^–1^ FeSO_4_·7H_2_O), as previously reported^18^. The carbon source in the filter-sterilized (Waters Corporation; 0.22-μm nylon filters) and pH-adjusted (7.0) minimal-nutrient medium was 100 mM C 4HB (14.3 mM), TER (12.5 mM), VAN (12.5 mM) or succinate (25 mM). For growth on mixtures, the total substrate concentration was kept at 100 mM C in the 1:1 4HB:VAN mixture and 1:1:1 in the 4HB:VAN:TER mixture. Chemicals for standards and growth medium were purchased from Millipore-Sigma, Fisher Scientific or Cayman Chemical. All experiments were conducted with two transfers into experimental growth conditions to acclimate cells. The final transfer for experimental growth was into 125-ml or 250-ml baffled flasks at one-fifth volume to promote oxygen exchange.

### Physiological characterizations

The optical density at 600 nm (OD_600_) on an Agilent Cary UV-visible spectrophotometer was used to monitor cell growth until late stationary phase. Cell suspensions were diluted when the OD_600_ reading was above 0.4 to maintain accurate measurements. To determine substrate consumption rates, metabolite secretion rates and g_CDW_, cell suspension samples of three biological replicates were collected periodically throughout growth and pelleted with 5 min of centrifugation at 9,800 *g* and 4 °C. The supernatant was removed, filtered (Costar Spin-X, 0.22-μm-pore-size filter) and stored at −20 °C until liquid chromatography–mass spectrometry (LC–MS) or ultra-high-performance liquid chromatography (UHPLC) analysis to determine substrate depletion and metabolite secretion profiles. The pelleted cells were lyophilized for g_CDW_ analysis as previously described^18^. Substrate consumption rates (mmol g_CDW_^−1^ h^−1^) and secretion rates (mmol g_CDW_^−1^ h^−1^) were determined from the rates of disappearance and appearance of metabolites, respectively, in the medium using linear fit regression analysis and accounting for growth rate and the g_CDW_. Metabolites released with increasing trends during exponential growth were considered secretions. We did not attempt to distinguish between active and passive release of metabolites but assumed that cell damage or lysis contributed minimally to these secretions, as determined previously^51^. Conversion factors between OD_600_ and g_CDW_ were calculated for *C. testosteroni* KF-1 grown on 4HB (0.26 g_CDW_ liter^−1^ OD_600_^−1^), VAN (0.42 g_CDW_ liter^−1^ OD_600_^−1^), TER (0.47 g_CDW_ liter^−1^ OD_600_^−1^), the 1:1 mixture of 4HB and VAN (0.25 g_CDW_ liter^−1^ OD_600_^−1^) and the 1:1:1 mixture of all three aromatic compounds (0.30 g_CDW_ liter^−1^ OD_600_^−1^). For the overexpression strains grown on 4HB, the conversion factors were 0.29 g_CDW_ liter^−1^ OD_600_^−1^, 0.26 g_CDW_ liter^−1^ OD_600_^−1^ and 0.25 g_CDW_ liter^−1^ OD_600_^−1^ for AG9402, AG9404 and AG9480, respectively.

### Transcriptomics analysis

Cells were grown in three replicates on minimal-nutrient medium with 100 mM C succinate, 4HB, VAN or TER as sole carbon sources. At an OD_600_ of about 1.0 (mid-exponential growth), an aliquot of cell culture (500 μl) was added to a double volume (1 ml) of RNAprotect bacteria reagent (Qiagen). Samples were pelleted by centrifugation at 5,000 *g* for 10 min and stored overnight at −80 °C before total RNA extraction. Enzymatic lysis of the bacteria cells was conducted using lysozyme prepared in molecular-grade TE buffer, and extractions were conducted using an RNeasy mini kit (Qiagen). Samples were sent to the NUSeq Core Facility (Northwestern University) for TruSeq total RNA-sequencing library prep and Illumina HiSeq sequencing (50-base pair (bp), single-end reads). Reads were mapped to the *C. testosteroni* KF-1 genome (ASM16885v1) obtained from NCBI^3^ using Kallisto version 0.46.0 (ref. ^52^). Transcript abundance and differential gene expression were calculated with Voom and Limma using Degust^53^.

### Proteomic analysis

Cells of *C. testosteroni* KF-1 (15 ml) were collected in quadruplicate at an OD_600_ of approximately 1.0 during growth on minimal-nutrient medium with 100 mM C 4HB, VAN or TER as sole carbon sources. Cell pellets were resuspended in a reducing and denaturing buffer (SDS (1%)/Tris (200 mM, pH 8.0)/DTT (10 mM)) and extracted by vortexing and heating (95 °C, 20 min), and cysteine thiols were alkylated with 40 mM iodoacetamide. A modified enhanced filter-aided sample preparation protocol^54^ with a Sartorius Vivacon 500 concentrator (30-kDa nominal cutoff) were used to purify proteins before overnight digestion with MS-grade trypsin at 37 °C. Peptides eluted from the concentrator were dried by vacuum centrifugation and isotopically labeled at both N and C termini using the diDO-IPTL methodology for quantitative analysis, as described previously^55^. In brief, N termini were labeled with either undeuterated or dideuterated formaldehyde, whereas C termini were labeled with either ^16^O or ^18^O (ref. ^55^). An internal standard for quantification was made from pooled peptide aliquots of each sample labeled with formaldehyde/^18^O. For LC–MS analysis, dideuterated formaldehyde/^16^O-labeled sample peptides and formaldehyde/^18^O-labeled internal standard were mixed 1:1 (vol/vol). Quantification of peptides was conducted on a Dionex UltiMate 3000 LC system with nanoelectrospray ionization (Proxeon Nanospray Flex source) coupled to an Orbitrap Elite mass spectrometer (Thermo Fisher Scientific) operating in a data-dependent acquisition mode, as described previously^55^. In brief, chromatographic separation occurred on a monolithic capillary C18 column (GL Sciences Monocap Ultra, 100-µm inner diameter × 200-cm length) using a water–acetonitrile and 0.1% formic acid gradient, and mass spectra were collected with high-resolution (120,000 *m*/∆*m*) MS1 parent ion full scan with fragment ion scans of selected precursors. Analysis of proteomic mass spectra was conducted using MorpheusFromAnotherPlace^55^. For MorpheusFromAnotherPlace analysis, the mass tolerance of precursor and product ions was set at 20 ppm and 0.6 Da, respectively, and modifications included static cysteine carbamidomethylation and variable methionine oxidation, N-terminal (d4)-dimethylation and C-terminal ^18^O_2_. Target–decoy searching was used to control the false discovery rate for peptide spectrum matches to <0.5%. Protein-level relative abundances and standard errors were calculated in R using the Arm postprocessing scripts for diDO-IPTL data^55^ (github.com/waldbauerlab).

### Intracellular metabolite extraction and isotope experiments

Intracellular metabolites were extracted from three biological replicates during exponential growth on unlabeled or ^13^C-labeled growth medium by filtering and lysing cells as described previously^18^. In brief, cells were collected via filtration and rapidly quenched with 2:2:1 cold (−20 °C) methanol:acetonitrile:water. After centrifugation, the liquid suspensions of metabolites were dried under N_2_ gas and stored at −20 °C until LC–MS analysis. Metabolites from unlabeled intracellular extracts were quantified using commercial standards on the software Thermo Scientific Xcalibur 3.0 Quan Browser with an *R*^2^ coefficient of 0.990 or higher for the calibration curve. For long-term isotopic enrichment experiments, cells were washed with minimal-nutrient medium between transfers into ^13^C-labeled medium to remove unlabeled extracellular compounds. The ^13^C-labeled substrates used in this study were [U-^13^C_4_]-succinate, [1-^13^C]-(carboxyl)-4HB, [^13^C_6_]-(phenyl)-VAN and [1,4-^13−^C_2_]-aspartic acid acquired from Cambridge Isotopes and [^13^C_6_]-(phenyl)-4HB obtained from Millipore-Sigma. To determine substrate incorporation into PCA, cells were grown on [1-^13^C]-(carboxyl)-4HB and unlabeled VAN for the mixture of two aromatic compounds and [1-^13^C]-(carboxyl)-4HB, [^13^C_6_]-(phenyl)-VAN and unlabeled TER for the mixture of all three aromatic compounds. After intracellular metabolites were identified and examined by LC–MS, isotopolog fractions were analyzed using Metabolomic Analysis and Visualization Engine software version 2011.6.17 (ref. ^56^). All isotopolog data were corrected for the natural abundance of ^13^C using IsoCor v2 (ref. ^57^).

#### Isotope and carbon switch kinetics

Cells were grown on two transfers of [U-^13^C]-succinate to obtain ^13^C-enriched intracellular metabolites. At mid-exponential growth (OD_600_ of about 0.8–1.0) of the second transfer on [U-^13^C]-succinate, 1.5 ml of ^13^C-labeled cells was filtered on to a 0.22-μm filter, rinsed with an equal volume of sterile minimal-nutrient medium and transferred to an agar plate containing unlabeled succinate, 4HB, VAN or TER. Each condition was conducted in triplicate on separate days with different initial stocks of *C. testosteroni* KF-1 cells (independent biological replicates). Cells adhered to filters and exposed to unlabeled plates were rapidly quenched in 1 ml of cold 2:2:1 methanol:acetonitrile:water after 10 s, 30 s, 1 min, 2 min, 5 min, 15 min and 30 min. Time 0 was quenched immediately after rinsing and before transfer to a plate. After quenching, lysed cells were scraped off of filters, and the solution was centrifuged to remove cell debris and dried under N_2_ as described previously^18^. Incorporation of unlabeled substrate was tracked with LC–MS and analyzed as described above. No adaptive time was required after the substrate switch to the unlabeled substrate to detect incorporation of non-labeled fractions in central carbon metabolites.

#### Single carbon source kinetics

Cells were grown in minimal-nutrient medium containing unlabeled 4HB for two transfers to acclimate the cells. During early exponential growth (OD_600_ of approximately 0.4–6.0) of the second transfer, an aliquot of the unlabeled cells (3 ml) was filtered, rinsed and transferred onto minimal-nutrient agar plates containing unlabeled 4HB. After cells doubled on the plates, the filter with adhered cells was transferred to agar plates containing 100% [^13^C_6_]-(phenyl)-4HB. At specific time points on the ^13^C-labeled plates (15 s, 1 min, 5 min, 15 min and 30 min), the cells were rapidly quenched using the aforementioned solvent solution, and the intracellular metabolites were extracted and analyzed as described above.

### Instrumental analysis of metabolites

LC–MS analysis was conducted on an UHPLC (Thermo Fisher Scientific Dionex UltiMate 3000) coupled to a high-resolution/accurate mass MS (Thermo Fisher Scientific Q Exactive quadrupole-Oribitrap hybrid MS) with electrospray ionization. Metabolomics analysis was conducted on the intracellular and extracellular extracts as described previously^18^. In brief, metabolites were analyzed by reversed-phase ion-pairing LC using an Acquity UPLC Waters 1.7-µm particle size column (2.1 × 100 mm) at a constant column temperature of 25 °C and an injection volume of 10 µl. The mass spectrometer was operated in full-scan negative mode. Metabolite identification was based on accurate mass and matches with standard retention time. For positional ^13^C-labeling analysis, the same LC–MS parameters were used with the addition of data-dependent tandem MS to obtain product ions and map ^13^C-carbons using previously outlined fragmentation pathways^58^. The following data-dependent tandem MS parameters were used: resolution, 17,500; isolation window, 1.0 *m*/*z*; normalized collision energy, 15, 30 and 45; automatic gain control target, 1 × 10^5^; maximum inject time, 50 ms.

Quantification of VAN and PDC from extracellular samples was performed using UHPLC with UV detection at 275 nm (Thermo Scientific Vanquish Flex with diode array detector). The PDC standard was prepared from cultures of *P. putida* CJ251 (refs. ^20,59^) and purified as previously reported^59^.To quantify PDC, we dissolved the standard first in DMSO and then diluted 1:100 to the standard range in high-purity water (Thermo Scientific Barnstead GenPure). A matrix mix spike of PDC into minimal-nutrient medium was used to evaluate the impact of the sample matrix. All other standards were prepared in minimal-nutrient medium. Chromatographic separation was performed using a reversed-phase C18 column (ZORBAX Eclipse Plus, 4.6 × 100 mm, 5 μm; Agilent) with a guard column (4.6 × 12.5 mm, 5 μm; Agilent). Column temperature was maintained at 25 °C. Mobile phases consisted of 1% formic acid in LC–MS-grade water (solvent A) and 80:10:10 acetonitrile:methanol:LC–MS water (solvent B), which was reported to be appropriate for phenolic acid separation^60^. The method described previously^60^ was modified to a total run time of 10.5 min at a flow rate of 0.9 ml min^–1^ and injection volume of 10 µl. The following multistep gradient was used for solvent B: 0 min, 6%; 1 min, 6%; 8.5 min, 25%; 9 min, 25%; 9.5 min, 6%; 10.5 min, 6%.

### Flux ratio analysis

To determine the fractional contribution of OAA versus pyruvate to the PEP pool, we conducted metabolic flux ratio analysis during exponential growth of cells grown on [1-^13^C]-(carboxyl)-4HB. We used the positional ^13^C labeling of citrate determined from fragmentation analysis to estimate the percentage of OAA that was ^13^C labeled at the first or fourth position. We obtained optimized flux ratios by optimizing across three replicate datasets to obtain the minimum error difference between the experimental and simulated labeling data. The following equations were used:

1 $${{{\mathrm{PEP}}}}_0 = {{{\mathrm{f}}}}_1\left( {{{{\mathrm{pyruvate}}}}_0} \right) + {{{\mathrm{f}}}}_2\left( {{{{\mathrm{OAA}}}}_0 + {{{\mathrm{OAA}}}}_{{{{1}}},4{{{\mathrm{th}}}}}} \right)$$

2 $${{{\mathrm{PEP}}}}_1 = {{{\mathrm{f}}}}_1\left( {{{{\mathrm{pyruvate}}}}_1} \right) + {{{\mathrm{f}}}}_2\left( {{{{\mathrm{OAA}}}}_{1,1{{{\mathrm{st}}}}}} \right)$$

Here, f_1_ and f_2_ refer to the fractions of PEP originating from pyruvate and OAA, respectively. The subscript 0 indicates the non-labeled fraction, and the subscript 1 indicates the singly labeled fraction, while 1st and 4th refer to the position of the ^13^C-carbon in OAA.

### Quantitative flux analysis

A metabolic network model consisting of 57 reactions was constructed for ^13^C MFA of *C. testosteroni* KF-1, as described previously^18^. The spontaneous reaction in the 4,5-*meta* pathway was assumed to be at equilibrium between the open and cyclic hemiacetal forms (Fig. 1a)^35^. The biomass efflux rates of metabolite precursors were calculated from experimentally determined growth rates and the stoichiometric ratio of polymer components in biomass composition to metabolites in the model^18^. The stoichiometric molar ratio was next converted to a rate by accounting for the biomass and substrate-specific growth rate. The anabolic demand for NADPH, NADH, and ATP was determined from the growth rates, metabolic network and biomass stoichiometry, as reported previously^18^.

Quantification of metabolic fluxes in CCM during growth on 4HB was conducted on the software OpenFlux2 using an extension of the elementary metabolic unit algorithm to simulate isotopomer balances across parallel-labeling experiments^61^. The ^13^C MFA model was constrained by measured metabolite secretions and biomass effluxes. The TCA cycle oxidative reactions from OAA to succinate were considered irreversible in the model. However, the TCA cycle reactions between succinate and OAA and the three potential cataplerotic reactions (OAA to pyruvate, OAA to PEP and malate to pyruvate) were all considered reversible in the model and were left unconstrained. Optimization of the fluxes was conducted across six isotopomer datasets (two labeling schemes [1-^13^C]-(carboxyl)-4HB and [^13^C_6_]-(phenyl)-4HB and three replicates per labeling scheme) and using the isotopomer distributions of metabolites in the TCA cycle (malate, fumarate, citrate and αKG), EMP pathway (pyruvate, PEP, 3-phosphoglycerate, fructose-6-phosphate and glucose-6-phosphate) and PP pathway (sedoheptulose-7-phosphate). The labeling of CO_2_ was determined experimentally by comparing the ^13^C labeling of *N*-carbamoyl-l-aspartate to aspartate, as demonstrated previously^18^. Addition of ^13^C-labeled carbons from aspartate to *N*-carbamoyl-aspartate was taken as the incorporation of ^13^C-labeled dissolved CO_2_. A global solution was obtained after 100 iterations starting from random initial values for all fluxes and evaluated by assessing the variance-weighted sum of squared residuals between the experimentally determined and model-estimated isotopomer distributions and efflux rates. When the minimized sum of squared residuals values were below the *χ*^2^ statistical test^.^ cutoff at a 95% confidence level, the model was considered to have a statistically acceptable fit. A non-linear search algorithm was used to calculate 95% confidence intervals for the optimized metabolic fluxes^61^. The standard deviation (or individual flux precision) was calculated from the 95% confidence intervals as previously reported^18^.

### Protein production and purification

Mdh (CtesDRAFT_PD1281), ME (CtesDRAFT_PD0934 and CtesDRAFT_PD5092), IDH (CtesDRAFT_PD1820 and CtesDRAFT_PD1824), 4HB 3-monooxygenase (CtesDRAFT_PD2627) and PEP synthase (CtesDRAFT_PD3828) from *C. testosteroni* KF-1 were recombinantly produced in *E. coli* BL21 (DE3) and subsequently purified (Supplementary Fig. 9). The gene fragments encoding the different enzymes were synthesized by Twist Bioscience. The native nucleotide coding sequences from *C. testosteroni* KF-1 were used for all the enzymes except for ME-1 (CtesDRAFT_PD0934), for which the codon usage was optimized for expression in *E. coli* K12 to decrease its GC content. The coding sequences CtesDRAFT_PD0934, CtesDRAFT_PD5092, CtesDRAFT_PD1820 and CtesDRAFT_PD3828 were divided into two gene fragments to ease the synthetic process. The synthesized fragments were amplified by PCR and inserted into a modified pET28a(+) vector by USER cloning^62^, and the corresponding USER primers were designed using the AMUSER tool2. The selected pET28a(+) vector carries a TEV cleavage site instead of the canonical thrombin target sequence between the N-terminal His tag and the multiple cloning site. Except for CtesDRAFT_PD1281, the enzymes were cloned as N-terminal His-tag fusion proteins, and their native start codon was removed. For CtesDRAFT_PD1281, its native start and stop codons were deleted, and the coding sequence was fused with the vector C-terminal His tag. *E. coli* DH5α λpir competent cells were used for plasmid construction. Plasmids were sequence verified and cloned into the expression host *E. coli* BL21 (DE3). The final strains were preserved in 25% glycerol at −80 °C. Supplementary Tables 22 and 23 display the sequences corresponding to the synthetic gene fragments and the USER primers, respectively.

For protein production, transformed *E. coli* BL21 (DE3) cells were grown in 2xYT medium supplemented with 50 μg ml^–1^ kanamycin. Single colonies were used to inoculate precultures, which were grown overnight at 37 °C and 250 r.p.m. The cultures were prepared in 2-liter baffled flasks and incubated at 37 °C and 200 r.p.m. until the OD_600_ reached 0.6–0.8. At this point, cultures were cooled down at 4 °C, and isopropyl-d-1-thiogalactopyranoside was added at a final concentration of 0.4 mM to induce protein expression. After induction, cultures were incubated at 20 °C and 200 r.p.m. for 18 h and then collected by centrifugation (4,000 *g*, 20 min, 4 °C). Cell pellets were resuspended in buffer A (20 mM sodium phosphate (pH 7.5), 300 mM NaCl and 20 mM imidazole), and cell lysis was performed by three passes through an Avestin Emulsiflex C5 French press (ATA Scientific Instruments). Nucleic acids were hydrolyzed by adding 2.5 U ml^–1^ Pierce Universal Nuclease for Cell Lysis (Thermo Fisher Scientific) to the cell extracts, followed by incubation for 30 min at room temperature with gentle shaking. Before purification, the lysates were centrifuged (10,000 *g*, 20 min, 4 °C), and the supernatants were filtered (0.2-μm-pore size).

Purification was performed by immobilized metal affinity chromatography using 1 ml of Ni-NTA resin (HisPur Ni-NTA resin, Thermo Fisher) loaded in 10-ml Pierce disposable columns. The resin was equilibrated with 10 ml of buffer A. The corresponding lysate was passed through the column followed by a washing step with 20 ml of buffer A to remove the non-bound proteins. Elution was performed with 4 ml of buffer B (20 mM sodium phosphate (pH 7.5), 300 mM NaCl and 500 mM imidazole). The buffer of the collected fractions was exchanged using PD-10 desalting columns (Cytiva) according to the instructions of the manufacturer. The new buffer was 20 mM sodium phosphate (pH 7.5) and 300 mM NaCl for all the purified enzymes with the exception of PEPs (CtesDRAFT_PD3828). The proteins were stored at 4 °C with the addition of 0.5 mM DTT until use. For PEPs, the storage buffer was 20 mM HEPES (pH 7.5) and 50 mM NaCl supplemented with 1 mM EDTA and 10 mM DTT to ensure stability^63^; the protein was stored at 4 °C until use.

The concentration of the purified enzymes was determined in a NanoDrop 2000 spectrophotometer using the theoretical extinction coefficient of the respective proteins. The enzyme purity was confirmed by SDS–PAGE, loading 1–2 μg of protein per sample (Supplementary Fig. 10).

### Enzyme assays

Unless otherwise stated, the enzymatic activities were determined following the reduction or oxidation of the NAD(P)^+^/NAD(P)H pair. Samples were prepared in triplicate at a final volume of 200 μl. Reactions were incubated at 30 °C for 8 h with no shaking using an ELx808 plate reader (BioTek Instruments). Absorbance measurements at 340 nm were taken in intervals of 20 s. Absolute consumption or formation of NAD(P)H was determined using the respective standard curve from 0.016 mM to 1 mM NAD(P)H.

#### Cofactor specificity

The cofactor specificity of Mdh (CtesDRAFT_PD1281), ME (CtesDRAFT_PD0934 and CtesDRAFT_PD5092), IDH (CtesDRAFT_PD1820 and CtesDRAFT_PD1824) and 4HB 3-monooxygenase (CtesDRAFT_PD2627) was determined by comparing the activity of the enzymes with both cofactors. For Mdh, the reaction mixture contained 50 mM HEPES (pH 7.5), 10 mM sodium OAA (preparing the stock solution within the same day), 5 mM MgCl_2_ and 0.01% bovine serum albumin (BSA). NADH and NADPH were added at 0.25 mM and 0.5 mM, respectively. The reactions were prepared using 0.0020 μM CtesDRAFT_PD1281. The two MEs (ME-1 and ME-2) were assayed in a reaction mixture containing 50 mM HEPES (pH 7.5), 20 mM sodium l-malate, 5 mM MnCl_2_ and 0.01% BSA. NAD^+^ and NADP^+^ were added at 0.5 mM and 1 mM, respectively. The reactions were performed using 0.040 μM CtesDRAFT_PD0934 or 0.75 μM CtesDRAFT_PD5092. The two IDHs (IDH1 and IDH2) were assayed in a reaction mixture containing 50 mM HEPES (pH 7.5), 10 mM sodium d,l-isocitrate, 5 mM MnCl_2_ and 0.01% BSA. NAD^+^ and NADP^+^ were added at 0.5 mM and 1 mM, respectively. The reactions were prepared using 0.0025 μM CtesDRAFT_PD1820 or 0.030 μM CtesDRAFT_PD1824. For 4HB 3-monooxygenase, the reaction mixture contained 50 mM HEPES (pH 7.5), 10 mM sodium 4HB, 5 mM MgCl_2_ and 0.01% BSA. NADH and NADPH were added at 0.25 mM and 0.5 mM. The reactions were prepared using 0.20 μM CtesDRAFT_PD2627.

#### Allosteric regulation

The effect of the potential allosteric agents on enzyme activity was analyzed relative to a control reaction without the selected compound. For the two MEs, the reaction mixture contained 50 mM HEPES (pH 7.5), 1 mM sodium l-malate, 5 mM MnCl_2_, and 0.01% BSA. The cofactors NADP^+^ and NAD^+^ were used at 0.5 mM for CtesDRAFT_PD0934 and CtesDRAFT_PD5092, respectively. The potential allosteric reagents (l-glutamate, l-aspartate, fumarate, succinate and αKG) were assayed at 0.5 mM and 2 mM. The reactions were prepared using 0.30 μM CtesDRAFT_D0934 or 3.2 μM CtesDRAFT_PD5092. For PEPs (CtesDRAFT_PD3828), the potential inhibitory role of αKG was analyzed. The reaction mixture contained 50 mM HEPES (pH 7.5), 1 mM sodium pyruvate, 1.5 mM ATP, 5 mM MgCl_2_ and 0.01% BSA. The reactions were prepared using 0.20 μM CtesDRAFT_PD3828. The effect of αKG was assayed at 2 mM and 5 mM. Samples were incubated at 30 °C for 30 min with no shaking, and the reaction was stopped by the addition of 5% formic acid. The activity of CtesDRAFT_PD3828 was determined based on the consumption of pyruvate measured on a Dionex UltiMate 3000 HPLC system equipped with an RI‐101 refractive index detector (Shodex). Samples were loaded onto an Aminex HPX‐87X ion exclusion (300 × 7.8 mm) column (Bio-Rad) kept at 30 °C. The mobile phase was 5 mM sulfuric acid at 0.6 ml min^–1^, and an isocratic elution was applied for 11 min. Data processing was performed using the Chromeleon Chromatography Data System software 7.2.9 (Thermo Fisher Scientific).

### Plasmid construction

Plasmids were synthesized by Genscript USA. Plasmids were designed based on the published sequences for *C. testosteroni* KF-1 (NCBI Reference Sequence NZ_AAUJ02000001.1) and either a pBBR1 backbone^64,65^ for autonomous replication or a pk18mobsacB backbone^66^ for chromosomal modification via homologous recombination. For pALC785, the P_tac_ promoter and *pmdAB* genes were synthesized and cloned into pBBR1-MCS1. For pALC786, the P_tac_ promoter and *pmdD* gene were synthesized and cloned into pBBR1-MCS1. For each of these plasmids, the *pmd* genes were codon optimized for expression in *P. putida* to prevent recombination with the native *pmd* locus^67^. For deletion of the chromosomal *pmd* operon, a DNA region 1,058 bp in length upstream of the operon and 1,055 bp downstream of the operon were synthesized together into pK18mobsacB to serve as loci for homologous recombination, resulting in plasmid pALC783. For insertion of a strong promoter in front of the *pmd* operon, DNA was synthesized containing 1,058 bp of DNA homologous to the region upstream of the *pmd* operon followed by the P_tac_ promoter, lacO operator, and a ribosome binding site previously characterized in *P. putida* KT2440 (ref. ^68^), followed by the first 1,008 bp of the coding region. This sequence was cloned into pK18mobsacB, resulting in plasmid pALC784. Synthesized DNAs, plasmid sequences and primers are listed in Supplementary Table 24 and Supplementary Data.

#### Transformation and strain construction in *C. testosteroni*

To enable genetic modifications in *C. testosteroni* KF-1, a transformation protocol was first developed. *C. testosteroni* was grown overnight in 50 ml of LB liquid medium at 30 °C and 250 r.p.m. The following day, stationary-phase cells were collected by centrifugation at 5,000 *g*. The supernatant was removed, and cells were resuspended in 25 ml of 10% glycerol. This wash was repeated two additional times before a final resuspension in 1 ml of 10% glycerol. This suspension was divided into 50-µl aliquots, which were ready for electroporation or frozen at −80 °C for later use. All cell collection and wash steps were performed at room temperature.

For electroporation, 100 ng of plasmid was added to 50 µl of competent cells at room temperature. Cells were electroporated at 1,600 V, 25 µF and 200 Ω in a 1-mm cuvette using a Gene Pulser Xcell (Bio-Rad). Observed time constants were ~4 ms. Cells were immediately recovered in 950 µl of SOC medium and incubated at 30 °C and 250 r.p.m. for 2 h. Cells were plated on LB solid medium (25 g liter^–1^ LB granules (Fisher Scientific) and 15 g liter^–1^ agar) with 50 µg liter^–1^ kanamycin sulfate (Fisher Scientific) to select for transformants. It was observed that some untransformed cells were able to escape selection at concentrations of 50 µg liter^–1^ kanamycin sulfate, so an additional selection was performed at a concentration of 200 µg liter^–1^ kanamycin sulfate by streaking on agar plates to isolate individual colonies that were resistant to this higher level of kanamycin. Successful transformation with the pBBR1-based replicating vectors pALC785 and pALC786 was confirmed by colony PCR (Supplementary Fig. 11), resulting in strains AG9402 and AG9404, respectively. For homologous recombination plasmids, *sacB* was used as a counterselectable marker. After kanamycin selection, cells were plated on YT 20% sucrose plates (10 g liter^−1^ tryptone, 5 g liter^–1^ yeast extract and 200 g liter^–1^ sucrose). Colonies appeared after several days; however, no discernable differences in colony size were observed. Additionally, these colonies remained kanamycin insensitive, even after an additional propagation on YT + 20% sucrose plates, suggesting that no counterselection occurred. Cells were then plated on LB + 10% sucrose plates, and after 24 h, differences in colony size were observed. To create strain AG9493, *C. testosteroni* KF-1 was transformed with pALC783. Deletion of the *pmd* operon was confirmed by colony PCR (Supplementary Fig. 12), and colonies were confirmed to be sensitive to kanamycin concentrations of 200 µg liter^–1^ in both liquid and solid media. For transformation with pALC784, all colonies screened after counterselection showed bands consistent with the wild-type genotype. As an alternative, initial transformed colonies were screened using colony PCR for a single crossover event (after kanamycin selection and before sucrose counterselection) into the downstream region of homology, which would introduce the intended promoter in front of the entire *pmd* operon (Supplementary Fig. 13) as a semistable merodiploid (strain AG9480). Kanamycin selection (200 µg liter^–1^) was maintained during experiments with this strain to ensure that an additional recombination event would not revert the genotype to the wild type. All primers are listed in Supplementary Table 24.

For future genetic modification of *C. testosteroni* KF-1, use of 200 µg liter^–1^ kanamycin rather than 50 µg liter^–1^ in liquid and solid media is recommended to ensure elimination of cells without *kanR*. Electroporation of pALC783-786 was repeated using 200 µg liter^–1^ kanamycin for selection and resulted in transformation efficiencies of 16 and 120 colony-forming units per µg of DNA for the homologous recombination plasmids pALC783 and pALC784, respectively, and 154,000 and 176,000 colony-forming units per µg of DNA for the autonomously replicating plasmids pALC785 and pALC786, respectively. For counterselection using *sacB*, LB + 10% sucrose plates are recommended, as they produced clear phenotypic differences in transformants after overnight incubation.

### Statistical analysis

Physiology experiments were analyzed for statically significant differences (*P* ≤ 0.05) between growth conditions using one-way analysis of variance (ANOVA) combined with Tukey honestly significant difference (HSD) post hoc tests. The Pearson correlation coefficient (*r*) was calculated to determine the strength of the relationship between transcriptomics and proteomics and between fluxomics and transcriptomics, proteomics or substrate-to-product ratios. A two-sided *t*-test was used to assess the significance of the relationships. For proteomics analysis, statistically significant differential protein abundance between aromatic-grown cells and succinate-grown cells was determined by calculating a *z* score for protein abundance differences as described previously^18^. In brief, assuming a standard normal distribution, *z-*score values were translated to *P* values and further corrected using the *q* value method (controlled to 0.05) to correct for multiple-testing familywise error rate.

### Reporting summary

Further information on research design is available in the Nature Portfolio Reporting Summary linked to this article.

## Online content

Any methods, additional references, Nature Portfolio reporting summaries, source data, extended data, supplementary information, acknowledgements, peer review information; details of author contributions and competing interests; and statements of data and code availability are available at 10.1038/s41589-022-01237-7.

### Supplementary information

Supplementary Information Supplementary Figs. 1–13 and Tables 1–24.

Reporting Summary

Supplementary Data 1 DNA synthesis and plasmid sequences for plasmid construction and statistical information for Fig. 1b,c.

## Data Availability

Proteomic mass spectral data are available via ProteomeXchange under accession number PXD029813 and the MassIVE repository (massive.ucsd.edu) under accession number MSV000088418. Stable isotope-assisted metabolomics LC–HRMS data are deposited in the MetaboLights repository (www.ebi.ac.uk/metabolights/) under accession number MTBLS3947. The RNA-sequencing datasets generated during this study are available at the Gene Expression Omnibus with accession number GSE192852. The NCBI Reference Sequence for *C. testosteroni* KF-1 was NZ_AAUJ02000001.1.
