# Author Correction: Complex regulation in a *Comamonas* platform for diverse aromatic carbon metabolism

**DOI:** 10.1038/s41589-024-01561-0

**Published:** 2024-02-05

**Authors:** Rebecca A. Wilkes, Jacob Waldbauer, Austin Carroll, Manuel Nieto-Domínguez, Darren J. Parker, Lichun Zhang, Adam M. Guss, Ludmilla Aristilde

**Affiliations:** 1 Department of Biological and Environmental Engineering, College of Agriculture and Life Sciences, Cornell University, Ithaca, NY USA; 2 Department of Civil and Environmental Engineering, McCormick School of Engineering and Applied Science, Northwestern University, Evanston, IL USA; 3 Department of the Geophysical Sciences, University of Chicago, Chicago, IL USA; 4 Biosciences Division, Oak Ridge National Laboratory, Oak Ridge, TN USA; 5 The Novo Nordisk Foundation Center for Biosustainability, Technical University of Denmark, Kongens Lyngby, Denmark; 6 Northwestern Center for Synthetic Biology, Evanston, IL USA

**Keywords:** Metabolic pathways, Metabolic engineering, Proteomics, Metabolomics

Correction to: *Nature Chemical Biology* 10.1038/s41589-022-01237-7, published online 6 February 2023.

In the version of this article initially published, the surname of Austin Carroll was misspelled (Caroll). In the “Allosteric regulation influences key metabolic reactions” section, in the sentence now reading, in part, “only high concentrations of αKG had a significant inhibitory impact on ME-2,” the word “inhibitory” was missing. In Fig. 3b, the carbon mapping illustration of citrate and αKG was drawn incorrectly. In Extended Data Fig. 1, “Malate” appeared incorrectly as “Malat”. No changes were needed in the data presented in Fig. 3 or Extended Data Fig. 1b, and the conclusions in the article remain the same. The errors have been corrected in the HTML and PDF versions of the article.

