# Supplementary material for: Complex regulation in a Comamonas platform for diverse aromatic carbon metabolism
Source: Nat Chem Biol. 2023 Feb 6;19(5):651–62. doi: 10.1038/s41589-022-01237-7 (PMC10154247; doi:10.1038/s41589-022-01237-7)
Supplement: Supplementary file 1 — Supplementary Figs. 1–13 and Tables 1–24. [file 41589_2022_1237_MOESM1_ESM.pdf]

nature chemical biology

Article https://doi.org/10.1038/s41589-022-01237-7

Complex regulation in a *Comamonas* platform for diverse aromatic carbon metabolism

In the format provided by the authors and unedited

TABLE OF CONTENTS

Supplementary Fig. 1 | Extended data for Fig. 2a. 3

Supplementary Fig. 2 | Reductive flux from OAA contributes to fumarate pool. 4

Supplementary Fig. 3 | Goodness of fit for the ^{13}C-metabolic flux analysis 4

Supplementary Fig. 4 | Reaction profiles at 340 nm for the two malic enzymes. 5

Supplementary Fig. 5 | Allosteric regulation of PEP synthase. 6

Supplementary Fig. 6 | Differential gene and protein expression relative to 4HB. 6

Supplementary Fig. 7 | Growth rate of the three overexpression strains. 7

Supplementary Fig. 8 | Extended data for Fig. 6a. 8

Supplementary Fig. 9 | Main activities of the selected enzymes from *C. testosteroni*. 9

Supplementary Fig. 10 | Sodium dodecyl sulfate–polyacrylamide gel electrophoresis (SDS–PAGE) of purified proteins. 10

Supplementary Fig. 11 | Construction of strain AG9402 and AG9404. 11

Supplementary Fig. 12 | Construction of strain AG9493. 12

Supplementary Fig. 13 | Construction of strain AG9480. 13

Supplementary Table 1 | Physiological characteristics. 14

Supplementary Table 2 | Differential gene expression in initial catabolism pathways to protocatechuate. 14

Supplementary Table 3 | Changes in protein abundance for the enzymes in the initial catabolism pathways to protocatechuate 15

Supplementary Table 4 | Differential gene expression in the three putative protocatechuate cleavage pathways 16

Supplementary Table 5 | Changes in protein abundance for the enzymes in the three putative protocatechuate cleavage pathways. 17

Supplementary Table 6 | Protein sequence identity of ortho cleavage pathway enzymes annotated in the *C. testosteroni* and *P. putida* genomes 18

Supplementary Table 7 | Protein sequence identity of 2,3-meta cleavage pathway enzymes annotated in the *C. testosteroni* and *Paenibacillus* sp. JJ-1b genomes 18

Supplementary Table 8 | Protein sequence identity of 4,5-meta cleavage pathway enzymes annotated in the *C. testosteroni* and two previously characterized species............................19

Supplementary Table 9 | Sequence similarity between the putative 4-hydroxybenzoate transporter. 20

Supplementary Table 10 | Differential gene expression in central carbon metabolism. 20

Supplementary Table 11 | Changes in protein abundance for the enzymes in central carbon metabolism. 22

Supplementary Table 12 | Free energy calculated from the ^{13}C-metabolic flux analysis. 24

Supplementary Table 13. Fragmentation analysis of singly labeled citrate and relative intensity (Rel. Int.) of product ions. 24

Supplementary Table 14 | Intracellular metabolic flux rates. 25

Supplementary Table 15 | *In vitro* calculation of cofactor specificities for ME, MaeB, IDH2, IDH1, Mdh, and PobA from *C. testosteroni* KF-1. 26

Supplementary Table 16 | Protein sequence identity comparison of PobA. 27

Supplementary Table 17 | Quantitative determination of NADH/FADH2, NADPH, and ATP production and consumption. 28

Supplementary Table 18 | Comparison of transcriptomics, proteomics, and fluxomics. 28

Supplementary Table 19 | The Log_2 fold change in the ratio of substrate (S) to product (P) of quantified intracellular metabolites and ^{13}C-metabolic fluxes. 30

Supplementary Table 20 | Measured allosteric regulation of ME (CtesDRAFT_PD0934) in the presence of five potential effectors. 31

Supplementary Table 21 | Measured allosteric regulation of MaeB (CtesDRAFT_PD5092) in the presence of five potential effectors. 31

Supplementary Table 22 | Nucleotide sequences of synthetic gene fragments encoding the enzymes studied in this work. 32

Supplementary Table 23 | Nucleotide sequences of primers used for plasmid construction. .35

Supplementary Table 24 | Primer sequences used for strain engineering. 37

SUPPLEMENTARY FIGURES

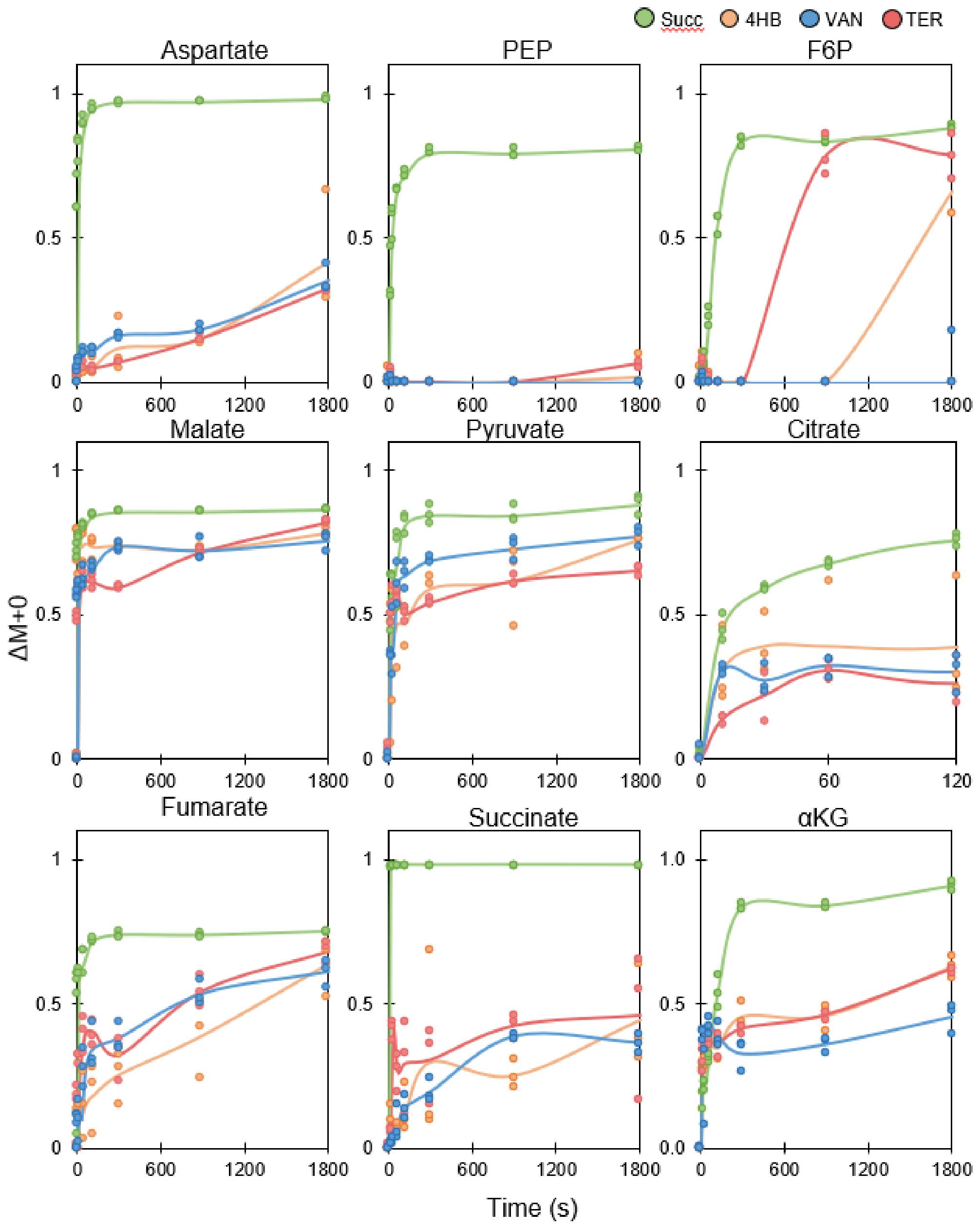

Supplementary Fig. 1 | Extended data for Fig. 2a. Experimental kinetic incorporation of nonlabelled fraction (M+0) over 1800 s (30 min) after carbon switch from ^{13}C-succinate to unlabeled succinate (green), 4HB (orange), VAN (blue), or TER (red). Labeling data were from biological replicates (n = 3) shown as individual data points. The line through the points denotes the average of the replicates. Metabolite abbreviations: phosphoenolpyruvate, PEP; fructose 6-phosphate, F6P; α-ketoglutarate, αKG.

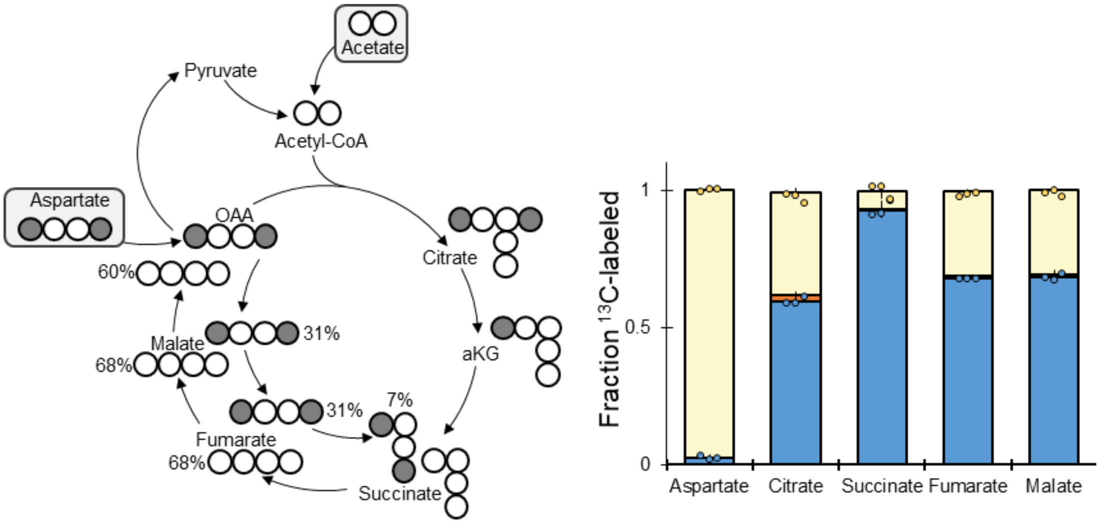

Supplementary Fig. 2 | Reductive flux from OAA contributes to fumarate pool. Carbon mapping (left) and experimental data (right) from long-term isotopic enrichment for *C. testosteroni* KF-1 cells grown on [1,4-$^{13}C_2$]-aspartic acid and unlabeled acetate. For the diagram, ^{13}C-carbons are in grey; ^{12}C-carbons are in white. Metabolite labeling patterns: M+0 (light blue), M+1(orange), and M+2 (cream). Labeling data (mean ± standard deviation) were from biological replicates (n = 3).

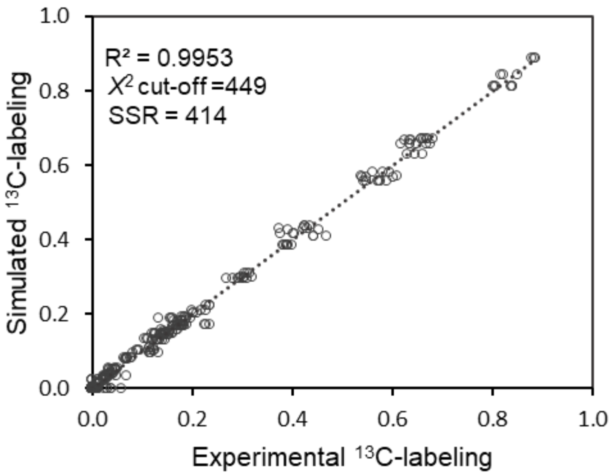

Supplementary Fig. 3 | Goodness of fit for the ^{13}C-metabolic flux analysis. The data show the correlation between the mass isotopomers of the ^{13}C-labeling experiments and the corresponding model-optimized simulated values. The experimental data for each strain were from two parallel tracer experiments [1-^{13}C]-(carboxyl)-4HB and [$^{13}C_6$]-(phenyl)-4HB. Optimized fluxes are shown in Fig. 6 and Table S10.

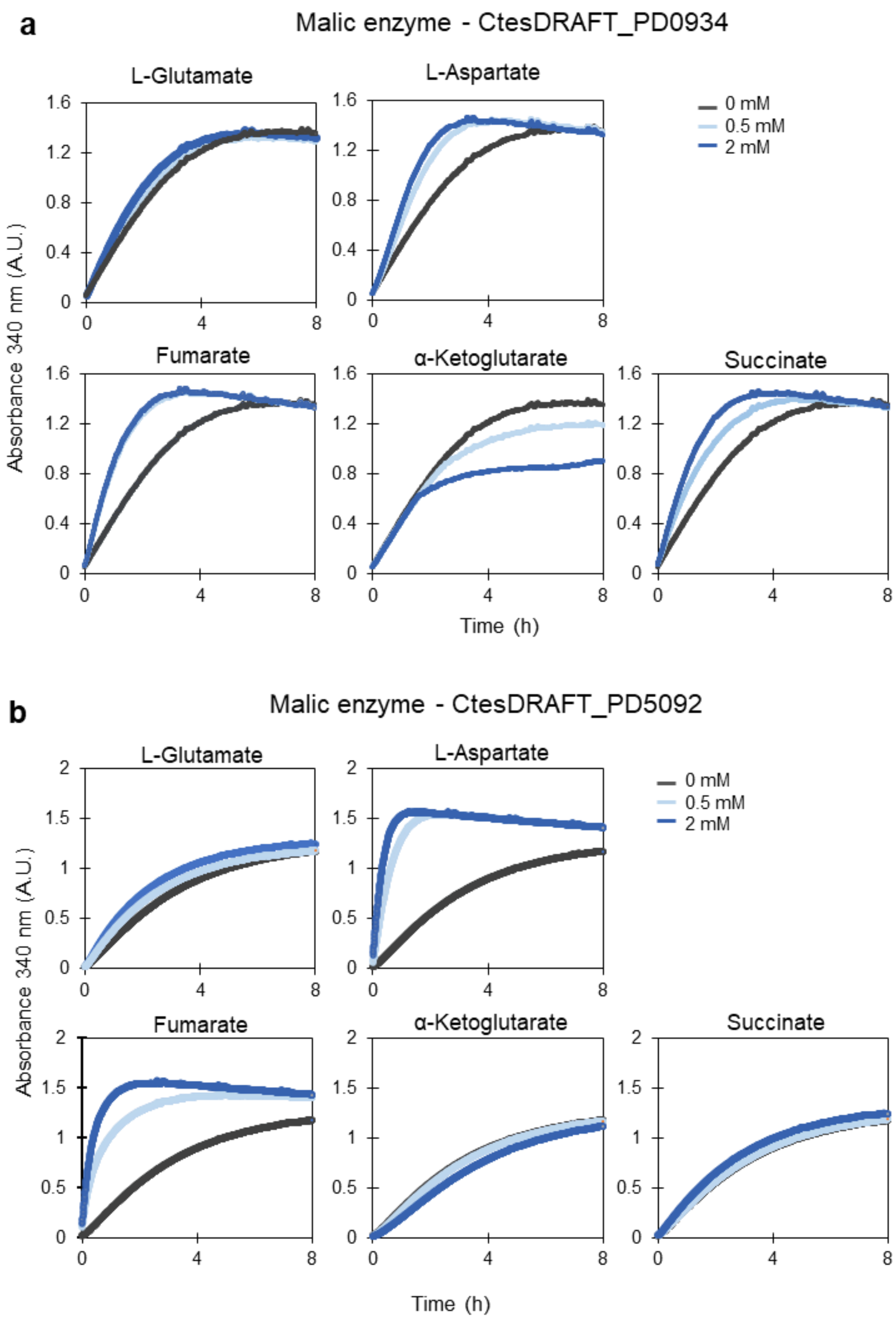

Supplementary Fig. 4 | Reaction profiles at 340 nm for the two malic enzymes. a) ME (CtesDRAFT_PD0934) and **b)** MaeB (CtesDRAFT_PD5092) in the presence of potential allosteric regulators (effectors): glutamate, aspartate, fumarate, α-ketoglutarate, or succinate. The control without an effector is shown in black, 0.5 mM of added effector is in light blue, and 2 mM of added effector is in dark blue. Measurements were taken every 20 s. Data points are shown separately for each replicate (n=3)

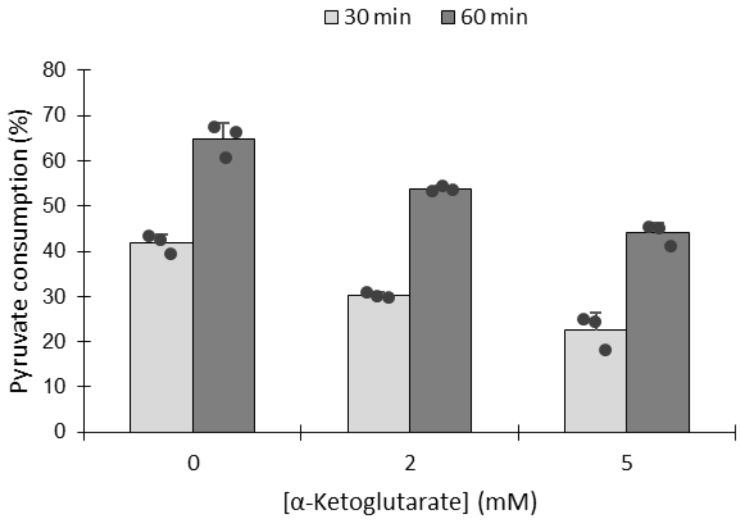

Supplementary Fig. 5 | Allosteric regulation of PEP synthase. Percentage of pyruvate consumption after 30 min (light grey) and 60 min (dark grey) for PEP synthase (CtesDRAFT_PD3828) without or with the addition of α-ketoglutarate. Data are presented as mean ± standard deviation of three biological replicates (n = 3).

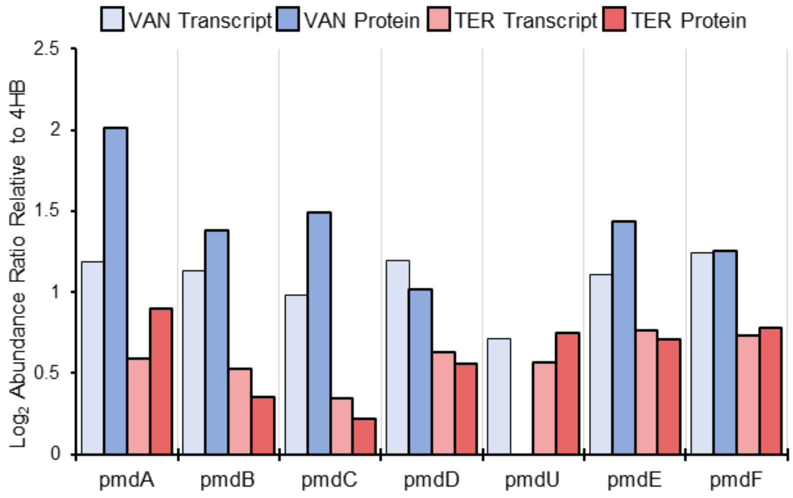

Supplementary Fig. 6 | Differential gene and protein expression relative to 4HB. Differences in the 4,5-*meta* cleavage pathway gene expression and protein abundance when cells were grown on VAN (shades of blue) and TER (shades of red) relative to 4HB. Values are expressed as the Log_2 fold changes between growth conditions for triplicate measurements of transcriptomics data and quadruplicate measurements of proteomics data.

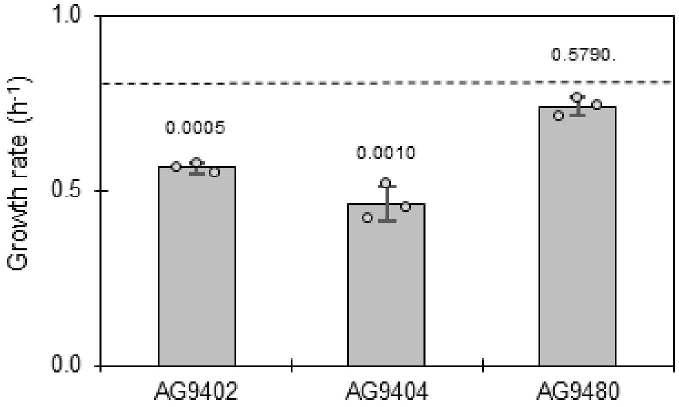

Supplementary Fig. 7 | Growth rate of the three overexpression strains. Cells were grown on 100 mM C 4HB. Data are presented as mean ± standard deviation of three biological replicates (n = 3). Statistically significant differences from the wildtype strain of *C. testosteroni* KF-1 were determined using two-tailed unpaired *t* test.

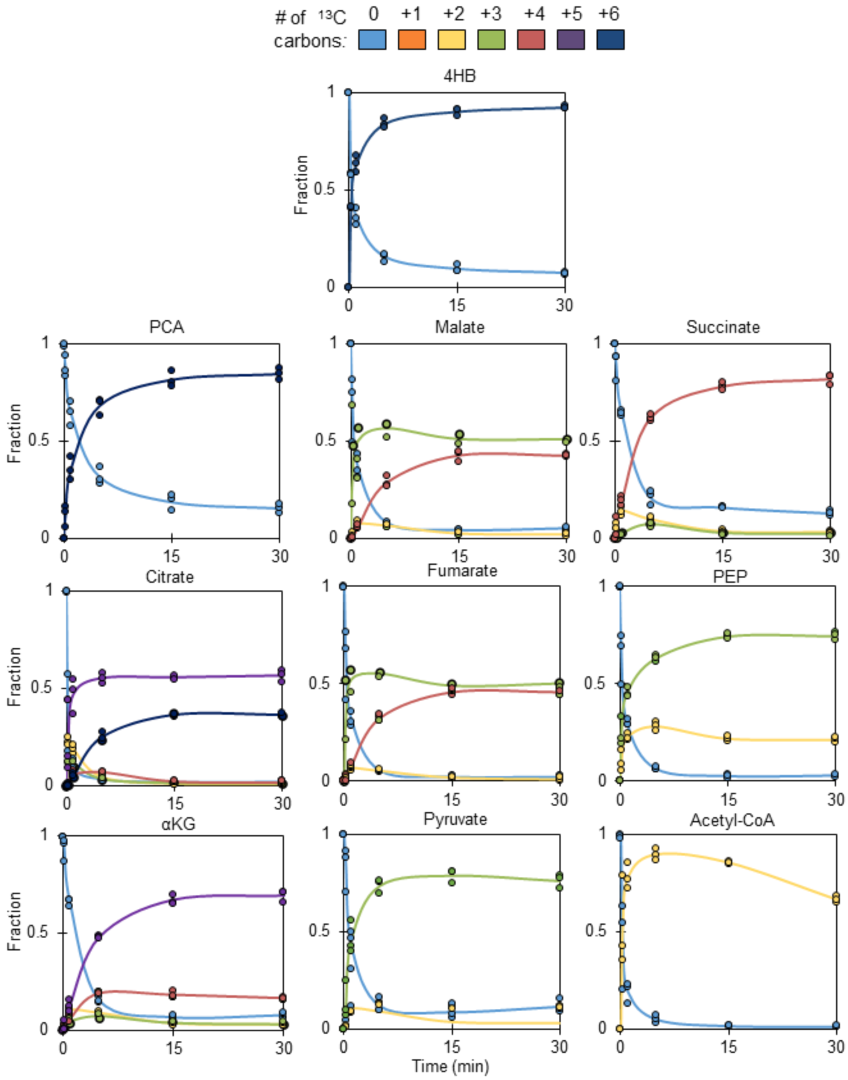

Supplementary Fig. 8 | Extended data for Fig. 6a. Kinetics of 4HB assimilation into central carbon metabolism in *C. testosteroni* KF-1 during growth on [$^{13}C_6$]-(phenyl)-4HB over 30 minutes. Labeling data were from biological replicates (n = 3) shown as individual data points. The line through the points denotes the average of the replicates. Abbreviations are as follows: 4-hydroxybenzoate, 4HB; protocatechuate, PCA; phosphoenolpyruvate, PEP; α-ketoglutarate, αKG.

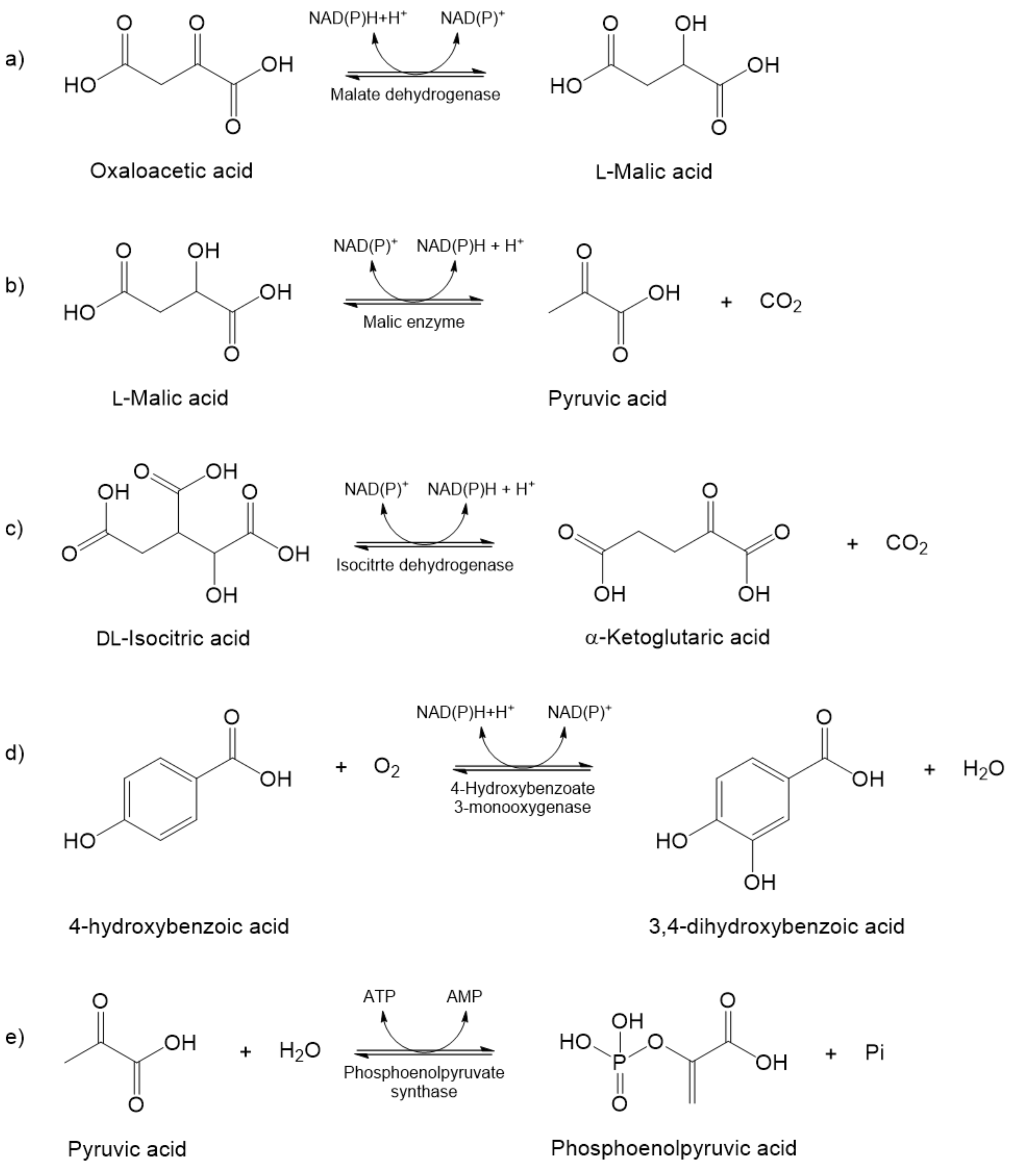

Supplementary Fig. 9 | Main activities of the selected enzymes from *C. testosteroni*.
Malate dehydrogenase (a); malic enzyme (b); isocitrate dehydrogenase (c); 4-hydroxybenzoate 3-monooxygenase (d); phosphoenolpyruvate synthase (e).

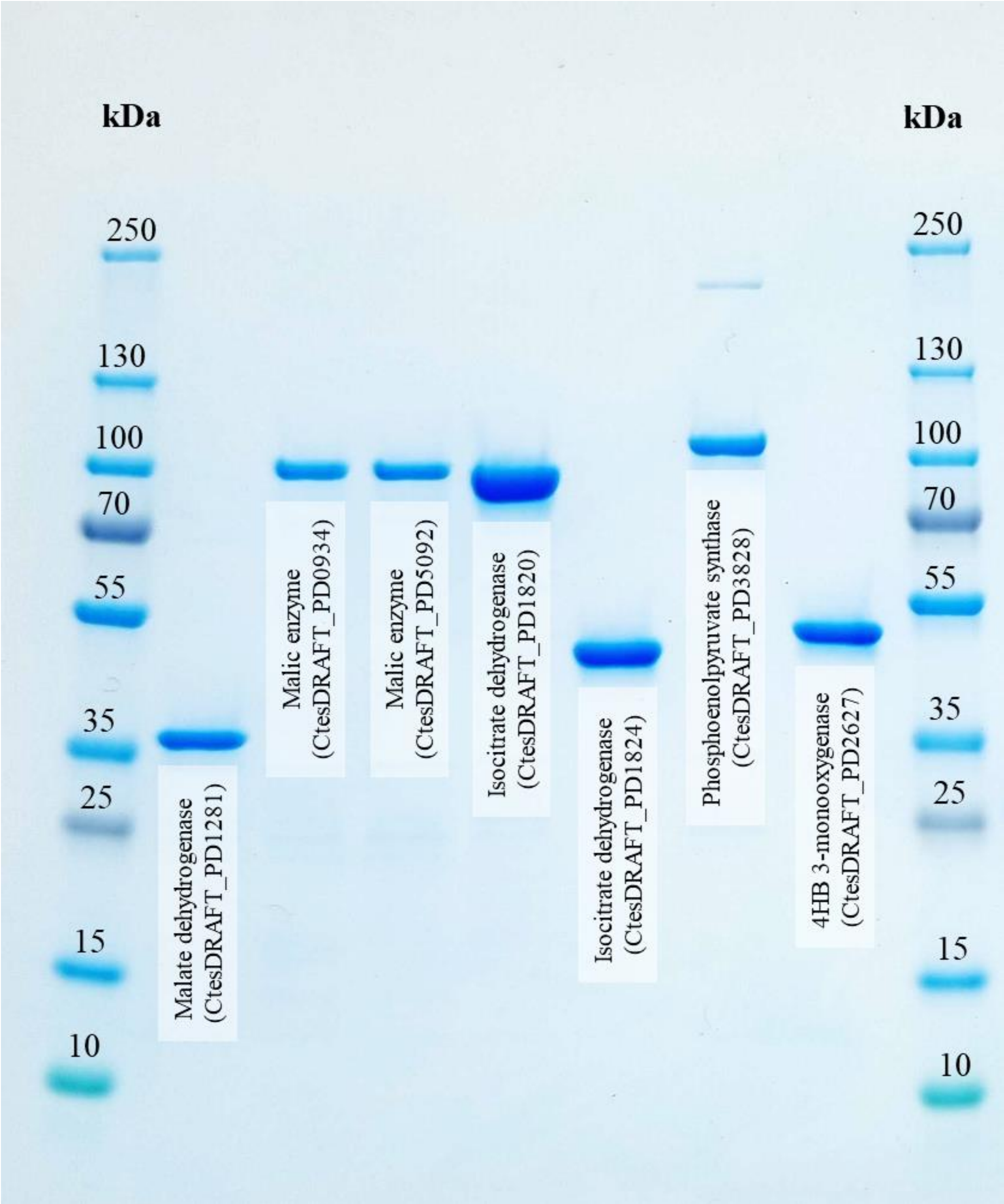

Supplementary Fig. 10 | Sodium dodecyl sulfate–polyacrylamide gel electrophoresis (SDS–PAGE) of purified proteins. Proteins were used in experiments to determine cofactor specificities and allosteric regulation.

a b

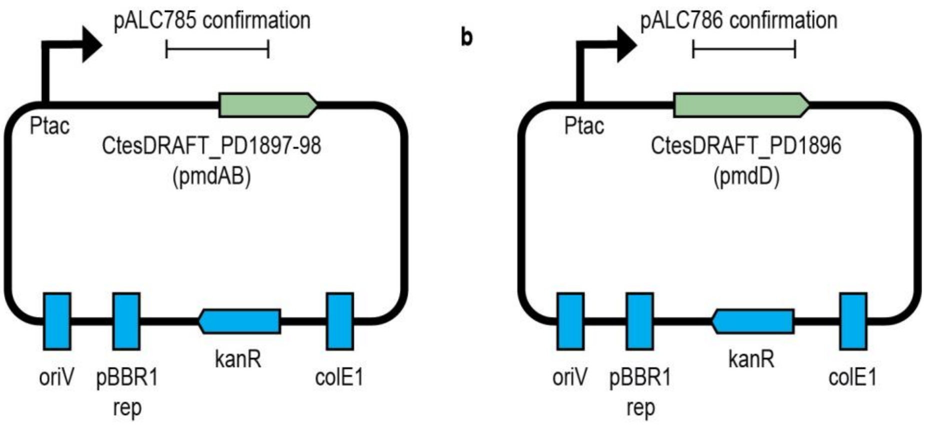

c

PCR reaction	primer 1	primer 2	expected band in AG9402 or AG9404	annealing temperature
pALC785 confirmation	oALC833	oALC834	585 bp	62˚C
pALC786 confirmation	oALC831	oALC832	650 bp	62˚C

d

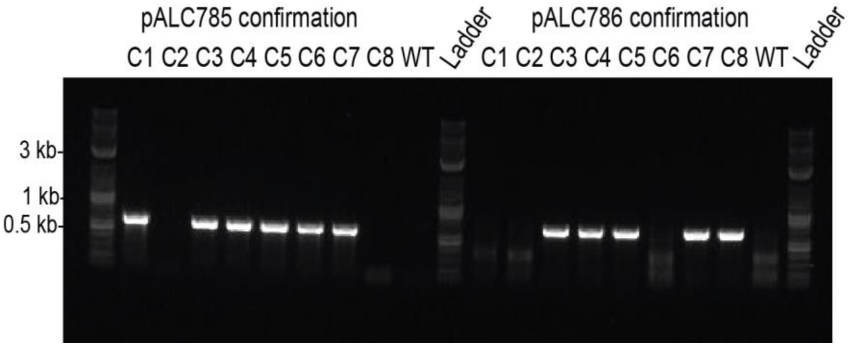

Supplementary Fig. 11 | Construction of strain AG9402 and AG9404. Autonomously replicating plasmids (A) pALC785 and (B) pALC786 were transformed into Comamonas testosteroni KF-1 to create strains AG9402 and AG9404, respectively. (C) Colony PCR primers and conditions used to verify AG9402 and AG9404 construction. All PCR reactions for strain verification were carried out using Phusion High-Fidelity DNA Polymerase (NEB M0530L). (D) Gel electrophoresis of reactions in (C), including 8 colonies after kanamycin selection, as well as a Wildtype (WT) cell control. Reactions were run on a 1% agarose TAE gel and a 1 kb Plus DNA Ladder (NEB-N3200L) was included for quantifying band sizes. Colony 1 and colony 3 were saved as strains AG9402 and AG9404, respectively.

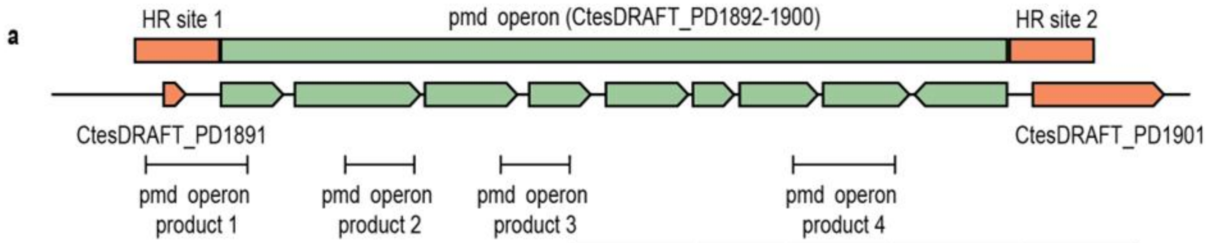

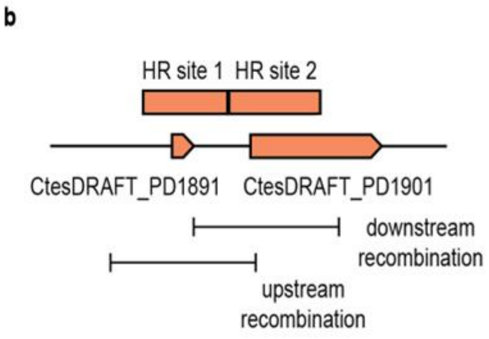

c

PCR reaction	primer 1	primer 2	expected band in AG9493	annealing temperature
upstream recombination	oALC874	oALC880	1262 bp	64˚C
downstream recombination	oALC840	oALC875	1271 bp	67˚C
pmd operon product 1	oALC874	oALC841	0 bp (1346 bp - WT)	67˚C
pmd operon product 2	oALC882	oALC883	0 bp (593 bp – WT)	67˚C
pmd operon product 3	oALC884	oALC885	0 bp (742 bp – WT)	67˚C
pmd operon product 4	oALC886	oALC887	0 bp (1154 bp – WT)	67˚C

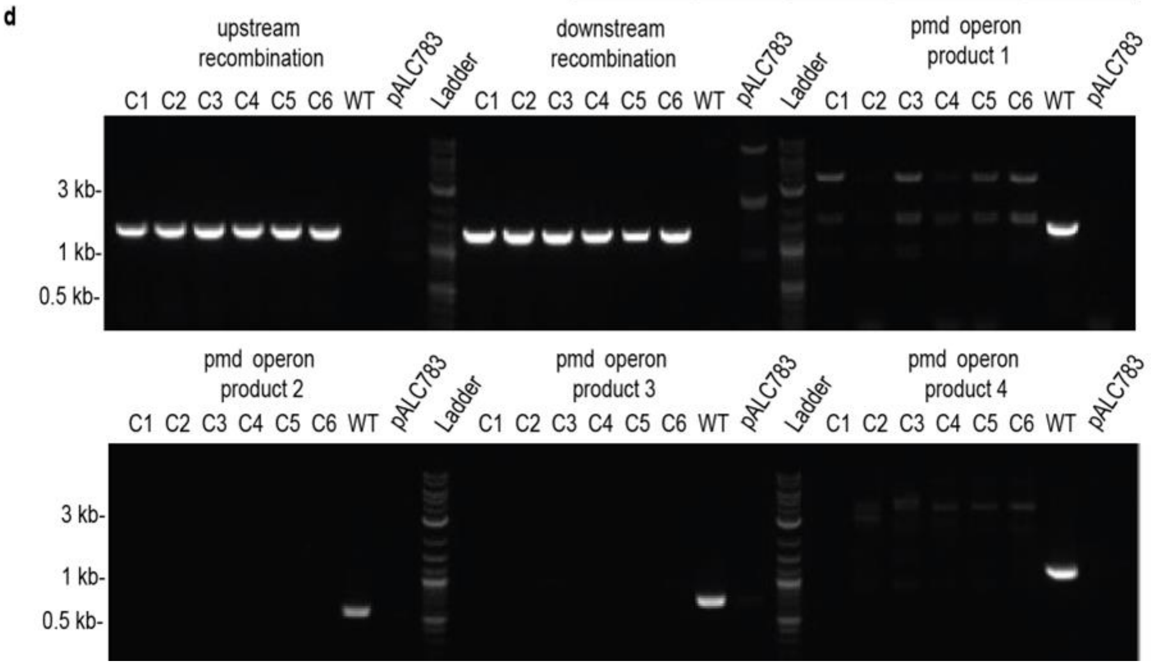

Supplementary Fig. 12 | Construction of strain AG9493. The *pmd* operon locus in (A) WT and (B) AG9493 strains, including homologous recombination sites (HR-orange) for pALC783, *pmd* pathway genes (green, not to scale), and amplicons for PCR confirmation. (C) Colony PCR primers and conditions used to verify AG9493 construction. (D) Gel electrophoresis of reactions in (C), including six colonies after sucrose counter selection, as well as a wildtype (WT) cell control and a pALC783 plasmid control. Reactions were run on a 1% agarose TAE gel and a 1 kb Plus DNA Ladder (NEB-N3200L) was included for quantifying band sizes. A wildtype band is not expected in the first two PCR reactions because it would be too large. Colony C1 was saved as strain AG9493.

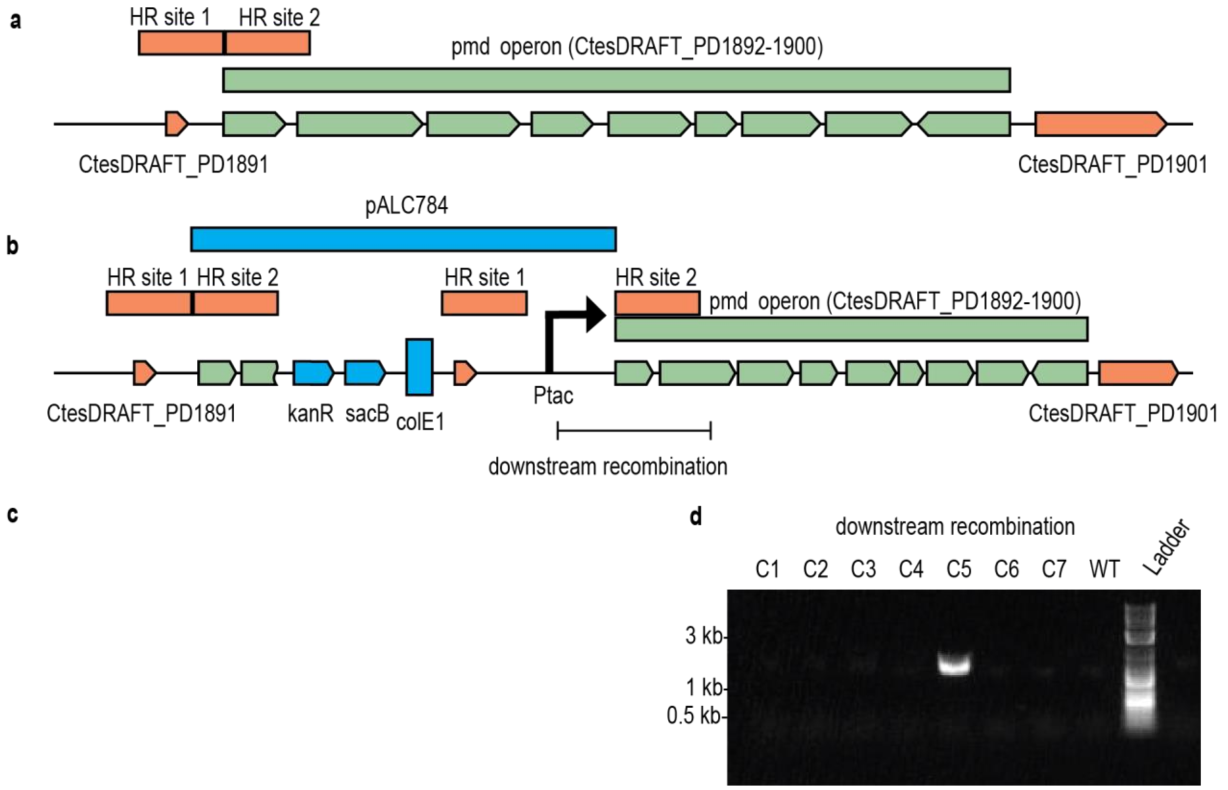

Supplementary Fig. 13 | Construction of strain AG9480. The pmd operon locus in (A) WT and (B) AG9480 strains, including homologous recombination sites (HR-orange rectangle) for pALC784, pmd pathway genes (green), a gene upstream of the pmd operon (CtesDRAFT_PD1891, orange arrow), pBBR1 vector components (blue), and amplicon for PCR confirmation. Strain AG9480 is the product of a single recombination event of pALC784 at HR site 2 in the WT strain. This recombination results in an additional copy of genes CtestDRAFT_PD1891 and 1892 due to their inclusion in HR sites 1 and 2, as well the insertion of Ptac promoter upstream of the pmd operon. (C) Colony PCR primers and conditions used to verify AG9480 construction. (D) Gel electrophoresis of reaction in (C), including seven colonies after kanamycin selection, as well as a wildtype (WT) cell control. Reactions were run on a 1% agarose TAE gel and a 1 kb Plus DNA Ladder (NEB-N3200L) was included for quantifying band sizes. Colony C5 was saved as strain AG9480.

SUPPLEMENTARY TABLES

Supplementary Table 1 | Physiological characteristics. Data represent mean ± standard deviation of *C. testosteroni* KF-1 grown in triplicate on 4-hydroxybenzoate, vanillate, and terephthalate.

	4-hydroxybenzoate	Vanillate	Terephthalate
Growth rate (h^{-1})	0.76 ± 0.03	0.48 ± 0.04	0.56 ± 0.03
Consumption rate (mmol g_{CDW}^{-1} h^{-1})	11.9 ± 1.1	5.1 ± 0.6	5.5 ± 0.9
Cumulative secretions (mmol C g_{CDW}^{-1} h^{-1})	10.9 ± 1.3	0.04 ± 0.01	0.25 ± 0.06

Supplementary Table 2 | Differential gene expression in initial catabolism pathways to protocatechuate. Values represent fold changes (FC) in transcript abundance for *C. testosteroni* KF-1 cells grown on aromatic compounds relative to cells grown on succinate. ND, transcript not detected. Statistically significant differential transcript abundance was determined using Degust software package [53, main text]

Gene Names	Locus Tag	Protein	Transcript abundance Log_2(FC) (4HB/SUCC)		(VAN/SUCC)		(TER/SUCC)	
			Average	P-values	Average	P-values	Average	P-values
pmdK	CtesDRAFT_PD1893	4HB transporter/MFS-transporter/protocatechuate transporter	5.103	3.09E-17	6.179	3.38E-18	6.077	3.97E-18
pobA	CtesDRAFT_PD2627	4HB 3-monooxygenase	7.655	3.84E-14	5.082	3.98E-12	0.536	4.15E-02
vanK	CtesDRAFT_PD0402	VAN transporter	1.061	3.53E-06	8.587	2.70E-17	0.997	9.87E-06
vanA1	CtesDRAFT_PD0400	VAN O-demethylase oxygenase subunit	-0.233	2.82E-03	7.458	2.34E-20	0.662	9.58E-08
vanA2	CtesDRAFT_PD0403	VAN O-demethylase oxidoreductase	0.923	7.45E-08	8.744	6.50E-20	1.133	8.32E-09
vanB	CtesDRAFT_PD0404	VAN O-demethylase oxidoreductase	1.333	7.64E-08	8.569	1.01E-17	0.902	1.00E-05
tphC	CtesDRAFT_PD2131	TER permease	0.203	6.71E-02	1.136	3.46E-08	10.265	8.80E-20
tctA	CtesDRAFT_PD4157	TER transporter	-3.022	3.98E-16	0.969	5.88E-10	2.853	2.55E-15
tctB	CtesDRAFT_PD4158	TER transporter	-3.057	1.83E-15	0.911	8.89E-10	2.893	1.50E-15
tphA2	CtesDRAFT_PD2130	TER 1,2-dioxygenase oxygenase component large subunit	ND		ND		9.18	1.10E-20
tphA3	CtesDRAFT_PD2129	TER 1,2-dioxygenase oxygenase component small subunit	ND		ND		9.762	2.67E-19

tphA1	CtesDRAFT_PD2127	TER 1,2-dioxygenase reductase	ND		ND		7.29	7.39E-18
tphB	CtesDRAFT_PD2128	TER dihydrodiol dehydrogenase	0.229	1.23E-02	0.709	5.45E-07	9.391	7.66E-21

Supplementary Table 3 | Changes in protein abundance for the enzymes in the initial catabolism pathways to protocatechuate. Values represent fold changes (FC) in protein abundance for *C. testosteroni* KF-1 cells grown on aromatic compounds relative to cells grown on succinate. Statistically significant differential protein abundance was determined by calculating a *Z*-score for protein abundance differences, assuming a standard normal distribution, and translating *Z*-score values to *P*-values. These *P*-values were further corrected using the *q*-value method (controlled to 0.05) to correct for multiple testing familywise error rate. ND, protein not detected. Present, protein found during growth on the aromatic compound but not succinate.

Gene Names	Locus Tag	Protein	Protein abundance Log_2(FC)					
			(4HB/SUCC)		(VAN/SUCC)		(TER/SUCC)	
			Average	P-values	Average	P-values	Average	P-values
PmdK	CtesDRAFT_PD1893	4HB transporter/MFS-transporter/protocatechuate transporter	Present		Present		Present	
PobA	CtesDRAFT_PD2627	4HB 3-monooxygenase	6.369	2.83E-07	4.746	0.015	0.402	0.378
VanK	CtesDRAFT_PD0402	VAN transporter	ND		ND		ND	
VanA1	CtesDRAFT_PD0400	VAN O-demethylase oxygenase subunit	ND		Present		ND	
VanA2	CtesDRAFT_PD0403	VAN O-demethylase oxidoreductase	ND		ND		ND	
VanB	CtesDRAFT_PD0404	VAN O-demethylase oxidoreductase	ND		ND		ND	
TphC	CtesDRAFT_PD2131	TER permease	ND		Present		Present	
TctA	CtesDRAFT_PD4157	TER transporter	-1.149	0.148466	0.48	0.36	2.884	0.003
TctB	CtesDRAFT_PD4158	TER transporter	ND		ND		ND	
TphA2	CtesDRAFT_PD2130	TER 1,2-dioxygenase oxygenase component large subunit	ND		ND		Present	
TphA3	CtesDRAFT_PD2129	TER 1,2-dioxygenase oxygenase component small subunit	ND		ND		Present	
TphA1	CtesDRAFT_PD2127	TER 1,2-dioxygenase reductase	ND		ND		Present	
TphB	CtesDRAFT_PD2128	TER dihydrodiol dehydrogenase	ND		ND		Present	

Supplementary Table 4 | Differential gene expression in the three putative protocatechuate cleavage pathways. Values represent fold changes (FC) in transcript abundance for *C. testosteroni* KF-1 cells grown on aromatic compounds relative to cells grown on succinate. Grey boxes are for genes that were not found in the *Comamonas testosteroni* KF-1 genome. Statistically significant differential transcript abundance was determined using Degust software package [53, main text].

Gene Names	Locus Tag	Protein	Transcript abundance Log_2(FC) (4HB/SUCC)		(VAN/SUCC)		(TER/SUCC)	
			Average	P-values	Average	P-values	Average	P-values
pmdA	CtesDRAFT_PD1897	PCA 4,5-dioxygenase, alpha subunit	4.713	1.41E-18	5.989	9.49E-20	5.814	9.49E-20
pmdB	CtesDRAFT_PD1898	PCA 4,5-dioxygenase, beta subunit	4.733	9.06E-19	5.956	7.39E-20	5.771	7.39E-20
pmdC	CtesDRAFT_PD1899	CHMS dehydrogenase	4.318	6.54E-18	5.392	5.89E-19	5.173	5.89E-19
pmdD	CtesDRAFT_PD1896	PDC hydrolase	4.715	2.66E-18	6.004	1.88E-19	5.856	1.88E-19
pmdU	CtesDRAFT_PD1892	OMA tautomerase	4.696	4.63E-14	5.515	6.57E-15	5.78	6.57E-15
pmdE	CtesDRAFT_PD1894	OMA hydratase	4.996	6.86E-19	6.195	6.67E-20	6.268	6.67E-20
pmdF	CtesDRAFT_PD1895	CHA aldolase	4.883	1.30E-18	6.218	8.80E-20	6.121	8.80E-20
pcaH	CtesDRAFT_PD0424	PCA 3,4-dioxygenase, beta subunit	-0.043	7.25E-01	-0.006	9.62E-01	-0.409	9.62E-01
pcaG	NF	PCA 3,4-dioxygenase alpha subunit						
pcaB	CtesDRAFT_PD5238	3-carboxy-cis,cis-muconate cycloisomerase	0.04	8.09E-01	0.998	1.71E-05	0.505	1.71E-05
pcaC	CtesDRAFT_PD5237	CML decarboxylase	0.357	7.54E-04	0.548	1.41E-05	-0.024	1.41E-05
pcaD	CtesDRAFT_PD4384	βKAP enol-lactonase	-2.591	7.38E-09	0.307	1.18E-02	1.886	1.18E-02
pcaI	CtesDRAFT_PD5468	3-oxoadipyl-CoA-transferase	-0.231	1.40E-01	-0.054	7.11E-01	0.152	7.11E-01
pcaJ	CtesDRAFT_PD1882	3-oxoadipyl-CoA-transferase	-0.107	4.20E-01	0.164	2.10E-01	-0.461	2.10E-01
pcaF	CtesDRAFT_PD4382	3-oxoadipyl-CoA thiolase	-1.838	8.12E-10	0.944	2.81E-08	2.259	2.81E-08
praA	NF	PCA 2,3-dioxygenase						
praH	NF	5CHMS decarboxylase						
praB	CtesDRAFT_PD5289	HMS dehydrogenase	-0.747	4.54E-04	0.517	1.68E-03	0.351	1.68E-03
praC	CtesDRAFT_PD5334	OCA tautomerase	0.108	3.45E-01	-0.491	1.55E-03	-0.604	1.55E-03
praD	CtesDRAFT_PD4982	OCA decarboxylase	-0.049	5.97E-01	0.132	1.69E-01	-0.292	1.69E-01
praE	CtesDRAFT_PD3647	HPD hydratase	-0.466	5.45E-02	0.34	1.04E-01	0.155	1.04E-01
praF	CtesDRAFT_PD3649	HOV aldolase	0.038	7.84E-01	0.352	1.86E-02	0.258	1.86E-02

praG	CtesDRAFT_PD3648	acetaldehyde dehydrogenase	-0.314	1.28E-02	0.339	4.54E-03	0.129	4.54E-03

Supplementary Table 5 | Changes in protein abundance for the enzymes in the three putative protocatechuate cleavage pathways. Values represent fold changes (FC) in protein abundance for *C. testosteroni* KF-1 cells grown on aromatic compounds relative to cells grown on succinate. Grey boxes are for genes that were not found in the *Comamonas* genome. Statistically significant differential protein abundance was determined by calculating a *Z*-score for protein abundance differences, assuming a standard normal distribution, and translating *Z*-score values to *P*-values. These *P*-values were further corrected using the *q*-value method (controlled to 0.05) to correct for multiple testing familywise error rate. ND, protein not detected. Present, protein found during growth on the aromatic compound but not succinate.

Gene Names	Locus Tag	Protein	Protein abundance Log_2(FC)					
			(4HB/SUCC)		(VAN/SUCC)		(TER/SUCC)	
			Average	P-values	Average	P-values	Average	P-values
PmdA	CtesDRAFT_PD1897	PCA 4,5-dioxygenase, alpha subunit	Present		Present		Present	
PmdB	CtesDRAFT_PD1898	PCA 4,5-dioxygenase, beta subunit	4.685	1.47E-06	6.066	3.43E-04	5.042	3.43E-04
PmdC	CtesDRAFT_PD1899	CHMS dehydrogenase	4.281	5.33E-19	5.771	4.70E-09	4.502	4.70E-09
PmdD	CtesDRAFT_PD1896	PDC hydrolase	4.202	9.5E-27	5.221	2.99E-18	4.759	2.99E-18
PmdU	CtesDRAFT_PD1892	OMA tautomerase	Present		Present		Present	
PmdE	CtesDRAFT_PD1894	OMA hydratase	4.47	7.75E-17	5.91	3.62E-14	5.183	3.62E-14
PmdF	CtesDRAFT_PD1895	CHA aldolase	Present		Present		Present	
PcaH	CtesDRAFT_PD0424	PCA 3,4-dioxygenase, beta subunit	ND		ND		ND	
PcaG	NF	PCA 3,4-dioxygenase alpha subunit						
PcaB	CtesDRAFT_PD5238	3-carboxy-cis,cis-muconate cycloisomerase	ND		ND		ND	
PcaC	CtesDRAFT_PD5237	CML decarboxylase	ND		ND		ND	
PcaD	CtesDRAFT_PD4384	βKAP enol-lactonase	ND		ND		ND	
PcaI	CtesDRAFT_PD5468	3-oxoadipyl-CoA-transferase	ND		ND		ND	
PcaJ	CtesDRAFT_PD1882	3-oxoadipyl-CoA-transferase	ND		ND		ND	
PcaF	CtesDRAFT_PD4382	3-oxoadipyl-CoA thiolase	ND		ND		ND	
PraA	NF	PCA 2,3-dioxygenase						
PraH	NF	5CHMS decarboxylase						
PraB	CtesDRAFT_PD5289	HMS dehydrogenase	ND		ND		ND	
PraC	CtesDRAFT_PD5334	OCA tautomerase	ND		ND		ND	
PraD	CtesDRAFT_PD4982	OCA decarboxylase	ND		ND		ND	
PraE	CtesDRAFT_PD3647	HPD hydratase	ND		ND		ND	
PraF	CtesDRAFT_PD3649	HOV aldolase	ND		ND		ND	

PraG	CtesDRAFT_PD3648	acetaldehyde dehydrogenase	ND	ND	ND

Supplementary Table 6 | Protein sequence identity of ortho cleavage pathway enzymes annotated in the *C. testosteroni* and *P. putida* genomes. Abbreviations for metabolites are described in Fig. 1 of the main text. NF, not found; NA, not applicable.

C. testosteroni Kf-1	Comparison organism				
Tag	Names	Function	Organism	Identity (%)	UniProt Accession No.
CtesDRAFT_PD0424	PcaH	PCA 3,4-dioxygenase, beta subunit	*P. putida* KT2440	30.4%	Q88E12
NF	PcaG	PCA 3,4-dioxygenase alpha subunit	*P. putida* KT2440	NA	Q88E13
CtesDRAFT_PD5238	PcaB	3-carboxy-cis,cis-muconate cycloisomerase	*P. putida* KT2440	38.6%	Q88N37
CtesDRAFT_PD5237	PcaC	CML decarboxylase	*P. putida* KT2440	43.2%	Q88N35
CtesDRAFT_PD4384	PcaD	βKAP enol-lactonase	*P. putida* KT2440	49.6%	Q88N36
CtesDRAFT_PD5468	PcaI	3-oxoadipyl-CoA-transferase	*P. putida* KT2440	66.4%	Q88FX5
CtesDRAFT_PD1882	PcaJ	3-oxoadipyl-CoA-transferase	*P. putida* KT2440	58.0%	P0A101
CtesDRAFT_PD4382	PcaF	3-oxoadipyl-CoA thiolase	*P. putida* KT2440	68.3%	Q88N39

Supplementary Table 7 | Protein sequence identity of 2,3-meta cleavage pathway enzymes annotated in the *C. testosteroni* and *Paenibacillus* sp. JJ-1b genomes. Abbreviations for metabolites are described in Fig. 1 of the main text. NF, not found; NA, not applicable.

C. testosteroni Kf-1	Comparison organism				
Tag	Names	Function	Organism	Identity (%)	UniProt Accession No.
NF	PraA	PCA 2,3-dioxygenase	*Paenibacillus* sp. JJ-1b	NA	C4TP01
NF	PraH	5CHMS decarboxylase	*Paenibacillus* sp. JJ-1b	NA	C4TP08
CtesDRAFT_PD5289	PraB	HMS dehydrogenase	*Paenibacillus* sp. JJ-1b	41.2%	C4TP02

CtesDRAFT_PD5334	PraC	OCA tautomerase	*Paenibacillus* sp. JJ-1b	29.5%	C4TP07
CtesDRAFT_PD4982	PraD	OCA decarboxylase	*Paenibacillus* sp. JJ-1b	23.3%	C4TP06
CtesDRAFT_PD3647	PraE	HPD hydratase	*Paenibacillus* sp. JJ-1b	46.3%	C4TP03
CtesDRAFT_PD3649	PraF	HOV aldolase	*Paenibacillus* sp. JJ-1b	57.6%	C4TP05
CtesDRAFT_PD3648	PraG	acetaldehyde dehydrogenase	*Paenibacillus* sp. JJ-1b	56.7%	C4TP04

Supplementary Table 8 | Protein sequence identity of 4,5-meta cleavage pathway enzymes annotated in the *C. testosteroni* and two previously characterized species. Abbreviations for metabolites are described in Fig. 1 of main text.

C. testosteroni Kf-1	Comparison organisms				
Tag	Names	Function	Organism	Identity (%)	UniProt Accession No.
CtesDRAFT_PD1897	PmdA	PCA 4,5-dioxygenase, alpha subunit	*Comamonas* sp. E6	96.7%	Q8RNY0
	LigA	PCA 4,5-dioxygenase alpha chain	Sphingobium sp. SYK-6	65.0%	P22635
CtesDRAFT_PD1898	PmdB	PCA 4,5-dioxygenase, beta subunit	*Comamonas* sp. E6	99.7%	D1MWA0
	LigB	PCA 4,5-dioxygenase, beta chain	*Sphingobium* sp. SYK-6	60.5%	P22636
CtesDRAFT_PD1899	PmdC	CHMS dehydrogenase	*Comamonas* sp. E6	99.1%	D1MWA1
	LigC	CHMS dehydrogenase	Sphingobium sp. SYK-6	76.6%	Q9KWL3
CtesDRAFT_PD1896	PmdD	PDC hydrolase	*Comamonas* sp. E6	98.0%	D1MW98
	LigI	PDC hydrolase	Sphingobium sp. SYK-6	54.6%	O87170
CtesDRAFT_PD1892	PmdU	OMA tautomerase	*Comamonas* sp. E6	95.7%	D1MW94
	LigU	OMA isomerase	Sphingobium sp. SYK-6	No significant similarity	Q0KJL4
CtesDRAFT_PD1894	PmdE	OMA hydratase	*Comamonas* sp. E6	100.0%	D1MW96
	LigJ	OMA hydratase	Sphingobium sp. SYK-6	63.50%	G2IQQ5

CtesDRAFT_PD1895	PmdF	CHA aldolase	*Comamonas* sp. E6	98.7%	D1MW97
	LigK	CHA aldolase	Sphingobium sp. SYK-6	65.90%	G2IQQ8

Supplementary Table 9 | Sequence similarity between the putative 4-hydroxybenzoate transporter.

C. testosteroni Kf-1	Comparison organisms				
Tag	Names	Function	Organism	Identity (%)	UniProt Accession No.
CtesDRAFT_PD1893	PmdK	4-hydroxybenzoate transporter	*Comamonas* sp. E6	98.5%	D1MW95
	PcaK	4-hydroxybenzoate transporter	*Pseudomonas putida* KT2440	49.1%	Q88N40

Supplementary Table 10 | Differential gene expression in central carbon metabolism. Values represent fold changes (FC) in transcript abundance for *C. testosteroni* KF-1 cells grown on aromatic compounds relative to cells grown on succinate. Statistically significant differential transcript abundance was determined using Degust software package [53, main text].

Gene Names or identifier	Locus Tag	Protein	Transcript abundance Log_2(FC) (4HB/SUCC)		(VAN/SUCC)		(TER/SUCC)	
			Average	P-values	Average	P-values	Average	P-values
PDH	CtesDRAFT_PD2531	Pyruvate dehydrogenase	0.681	3.70E-07	0.351	2.31E-04	0.443	2.62E-05
GltA	CtesDRAFT_PD1274	Citrate synthase	0.426	1.76E-07	0.119	0.011	0.589	4.35E-09
AcnB	CtesDRAFT_PD1285	Aconitate hydratase B (aconitase)	0.430	2.37E-06	1.026	1.46E-10	0.245	4.48E-04
AcnA	CtesDRAFT_PD1305	Aconitate hydratase B (aconitase)	0.424	3.14E-04	0.537	3.81E-05	0.052	0.546
IDH1	CtesDRAFT_PD1820	Isocitrate dehydrogenase [NADP]	0.380	2.77E-05	0.295	2.72E-04	0.866	4.19E-09
IDH2	CtesDRAFT_PD1824	Isocitrate dehydrogenase [NADP]	-0.410	4.23E-05	-0.090	0.196	0.526	3.71E-06
Suc1	CtesDRAFT_PD2650	2-oxoglutarate dehydrogenase	0.215	0.004	0.707	6.01E-08	0.739	3.42E-08
Suc2	CtesDRAFT_PD2649	2-oxoglutarate dehydrogenase	0.204	0.004	0.546	6.80E-07	0.669	7.10E-08
SucA	CtesDRAFT_PD2651	2-oxoglutarate dehydrogenase	0.229	0.013	0.625	4.16E-06	0.721	8.87E-07
SucD	CtesDRAFT_PD5114	Succinyl-coenzymeA synthase	-0.361	1.71E-04	-1.034	2.39E-09	0.122	0.096
SucC	CtesDRAFT_PD5113	Succinyl-coenzymeA synthase	-0.374	6.21E-05	-1.120	3.76E-10	0.084	0.207

SdhB	CtesDRAFT_PD1276	Succinate dehydrogenase	0.225	1.79E-04	0.051	0.253	0.426	3.09E-07
SdhA	CtesDRAFT_PD1279	Succinate dehydrogenase	0.180	0.006	-0.011	0.837	0.417	5.33E-06
SdhC	CtesDRAFT_PD1277	Succinate dehydrogenase	0.240	1.29E-04	-0.021	0.629	0.406	6.86E-07
SdhD	CtesDRAFT_PD1278	Succinate dehydrogenase	0.212	0.001	0.052	0.272	0.387	1.90E-06
FumC	CtesDRAFT_PD3618	Fumarate hydratase	0.399	9.53E-05	0.004	0.960	-0.453	4.34E-05
FH2	CtesDRAFT_PD3620	Fumarate hydratase	0.078	0.410	-0.505	1.17E-04	0.696	6.43E-06
Mdh	CtesDRAFT_PD1281	Malate dehydrogenase	0.547	4.47E-07	0.064	0.271	0.918	1.32E-09
Mqo	CtesDRAFT_PD1015	malate:quinone oxidoreductase	-2.444	3.80E-15	-5.632	2.81E-17	-5.378	5.63E-17
AceA	CtesDRAFT_PD1530	Isocitrate lyase	-0.110	0.378	0.253	0.049	0.441	0.003
MS	CtesDRAFT_PD5335	Malate synthase	0.578	0.002	-0.748	2.28E-04	-0.462	0.008
PycB	CtesDRAFT_PD0191	Pyruvate carboxylase	0.029	0.724	0.617	2.93E-06	0.104	0.232
Pck	CtesDRAFT_PD5031	PEP carboxykinase	-1.315	5.07E-10	0.531	1.58E-05	-0.130	0.111
Ppc	CtesDRAFT_PD1089	PEP carboxylase	-0.413	2.60E-06	-0.517	2.40E-07	-0.110	0.047
ME	CtesDRAFT_PD0934	Malic enzyme	-0.209	0.001	-0.625	2.33E-08	-0.151	0.010
MaeB	CtesDRAFT_PD5092	Malic enzyme	-1.206	1.76E-11	-0.280	1.59E-04	-0.360	1.42E-05
PEPs	CtesDRAFT_PD3828	Phosphoenolpyruvate synthase	-0.445	1.33E-04	-0.082	0.335	0.772	6.94E-07
PK	CtesDRAFT_PD5271	Pyruvate kinase	0.271	2.27E-05	-0.395	4.94E-07	0.269	2.49E-05
Eno	CtesDRAFT_PD3884	Enolase	-0.233	0.014	-0.355	0.001	0.485	6.71E-05
Gpm	CtesDRAFT_PD0700	2,3-bisphosphoglycerate-dependent phosphoglycerate mutase	-0.185	0.014	-0.790	3.25E-08	-0.054	0.422
Pgk	CtesDRAFT_PD5167	Phosphoglycerate kinase	-0.645	4.46E-06	-0.329	0.002	-0.121	0.169
Gap	CtesDRAFT_PD5265	Glyceraldehyde-3-phosphate dehydrogenase	-0.451	5.70E-05	-0.030	0.697	0.617	3.02E-06
TpiA	CtesDRAFT_PD3953	Triosephosphate isomerase	-0.206	0.027	-0.375	0.001	0.183	0.045
Fba	CtesDRAFT_PD5277	Fructose-1,6-bisphosphate aldolase	-0.549	4.17E-07	0.023	0.692	0.215	0.002
Fbp	CtesDRAFT_PD1620	Fructose-1,6-bisphosphatase	-0.086	0.203	-0.321	2.78E-04	-0.002	0.978
Pgi1	CtesDRAFT_PD0867	Glucose-6-phosphate isomerase	-0.113	0.227	0.538	2.62E-05	-0.155	0.126
Rpe	CtesDRAFT_PD5427	Ribulose-phosphate 3-epimerase	0.050	0.278	-0.110	0.030	-0.028	0.541
RpiA	CtesDRAFT_PD2537	Ribose-5-phosphate isomerase A	0.030	0.624	-0.301	2.89E-04	0.111	0.087

Tkt	CtesDRAFT_PD5266	transketolase	-0.191	0.003	-0.464	1.02E-06	0.099	0.079
Tal	CtesDRAFT_PD0868	Transaldolase	-0.263	0.381	0.463	0.102	-0.097	0.750
PntA	CtesDRAFT_PD4838	NAD(P) transhydrogenase subunit alpha	-0.142	0.029	0.169	0.005	0.091	0.091
PntB	CtesDRAFT_PD4837	NAD(P) transhydrogenase subunit beta	-0.207	0.012	0.223	0.006	0.183	0.021

Supplementary Table 11 | Changes in protein abundance for the enzymes in central carbon metabolism. Values represent fold changes (FC) in protein abundance for *C. testosteroni* KF-1 cells grown on aromatic compounds relative to cells grown on succinate. Statistically significant differential protein abundance was determined by calculating a *Z*-score for protein abundance differences, assuming a standard normal distribution, and translating *Z*-score values to *P*-values. These *P*-values were further corrected using the *q*-value method (controlled to 0.05) to correct for multiple testing familywise error rate. ND, protein not detected.

Gene Names or identifier	Locus Tag	Protein	Protein abundance Log_2(FC)					
			(4HB/SUCC)		(VAN/SUCC)		(TER/SUCC)	
			Average	P-values	Average	P-values	Average	P-values
PDH	CtesDRAFT_PD2531	Pyruvate dehydrogenase	0.174	0.286	-0.224	0.371	0.024	0.397
GltA	CtesDRAFT_PD1274	Citrate synthase	0.456	0.229	0.405	0.320	0.270	0.336
AcnB	CtesDRAFT_PD1285	Aconitate hydratase B (aconitase)	0.221	0.177	-0.189	0.370	0.419	0.027
AcnA	CtesDRAFT_PD1305	Aconitate hydratase B (aconitase)	1.205	0.000	0.819	0.351	0.361	0.162
IDH1	CtesDRAFT_PD1820	Isocitrate dehydrogenase [NADP]	0.463	0.139	0.573	0.229	0.523	0.094
IDH2	CtesDRAFT_PD1824	Isocitrate dehydrogenase [NADP]	-0.223	0.333	ND		0.416	0.275
Suc1	CtesDRAFT_PD2650	2-oxoglutarate dehydrogenase	0.158	0.314	0.574	0.272	0.402	0.124
Suc2	CtesDRAFT_PD2649	2-oxoglutarate dehydrogenase	0.309	0.206	0.963	0.064	0.179	0.335
SucA	CtesDRAFT_PD2651	2-oxoglutarate dehydrogenase	0.182	0.347	0.911	0.097	0.259	0.304
SucD	CtesDRAFT_PD5114	Succinyl-coenzymeA synthase	0.066	0.391	-0.326	0.372	0.115	0.377
SucC	CtesDRAFT_PD5113	Succinyl-coenzymeA synthase	0.024	0.397	-1.268	0.018	0.052	0.393
SdhB	CtesDRAFT_PD1276	Succinate dehydrogenase	0.155	0.387	-1.720	0.160	-0.068	0.397
SdhA	CtesDRAFT_PD1279	Succinate dehydrogenase	0.277	0.368	ND		0.082	0.397
SdhC	CtesDRAFT_PD1277	Succinate dehydrogenase	0.133	0.374	-0.514	0.260	-0.100	0.384
SdhD	CtesDRAFT_PD1278	Succinate dehydrogenase	-0.081	0.387	ND		ND	

FumC	CtesDRAFT_PD3618	Fumarate hydratase	1.374	0.027	0.469	0.264	0.011	0.399
FH2	CtesDRAFT_PD3620	Fumarate hydratase	-0.111	0.383	-0.419	0.384	0.032	0.398
Mdh	CtesDRAFT_PD1281	Malate dehydrogenase	0.645	0.054	0.319	0.366	0.489	0.188
Mqo	CtesDRAFT_PD1015	malate:quinone oxidoreductase	ND		ND		ND	
AceA	CtesDRAFT_PD1530	Isocitrate lyase	ND		ND		ND	
MS	CtesDRAFT_PD5335	Malate synthase	0.832	0.210	0.286	0.384	0.016	0.399
PycB	CtesDRAFT_PD0191	Pyruvate carboxylase	ND		ND		ND	
Pck	CtesDRAFT_PD5031	PEP carboxykinase	-1.299	0.005	1.123	0.174	-0.519	0.160
Ppc	CtesDRAFT_PD1089	PEP carboxylase	0.222	0.308	ND		-0.100	0.379
ME	CtesDRAFT_PD0934	Malic enzyme	-0.050	0.397	-0.161	0.393	-0.343	0.315
MaeB	CtesDRAFT_PD5092	Malic enzyme	ND		-0.106	0.397	-0.135	0.391
PEPs	CtesDRAFT_PD3828	PEP synthase	-0.630	0.024	-0.536	0.179	0.016	0.398
PK	CtesDRAFT_PD5271	Pyruvate kinase	0.341	0.183	0.223	0.305	0.351	0.267
Eno	CtesDRAFT_PD3884	Enolase	-0.146	0.379	-0.049	0.398	0.333	0.274
Gpm	CtesDRAFT_PD0700	2,3-bisphosphoglycerate-dependent phosphoglycerate mutase	-0.304	0.346	-0.479	0.272	-0.160	0.386
Pgk	CtesDRAFT_PD5167	Phosphoglycerate kinase	-0.595	0.197	0.120	0.385	-0.360	0.315
Gap	CtesDRAFT_PD5265	Glyceraldehyde-3-phosphate dehydrogenase	-0.452	0.169	-0.861	0.215	0.109	0.383
TpiA	CtesDRAFT_PD3953	Triosephosphate isomerase	-0.332	0.281	0.443	0.338	-0.162	0.374
Fba	CtesDRAFT_PD5277	Fructose-1,6-bisphosphate aldolase	-0.398	0.107	-0.603	0.145	-0.176	0.316
Fbp	CtesDRAFT_PD1620	Fructose-1,6-bisphosphatase	0.077	0.386	-0.357	0.192	-0.072	0.394
Pgi1	CtesDRAFT_PD0867	Glucose-6-phosphate isomerase	ND		ND		ND	
Rpe	CtesDRAFT_PD5427	Ribulose-phosphate 3-epimerase	ND		ND		ND	
RpiA	CtesDRAFT_PD2537	Ribose-5-phosphate isomerase A	-0.025	0.398	-0.199	0.335	-1.158	0.186
Tkt	CtesDRAFT_PD5266	transketolase	-0.133	0.388	0.105	0.395	-0.099	0.393

Tal	CtesDRAFT_PD0868	Transaldolase	0.252	0.387	0.806	0.283	-0.263	0.388
PntA	CtesDRAFT_PD4838	NAD(P) transhydrogenase subunit alpha	ND		ND		ND	
PntB	CtesDRAFT_PD4837	NAD(P) transhydrogenase subunit beta	ND		ND		ND	

Supplementary Table 12 | Free energy calculated from the ^{13}C-metabolic flux analysis. The 95% confidence intervals of the ∆G are reported for the lower bound (L.B) and upper bound (U.B.)

Reaction	Flux ratio	$\Delta G = -RT \ln(J^+/J^-)$		
Forward flux direction	J^+/J^-	*∆G (kJ/mol)*	*L.B.*	*U.B.*
Succinate → Fumarate	1.03	-0.078	0	-0.029
Fumarate → Malate	1.00	-0.010	-0.046	-0.0056
Malate → OAA	1.04	-0.10	-0.10	-0.099
Malate → Pyruvate	4.63	-3.95	-5.67	0.10

Supplementary Table 13. Fragmentation analysis of singly labeled citrate and relative intensity (Rel. Int.) of product ions.

	M+1 Citrate (192.0219 m/z)			
	m+0		m+1	
Carbon origin	m/z found	Rel. int.	m/z found	Rel. int.
[3,2,1,5]	85.02757	0.32 ± 0.06	86.03116	0.68 ± 0.06
[3,2,1] or [1,2,5]	87.00713	0.31 ± 0.01	88.01083	0.69 ± 0.01

Supplementary Table 14 | Intracellular metabolic flux rates. Rates were determined from parallel quantitative flux modeling of *C. testosteroni* KF-1 during growth on [1-^{13}C]-(carboxyl)-4HB and [$^{13}C_6$]-(phenyl)-4HB. For reversible reactions, the net flux is in the direction shown. Cellular secretions are designated as EX and biomass efflux is designated as B. Error is shown as lower bound (LB) and upper bound (UB) 95% confidence intervals and standard deviation (SD) for three biological replicates (n=3). Refer to the legend of Fig. 2 for metabolite names.

Uptake rate (mmol/g_{cdw}/h):11.6 ± 3.4		Uptake (%)			
Rxn	Flux	Best fit	LB (95%)	UB (95%)	SD
v1	4HB_EX = 4HB	100.0	98.3	101.4	0.8
v2	4HB = PCA	100.0	98.3	101.4	0.8
v3	PCA = PDC	95.7	93.9	97.3	0.9
v4	PDC = PYR + OAA	91.4	88.6	94.0	1.4
v5	SUCC = FUM	79.8	75.1	85.1	2.5
v6	FUM = 0.5 MAL + 0.5 MAL	79.8	75.1	85.1	2.5
v7	MAL = OAA	9.5	2.8	14.8	3.0
v8	ACCOA + OAA = CIT	88.7	83.8	94.4	2.6
v9	CIT = AKG + CO2	85.4	78.3	93.9	3.9
v10	AKG = 0.5 SUCC + 0.5 SUCC + CO2	76.4	69.3	84.7	3.9
v11	CIT = 0.5 SUCC + 0.5 SUCC + GLOX	3.4	0.0	7.9	2.0
v12	GLOX + ACCOA = MAL	3.4	0.0	7.9	2.0
v13	OAA = PEP + CO2	5.7	4.4	9.2	1.2
v14	PEP + CO2 = OAA	0.0	0.0	5.1	1.3
v15	OAA = PYR + CO2	0.0	0.0	3.5	0.9
v16	PYR + CO2 = OAA	0.0	0.0	5.6	1.4
v17	MAL = PYR + CO2	73.3	67.0	80.2	3.3
v18	PYR = ACCOA + CO2	129.3	120.2	138.7	4.6
v19	PYR = PEP	17.4	13.8	30.4	4.2
v20	PEP = PYR	0.0	0.0	15.5	3.9
v21	PEP = 3PG	17.6	13.6	21.6	2.0
v22	3PG = GAP	11.1	8.2	14.0	1.4
v23	GAP = DHAP	4.6	1.2	3.4	0.6
v24	DHAP + GAP = FBP	3.9	2.9	5.0	0.5
v25	FBP = F6P	3.9	2.9	5.0	0.5
v26	F6P = G6P	1.4	0.8	2.1	0.3
v27	S7P + GAP = E4P + F6P	0.1	-0.4	0.5	0.2
v28	R5P + Xu5P = S7P + GAP	0.1	-0.4	0.5	0.2
v29	E4P + Xu5P = F6P + GAP	-2.6	-3.4	-2.0	0.3
v30	Xu5P = R5P	2.5	1.7	3.4	0.4
v31	CO2 = CO2_EX	370.2	351.5	515.4	41.0
v32	CO2_EX = CO2	0.0	0.0	2.5	0.6
v33	AKG = AKG_EX	4.0	2.9	5.0	0.5
v34	PCA = PCA_EX	4.3	3.5	5.0	0.4
v35	PDC = PDC_EX	4.3	2.3	6.2	1.0
v36	MAL = MAL_EX	0.2	0.1	0.3	0.1
v37	PYR = PYR_EX	5.5	4.0	7.2	0.8
v38	3PG = B_3PG	6.5	3.7	9.3	1.4
v39	AKG = B_AKG	5.0	2.9	7.0	1.0
v40	DHAP = B_DHAP	0.6	0.4	0.9	0.1

v41	E4P = B_E4P	2.7	1.9	3.6	0.4
v42	G6P = B_G6P	1.4	0.8	2.1	0.3
v43	OAA = B_OAA	6.6	3.7	9.5	1.5
v44	PEP = B_PEP	5.5	3.1	7.4	1.1
v45	PYR = B_PYR	12.6	7.9	17.2	2.3
v46	R5P = B_R5P	2.4	1.4	3.7	0.6
v47	ACCOA = B_ACCOA	37.3	29.6	43.8	3.6

Supplementary Table 15 | *In vitro* calculation of cofactor specificities for ME, MaeB, IDH2, IDH1, Mdh, and PobA from *C. testosteroni* KF-1. Specific activities (mmol min^{-1} g^{-1}) are reported for each replicate (listed as 1, 2, and 3) as well as the relative activity toward a cofactor as a percentage of the total measured activity.

MALIC ENZYME - CtesDRAFT_PD0933 (ME)					
	1	2	3	Avg	SD
$NADP^+$ (mmol min^{-1} g^{-1})	0.81	0.81	0.83	0.82	0.01
NAD^+ (mmol min^{-1} g^{-1})	0	0	0	0	0
$NADP^+$ (%)	100	100	100	100	0
NAD^+ (%)	0	0	0	0	0
MALIC ENZYME - CtesDRAFT_PD5092 (MaeB)					
	1	2	3	Avg	SD
$NADP^+$ (mmol min^{-1} g^{-1})	0.016	0.016	0.015	0.016	0.001
NAD^+ (mmol min^{-1} g^{-1})	0.099	0.096	0.100	0.098	0.002
$NADP^+$ (%)	14.1	14.3	12.8	13.7	0.8
NAD^+ (%)	85.9	85.7	87.2	86.3	0.8
ISOCITRATE DEHYDROGENASE - CtesDRAFT_PD1824 (IDH2)					
	1	2	3	Avg	SD
$NADP^+$ (mmol min^{-1} g^{-1})	12.12	11.50	13.35	12.32	0.94
NAD^+ (mmol min^{-1} g^{-1})	0.11	0.10	0.11	0.10	0.00
$NADP^+$ (%)	99.1	99.1	99.2	99.2	0.0
NAD^+ (%)	0.9	0.9	0.8	0.8	0.0
ISOCITRATE DEHYDROGENASE - CtesDRAFT_PD1820 (IDH1)					
	1	2	3	Avg	SD

$NADP^+$ (mmol min^{-1} g^{-1})	61.55	60.39	61.69	61.21	0.71
NAD^+ (mmol min^{-1} g^{-1})	0.00	0.00	0.00	0.00	0.00
$NADP^+$ (%)	100	100	100	100	0
NAD^+ (%)	0.0	0.0	0.0	0.0	0.0
MALATE DEHYDROGENASE - CtesDRAFT_PD1281 (Mdh)					
	1	2	3	Avg	SD
$NADP^+$ (mmol min^{-1} g^{-1})	30.66	30.82	31.26	30.91	0.31
NAD^+ (mmol min^{-1} g^{-1})	319.47	319.47	319.47	319.47	0.00
$NADP^+$ (%)	8.8	8.8	8.9	8.8	0.1
NAD^+ (%)	91.2	91.2	91.1	91.2	0.1
4HB 3-MONOOXYGENASE - CtesDRAFT_PD2627 (PobA)					
	1	2	3	Avg	SD
$NADP^+$ (mmol min^{-1} g^{-1})	1.64	1.65	1.78	1.69	0.08
NAD^+ (mmol min^{-1} g^{-1})	0.00	0.00	0.00	0.00	0.00
$NADP^+$ (%)	100	100	100	100	0
NAD^+ (%)	0	0	0	0	0

Supplementary Table 16 | Protein sequence identity comparison of PobA. CtesDRAFT_PD2627 (PobA) in *Comamonas testosteroni* KF-1 was compared to PobA in species with previously characterized cofactor preference [30, main text]

Species	Cofactor Preference	Identity (%)	Accession number
Acinetobacter sp. ADP1	NADPH	70.95	WP_004926674.1
Cupriavidus necator JMP134 (synthetic)	NADPH	69.92	AOR50759.1
Pseudomonas aeruginosa PAO1	NADPH	67.87	WP_003112685.1
Rhizobium leguminosarum B155	NADPH	65.64	AAA73519
Pseudomonas putida KT2440	NADPH	66.24	WP_010954394.1
Azotobacter chroococcum ATCC9043	NADPH	65.04	AAB70835

Cupriavidus necator JMP134 (synthetic)	NAD(P)H	53.08	AOR50758.1
Pseudomonas sp. CBS3	NAD(P)H	52.59	CAA52824.1
Rhodococcus opacus 557	NADH	49.49	ANS30736
Rhodococcus rhodnii 135	NADH	48.59	KF234627
Corynebacterium glutamicum ATCC 13032	NAD(P)H	40.57	WP_011014104.1

Supplementary Table 17 | Quantitative determination of NADH/FADH2, NADPH, and ATP production and consumption. Absolute rates (mmol g_{CDW}^{-1} h^{-1}) were determined from cellular fluxes (Table S10) and species-specific biomass stoichiometry in 4-hydroxybenzoate-grown cells of *C. testosteroni* KF-1.

CO-FACTOR BALANCES (mmol/gCDW/h)			
NADH/FADH2		Avg	SD
	TCA Cycle	34.2	0.64
	EMP pathway	-1.3	0.17
	Anabolism	0.7	0.03
	Transhydrogenase	6.5	0.76
	Oxidative Phosphorylation	-40.1	1.00
NADPH			
	TCA Cycle	9.9	0.45
	Malic Enzyme	8.5	0.38
	4,5-meta	11.1	0.09
	4HB uptake	-11.6	0.09
	Anabolism	-11.4	0.45
	Transhydrogenase	-6.5	0.76
ATP			
	TCA Cycle	8.9	0.4
	Pyruvate Kinase	0.0	0.4
	Oxidative Phosphorylation	60.2	1.5
	Anabolism	-20.9	0.8

Supplementary Table 18 | Comparison of transcriptomics, proteomics, and fluxomics. Fold changes (Log_2) of gene transcript levels (transcriptomics), protein abundance (proteomics), and metabolic fluxes (fluxomics) in *C. testosteroni* KF-1 cells grown on 4-hydroxybenzoate relative to cells grown on succinate. Refer to the legend in Fig. 1 for metabolite abbreviations. ND, not detected.

Reactions	Proteins	ORF number	Transcriptomics	Proteomics	Fluxomics
Pyruvate → Acetyl-CoA	PDH	CtesDRAFT_PD2531	0.681	0.174	1.107
Acetyl-CoA + OAA -> Citrate	GltA	CtesDRAFT_PD1274	0.426	0.456	1.53
Citrate -> Isocitrate	AcnB	CtesDRAFT_PD1285	0.43	0.221	1.474
Citrate -> Isocitrate	AcnA	CtesDRAFT_PD1305	0.424	1.205	1.474
isocitrate -> α-ketoglutarate	IDH1	CtesDRAFT_PD1820	0.38	0.463	1.474
isocitrate -> α-ketoglutarate	IDH2	CtesDRAFT_PD1824	-0.41	-0.223	1.474
α-Ketoglutarate -> Succinyl-CoA	Suc	CtesDRAFT_PD2650	0.215	0.158	1.541
α-Ketoglutarate -> Succinyl-CoA	Suc	CtesDRAFT_PD2649	0.204	0.309	1.541
α-Ketoglutarate -> Succinyl-CoA	SucA	CtesDRAFT_PD2651	0.229	0.182	1.541
Succinyl-CoA -> Succinate	SucD	CtesDRAFT_PD5114	-0.361	0.066	1.541
Succinyl-CoA -> Succinate	SucC	CtesDRAFT_PD5113	-0.374	0.024	1.541
Succinate <-> Fumarate	SdhB	CtesDRAFT_PD1276	0.225	0.155	-0.664
Succinate <-> Fumarate	SdhA	CtesDRAFT_PD1279	0.18	0.277	-0.664
Succinate <-> Fumarate	SdhC	CtesDRAFT_PD1277	0.24	0.133	-0.664
Succinate <-> Fumarate	SdhD	CtesDRAFT_PD1278	0.212	-0.081	-0.664
Fumarate <-> malate	FumC	CtesDRAFT_PD3618	0.399	1.374	-0.664
Fumarate <-> malate	FH2	CtesDRAFT_PD3620	0.078	-0.111	-0.664
Malate <-> OAA	Mdh	CtesDRAFT_PD1281	0.547	0.645	-3.645
Malate = OAA	Mqo	CtesDRAFT_PD1015	-2.444	ND	-3.645
Isocitrate -> Succinate + Glyoxylate	AceA	CtesDRAFT_PD1530	-0.11	ND	ND
Acetyl-CoA + Glyoxylate -> Malate	MS	CtesDRAFT_PD5335	0.578	0.832	ND
Pyruvate -> OAA	PycB	CtesDRAFT_PD0191	0.029	ND	ND
OAA -> PEP	Pck	CtesDRAFT_PD5031	-1.315	-1.299	-3.204
PEP -> OAA	Ppc	CtesDRAFT_PD1089	-0.413	0.222	ND

Malate <-> Pyruvate	ME	CtesDRAFT_PD0934	-0.209	-0.05	3.369
Malate <-> Pyruvate	MaeB	CtesDRAFT_PD5092	-1.206	ND	3.369
Pyruvate -> PEP	PEPs	CtesDRAFT_PD3828	-0.445	-0.63	ND
PEP -> Pyruvate	PK	CtesDRAFT_PD5271	0.271	0.341	ND
PEP <-> 2PG	Eno	CtesDRAFT_PD3884	-0.233	-0.146	0.214
2PG <-> 3PG	Gpm	CtesDRAFT_PD0700	-0.185	-0.304	0.214
3PG <-> 1,3-BPG	Pgk	CtesDRAFT_PD5167	-0.645	-0.595	0.232
1,3-BPG -> GAP	Gap	CtesDRAFT_PD5265	-0.451	-0.452	0.232
GAP <-> DHAP	TpiA	CtesDRAFT_PD3953	-0.206	-0.332	0.171
FBP <-> GAP + DHAP	Fba	CtesDRAFT_PD5277	-0.549	-0.398	0.16
FBP -> F6P	Fbp	CtesDRAFT_PD1620	-0.086	0.077	0.16

Supplementary Table 19 | The Log_2 fold change in the ratio of substrate (S) to product (P) of quantified intracellular metabolites and ^{13}C-metabolic fluxes. Cells were grown on 4-hydroxybenzoate (4HB) and compared to growth on succinate (SUCC). The flux ratio for the reaction of pyruvate to PEP could not be calculated because the flux during growth on succinate was zero.

Reaction	S to P ratio	Flux
	Log_2(4HB/SUCC)	Log_2(4HB/SUCC)
Pyruvate → Acetyl-CoA	3.58 ± 0.01	1.11
Malate → Pyruvate	-3.26 ± 2.22	3.37
Succinate → Fumarate	-2.72 ± 0.29	-0.66
Fumarate → Malate	-1.6 ± 0.94	-0.66
Pyruvate → PEP	1.49 ± 0.03	NF
PEP → DHAP	1.39 ± 0.38	0.23
F6P → G6P	-0.06 ± 0.18	0.28

Supplementary Table 20 | Measured allosteric regulation of ME (CtesDRAFT_PD0934) in the presence of five potential effectors. Specific activities (mmol min^{-1} g^{-1}) are reported for each replicate (listed as 1, 2, and 3) as well as the average (Avg) ± standard deviation (SD) of the three technical replicates.

	Aspartate	Glutamate	Succinate	Fumarate	aKG
**0 mM**					
1	0.081				
2	0.080				
3	0.081				
Avg ± SD	0.081 ± 0.001				
**0.5 mM**					
1	0.126	0.097	0.129	0.174	0.080
2	0.121	0.092	0.126	0.168	0.074
3	0.128	0.091	0.126	0.166	0.075
Avg ± SD	0.125 ± 0.004	0.093 ± 0.003	0.127 ± 0.002	0.169 ± 0.004	0.076 ± 0.003
**2 mM**					
1	0.153	0.108	0.164	0.181	0.082
2	0.147	0.106	0.156	0.173	0.074
3	0.149	0.105	0.157	0.172	0.077
Avg ± SD	0.149 ± 0.003	0.107 ± 0.002	0.159 ± 0.004	0.175 ± 0.005	0.078 ± 0.004

Supplementary Table 21 | Measured allosteric regulation of MaeB (CtesDRAFT_PD5092) in the presence of five potential effectors. Specific activities (mmol min^{-1} g^{-1}) are reported for each replicate (listed as 1, 2, and 3) as well as the average (Avg) ± standard deviation (SD) of the three technical replicates.

	Aspartate	Glutamate	Succinate	Fumarate	aKG
**0 mM**					
1	0.0087				
2	0.0085				
3	0.0081				
Avg ± SD	0.0084 ± 0.0003				
**0.5 mM**					
1	0.054	0.0112	0.0091	0.056	0.009
2	0.052	0.0106	0.0084	0.051	0.008
3	0.050	0.0104	0.0082	0.050	0.008

Avg ± SD	0.052 ± 0.002	0.0108 ± 0.0004	0.0086 ± 0.005	0.052 ± 0.003	0.008 ± 0.001
**2 mM**					
1	0.0971	0.0129	0.0108	0.092	0.0065
2	0.0902	0.0126	0.0102	0.081	0.0058
3	0.0875	0.0123	0.0097	0.077	0.0057
Avg ± SD	0.092 ± 0.005	0.0126 ± 0.0003	0.0103 ± 0.005	0.083 ± 0.008	0.0060 ± 0.0004

Supplementary Table 22 | Nucleotide sequences of synthetic gene fragments encoding the enzymes studied in this work.

Description	Sequence (5'→3')
Malate dehydrogenase (PD1281) Fragment 1/1	agcaagaagcccgtccgtgtcgccgttaccggcgcagctggccaaatcggttacgccctgttgttccgcatcgcctccggcgaaatgctgggtaaagatcagcccgttattctgcaactgctggaaatccccgacgaaaaggctcaaaacgctctgaagggcgtgatcatggagctggaagactgcgccttccccctgctggccggcatcgaagcccacgccgatcccatgaccgccttcaaggacaccgactacgctctgctggtgggcgcccgtcctcgtggccccggcatggaacgtgctgacctgctggctgccaacgcccagatcttcaccgcccagggcaaggctctgaacgccgtggcttcgcgcaatgtcaaggttctggtggtgggcaaccccgccaacaccaatgcctacatcgccatgaagtcggctcccgatctgcccgccaagaacttcaccgccatgctgcgcctggaccacaaccgtgctgcttcccagctggctgccaagggtggcttcaaggttggcgacatcaggaagctgaccgtgtggggcaaccactcgcccaccatgtacgccgactaccgcttcgccaccgtggacggcaagtccgtgaaggaagccatcaacgaccaggaatggaatgccaacgtgttcctgcccaccgtgggcaagcgtggcgccgccatcatcgctgctcgcggtctgtcgtcggctgcttcggctgccaacgctgccatcgaccacatgcgtgactgggccctgggctccaacggcgaatgggtcaccatgggcgtgccttccaacggcgagtacggcatccccgccggtatcgtgttcggtttccccgtgaccaccacggctgacggcgaatataagattgtcgaaggtctggaaatcgatgccttctcgcaagagtgcatcaacaagactctggctgagctgcaaggcgagcaagacggcgtcaagcacctgctg
Malic enzyme (PD0934) Fragment 1/2	acgcaaaatttaagcagtgcggaacaggcacttcgtgacgctgcccgtgagtaccaccgttcccctgttaagggcaagatttcagtgacgccgacgaaacccttatccaaccagcgtgacctgagcttagcatattcacctggcgtagcctatccctgcttggacatcgaagcggatccgagcactgctgctgaatacacgtctcgcggtaatttggtaggcgtcatcaccaatggtacagcagttttgggactgggcgatatcggtcctttggcgagcaagcctgtcatggaaggtaaggggtgtttgtttaagaagtttgcgggggtcgacgtcttcgatatcgagcttgccgagcgcgaccccgataagcttatcgacattatcgcgtctatggaaccaacacttggaggcatcaacctggaggacatcaaagctcctgagtgtttctacatcgaacaggaactgtcgaagcgtatgaacatccctgtattccatgatgaccagcacgggacagcaatcattagttcggcagcccttctgaatggcctggaacttgtgggtaaggacatcggggcagtcaaggtggcagtatctggcgcaggagccgccgctatcgcgtgtgtagatgtaatggttggacttggaatcaaacgcagcaacgtatatatggtagactcgaagggggtgatctatgagggccgcccaggcgggttagacgcatcaaaacagcgttatgcacaaaatacggaagcccgcactctggcagatgtcgttgacggagccgacgtctttttaggatgctcggcccctggggtacttacggcagacatggtaaagacgatggcggataagcctatcattctggctctggcaaatcctgagcctgaaatccgtcctgaattagcaaaagctattcgtcctgattgcattgttgctacgggtcgctcggactacccgaaccaggtaaacaacgtattatgcttcccctatatctttcgtggtgcgttggactgtggtgctactaagatcacagaggctatgaaattggcttgtgtacgtgagattgccgccctggctaaacaggacgtttcggatgaagttgcggccgcataccaaggtaaagagttgaccttcggaccggattatctgattccgacacctttcgatactcgcttgatcttacgtatcgccccagcagttgca
Malic enzyme (PD0934) Fragment 2/2	caagctgcggccgaatcaggagtagcaacgcgtccaatcgaggacattgccgcgtatcgtgagtcccttacgcgttttgtgtatcaaaccagtatgtttatgcgcccagtgttcgcggcagcaaaggcaaacttacagcgtgttgcgtacgcggaaggtgaagacgaacgcgtattacgtgcggttcaagttgcagtagatgatggtctggcgaagccgatcctgatcggccgtccggctgttatcgaagcccgcattgcaaaggcaggtttgcgcattcagttaggtaaggatgtcgaaatctgtaatcccgaggatgatccacgcttccgtcagtactgggagacgtatcacaaattgatgggacgtaacggagtaactcccgaagctgcgaaggctgcagtgcgtcgttcgaatacattgattgccgctttaatggtccatttgggggatgcggacgctatgctgt

	gcggattagtcggacgttttgactcccacttggcgcaccttgaggacgtattggggttaaaaaagggggcaaacgaat<br>ttgcaactgtaaacgcggtaatgttggaaagcgggaccctgttcgtagctgatacttatatcaatgatgccccttcggcg<br>gaagagttagctgagattgcaaaaatggcggctgacgaagttgcacgctttgggcttcctcccaaagttgcatttctgtc<br>tcattcaaactatggatctagcacgcgcggttcggcccgtaagatgcgtgctgctcgcgatctgtttgctgctgcaaac<br>cccggcattgaatgtgacggagagatgcacggcgacgccgcgttagatgccaatgttcgctcacactcgcttttggag<br>tcctccttgaccggagcagccaacgtacttatttgtccaaatttggacgcggctaacatcttgtttaatgtgcttaaaacta<br>cggggggcacggcactacaatcggaccgattctgatgggatgctcggcatccgcccatgtcttgactccatcatca<br>acagtgcgtcgcgtcgtgaatatgaccgccctggcggcggcccaagctattgctcttcgttcctga
Malic enzyme<br>(PD5092)<br>Fragment 1/2	tccgacaccaagcccattacctcgattgataaacgtgcgttgctgcgtcaggcggcactggaatatcacgagttcccc<br>aagcccggcaaggtggccatcgcggcaaccaagcccatggccaatcagcatgatctggccctggcttactcgcccg<br>gcgtggctgcgccctgcgaggaaatcgtcaaggacccggctgcggccttcaaatacaccagccgcggcaacctgg<br>tgggcgtgatctccaacggcacggcggtgctgggcctgggcgacatcggcgcgctggcctccaagcccgtgatgg<br>agggcaagggcgtgctgttcaagaagttcgccggcgtcgatgtgttcgatatcgaaatcgacgagaaggacccgca<br>aaagctggtcgacgtgattgctgcgctggagcccaccttcggcgccatcaatctcgaagacatcaaggcgcccgagt<br>gcttcttcgtggagcgcgagctgcgcaagcgcatgaacattcccgtcttccacgacgaccagcacggcacggccat<br>caccgtggctgcggccatggtcaatgcgctcaaggtcgccggcaagaagatcgaagaggtcaagctggtggcgtc<br>gggtgccggcgccgcggcgctggcctgcctgaacctgctgctggaagtgggtctgaagcgcgagaacgtgttcgt<br>gaccgatatcgccggcgtggtctacgagggccgcaccgagctgatggacccggacaaggcgctgtttgcccagaa<br>gaccgagctgcgcacgctgggcgaggtgatcgagggcgctgacgccttcctgggcctgtctgccggcaatgtgctc<br>aagcaggacatggtcaggaagatggcggccaggcccatcatcttcgcgctggccaaccccaacccggaaatcattc<br>ccgaagatgtgaaggcggtgcgcgacgacgccatcatcgccacgggccgcacggactaccccaaccaggtcaac<br>aacgtcctgtgcttcccctatatcttccgtggtgcgctggactgcggcgcgaccaccatcaccacctcgatggagatc<br>gcctgcgtgcacgcgattgccgcgctggcccaggccgagcagtccgaggaagtggcagccgcctatgtgggcga<br>ggtgctgagcttcggc
Malic enzyme<br>(PD5092)<br>Fragment 2/2	cccgagtacctgattcccaagcccttcgatccgcgcctgatgctggttgtggcgcctgccgtggcccaggctgcggc<br>cgaagccggcgtggccaagcgccccatccaggacatggatgcctaccgcgagcatctgaagaccttcgtctacgcc<br>tccggcaccatgatgaagcccatcgtgcatgccgccaagaaggcctccaagaagcgcgtggcctatgccgagggc<br>gaggaagagcgcatcctgcgcgcggcccagatcgtggtggacgagcgcattgcgcgcccgacgctgatcggccg<br>ccccgccatcattgcccagcgcatcgagaagttcggcctgcgcatgaaggaaggcgccgactacgacgtggtcaac<br>gtcgagcatgacgagcgctaccgcgacttctggcagagctatcaccgcatgacggagcgcaagggcatcaccgcg<br>cccatcgccaagatcgaaatgcgccgccgcctgacgctgatcggctccatgctgctgcacaagggcgaggtcgac<br>ggcctgatctgcggcacctggaacaacaccaatgtgcacctgaactacatcgatcaggtcatcggcaagcgcagcg<br>gcgtgaccacctatgcctgcatgaacggtctgctgctgcccgagcgccaggtattcctggtcgacacccacgtcaact<br>acgaccccacggccgagcagttggccgagatcaccatcatggccgccgaggaaatgatgcgcttcggtatcaagcc<br>caaggctgcgctgctgagccactccaacttcggctccagcaaccagcctagcgccgtcaagatgcgccagaccctg<br>gagctgctgcgcgagcaggcaccctggctggaagtggatggcgaaatgcacggcgatgtggctctggacggcaa<br>ggcccgcgccaagctcatgcccaacagcacgctgctgggtgatgccaacctgctggtctcgcccaatatcgacgcc<br>gccaacatcagctacaacttgctcaagcaggcggccggcggcggcattgccgtgggcccggtgctgctgggtgcg<br>gccaagcccgtgcatgtgctgacgcccagcaccacggtgcgccgtatcgtgaacatgacggcgttgaccgtggccg<br>acgccaacgtcagccgttaa
Isocitrate<br>dehydrogenase<br>(PD1820)<br>Fragment 1/2	agtacgcagcaacccaccatcatctacaccttgactgacgaggctccccgcctggctaccagcgcattcctgcccgt<br>gatccgtgctttcgcagcaccggccggcatcaatgtggagaccagcgatatctcgctggcaggccgtatcttgggcg<br>agttccccgactttttgaccgaagcccaacgcgctccgaacaacctggctgaactgggcaagctgacgctgacgcct<br>gaagccaacatcatcaagctgcccaatatctccgcatccgtgggccagctgcaagaagcgatcaaggaattgcagg<br>gcaagggctacgccatccccgactatcctgaaaccgccacgaccgatgccgaaaaggacatcaaggcacgctaca<br>acaggtgcatcggctctgccgtgaaccccgtgctgcgcgaaggcaactcggatcgccgcgcgcctgctgcggtcaa<br>gaactacgccaagaagcacccccattccatgggtgagtggaagcagtggtcgcaaacccatgtgtcgcacatggaa<br>gagggcgacttctaccacggcgaaaagtccatgacgctggacaaggcccgcgaagtgaagatggtgctggagacc<br>caatccggcaacagcatcgttttgaaggacaaggtcaagctgcaggctgcagaagtgatcgattccatgttcatgagc<br>aagaaggccttgtgcgctttctatgaaaaggaaatcgaagactgccgcgagtcgggcatcttgttctcgctgcacgtg<br>aaggccaccatgatgaaggtgtcccaccccatcgtgttcggtcactgcgtgaagattttctacaaggaagccttcgaaa

	agcacggcaagctgtttgatcagctgggcgtgaacgtgaacaatggcatggccaatctgtacgagaagattgagacc<br>ctgcctgcttcgcagcgcgaagaaatcatccgtgacctgcacaagtgccaggagcaccgtccacgtctggccatggt<br>cgattccgccaagggcatcaccaacttccactcgcccaa
Isocitrate dehydrogenase (PD1820) Fragment 2/2	cgacgtgatcgtggatgcttccatgcccgccatgatccgcgtgggcggcaagatgtgggccgctgacggcaagcct<br>tacgactgcaaggccgtgatgcctgagtccacctttgcccgtatctaccaggaagtgatcaacttctgcaagtggcac<br>ggcaacttcgaccccaagaccatgggcaccgtgcccaacgtgggtctgatggcgcaaaaggccgaagagtacggc<br>tcgcacgacaagacgtttgagatcgccgaagacggcgtggccaacatcgtggacatcgccaccggcgaagtgctg<br>ctgagccagaacgtggaagctggcgacatctggcgcatgtgccaggtcaaggatgctcctatccgcgactgggtga<br>agctggctgtgacccgtgcccgcaactccggcatgcctgccgtgttctggctggacccctatcgtccccacgaagcc<br>gagctgatcaagaaggtgcagacctacttgaaggaatacgacaccaacggcctggaaatccacatcctctcgcaagt<br>gcgcgccatgcgctacacgctggagcgcgtggcccgcggtctggacaccatttcggtgaccggcaacattctgcgt<br>gactacctgaccgacctgttcccgattctggaactgggcacctcggccaagatgctgtccatcgtgcctttgatggccg<br>gtggcggcatgtacgaaaccggtgcaggcggctccgctcccaagcatgtgcagcagctggtggaagaaaaccacc<br>tgcgctgggactcgctgggtgaattcctggccctggccgtctcgtttgaagacctgggcatcaaggaaaacaatgccc<br>gcgccaagctgctggccaagactctggatgaagccaccggcaagctgttggacgaggacaagtcgccttcgcgtcg<br>caccggcgagctggacaaccgtggttcgcagttctacatcgctctgtactgggcccaggctctggctgctcagagcg<br>aagatgcggaactggcggccaagttcgccactctggctaaaactctggccgagaacgaagccaagatcgtgggcg<br>agatgaaggaagtgcaaggcaaggctgccgatatcggcggctactacatgcccgacgtggccaagctggacgccg<br>tgatgcgccccagcgccaccttcaacgccgccatcgcgtcggtgtaa
Isocitrate dehydrogenase (PD1824) Fragment 1/1	agcacgtcccagcacatccaggttccgccgcagggggagaagatcagcgtcaacgcggacaagacactgaatgtt<br>ccgaatcaacccatcattccgttcatcgagggcgatggtgtcggtgttgatgtgacgccggtgatgcgcaaggtggct<br>gacgctgccgttgccaaggtatatggcgggcagcgcaagattgcctggatggaggtgtatgcgggtgagaaggcca<br>cgcgcatctacggccccgatgtctggctgcccgaggaaaccgtggctgccgtgcgcgactatgccgtgtccatcaa<br>gggcccgctgaccacgcccgtgggcggcggcatccgctcgctgaacgtggcgctgcgccaggagctggacctgt<br>atgtctgcctgcgccccgtgcagtacttcaagggcgtgccttcgccgctcaaggagcccgagaagacaaatatggtg<br>atcttccgcgagaactcggaggacatctacgcgggcatcgaatacgcggccggcagcgagaaggcgaaaaagctc<br>atcgacttcctggccaaggaattgggcgccaacaagatccgctttcccgaaacctcgggcatcgggatcaagcccgt<br>ctcgcgcgagggtacggagcgcctggtgcgcaaggccatccagtacgccatcgacaacaacaagcccagcgtga<br>ccctggttcacaagggcaacatcatgaagtacacggaaggcgccttccgtgactggggctacgcgctggcgcagcg<br>cgagttcggtgccgagctgatcgacggcggcccatggtgccgcatcaagcacccgaagacgggcaaggacatcat<br>catcaaggactcgatcaccgacgccttcttgcagcagattctgatgcgcccggccgaatattcggtggtggccacgct<br>caacctcaacggcgactacatctcggatgcgctggccgcgcaggtgggcgggatcggcattgctcccggtgccaat<br>ctgtcggagagcattgcctgcttcgaagccacccacggcacggcgccgcgctatgcgggcaaggactatgtgaacc<br>ccggctccatgattttgtgcgccgagatgatgctgcgccacatgggctggcgcgaggcagcggatctgatcgtcgag<br>tcgctggaaaaagccatccagagcaagcatgtgacctacgactttgcccgcctgatggacggagcgacccaggtttc<br>atgctcgggcttcggggatgtggtgatctcgatgatggactga
4-HB 3-monooxygenase (PD2627) Fragment 1/1	cgtacacaagtcgccatcatcggtgccggtccctcgggtttgctgctggggcagttgctctacaaggcgggcatcga<br>caacatcatcatcgagcagcgcagcgccgactatgtgctgggccgcatccgcgccggcgtgctggagcaggtgac<br>ggtggatctgctcaagcaggcgggtgccgacaagcgcatgaatgaggaaggtctgcctcacgatggcatcgagctg<br>ctgttcaagggcaagcgtcatcgcatcgacctcaacggcctgaccggcggcaagcgcgtgatggtctacggccaga<br>ccgaagtcacccgtgacctgatggaagtgcgtgcccaggaggggttgaccacggtgtacgaggccagtcacgtgc<br>agcccgtggacttcgagagcgacaagcccaaggtgcgttacgaaaaagacggccaggtgcacgagatcgaatgc<br>gacttcatcgccggttgcgacggctttcacggcatctgccgcgccagcgcgcctcaggacaagatcaagaccttcga<br>gaaggtctatcccttcggctggctgggcctgctctccgatacgccgcccgtgtcgcacgagctgatctacgccaacac<br>cgaacgcggctttgccctgtgcagccagcgcagcgccacgcgcagccgctactatctgcaggtgccgctgaccga<br>caaggtcgaggactggagcgatgaagcgttctgggaagagctcaagaagcgcctggaccccgaagcgcgcgcca<br>atctggtgaccggcccttcgctggagaaaagcattgcaccgctgcgcagctttgtgaccgagcccatgcgtttcggtc<br>gcatgttcctggccggcgacgccgcccacatcgtgcctcccacaggcgccaagggcctgaacctggcggcttccg<br>atgtaggctatctgtcgcaagcctttgtcgagtattaccaggaacagtccgaagccggcatcgatcgctactccgagc<br>agtgcctgcgtcgcgtctggaaggccgagcgtttctcctggtggatgacctccatgctgcacaacttccccggcgaag

	gcgagttcaacaccaaggtgcaggaagcagagctggactacatcgtccactccgaggccggctccacctcgctggc<br>cgagaactatgtgggtctgcctctggtgtaa
Phosphoenol-pyruvate synthase (PD3828) Fragment 1/2	tctcaactcttcgaagcgaccgcattagtcgttccgttcgaaaaactgcgcatgaccgacgtcgagtcggtcggcggc<br>aagaacgcctcgctcggcgagatgatttcgcaactgccccagggcgtgcgcgtgcctaccggctttgccaccacgg<br>cccatgccttccgcgagttcctggcctttgaaggtctggccggcaagatctctgccaagctggccgctctggacgtgg<br>acgatgtccgcgccctcgccgctgtcggcgcggaaattcgtgccatggtggaaagccagcctttccctgccgatctg<br>gaacaggccatccgcgaggaatttgtccgtctgcaaggcggcaatgccgaggcatcgtttgcggtgcgctcttcggc<br>cactgcagaagacctgcccgatgcctcgtttgccggccagcaggaaaccttcctgaacgtgttgggcatcgaagacg<br>tgctgcacaagatgaaggaagtgttcgcctcgctttacaacgaccgcgccatctcctaccgcgtgcacaagggcttcg<br>agcacgatgtggtggccctgtccgccggcgtgcagcgcatggtgcgttccgacaagggcgctgccggcgtgatgtt<br>caccatcgacaccgaatccggtttcgaggaagtggtcttcatcacctccagctacggcctgggcgagaccgtggtgc<br>agggcgcggtgaaccccgacgagttctatgtgcacaagcccatgctcaaggctggcaacaaggcactgatccgcc<br>gcaatctgggctccaagctgatccagatgatctttgccacgcccgaggaaaaggcggccgacggcaagctggtcaa<br>gaccaccgatgtggctcacgaactgcgcaaccgctattcgctgaccgacgaggaagtgcagcaactggcgcattac<br>gcgctggtgatcgagcagcattatggccgtcccatggatatcgaatggggcaaggacggcaccgacggccagctct<br>acatcctgcaggcgcgccctgaaaccgtgaagagccagtccaagggacaggccgagctgcgctacaagctcaag<br>ggcacgggcaccgtgctggccgaaggccgcgccatcggtcagaagatcggtaccggccccgtgcgcctggtgtc<br>cgacatctcgcagatggatcaggtgcaggccggcgacgtgctggtgaccgacatgaccgatcccaactgggagcc<br>cgtcatgaagaaggcttcggc
Phosphoenol-pyruvate synthase (PD3828) Fragment 2/2	catcgtcaccaaccgcggtggccgtacctgccacgccgccatcattgcacgcgagctgggtatccctgccgttgtgg<br>gctgcggcaatgcaaccgatctgctcaaggccgaaacactggttaccgtgtcctgtgcggaaggcgataccggcaa<br>gatctatgatggtctgctggaaaccgaagtgaccgaggtcaagcgtggcgagatgcccagcatccccaccaagatc<br>atgatgaacgtgggcaatccccagctggctttcgactttgcccagctgcccaacgaaggcgtgggtctggcccgcct<br>ggaattcatcatcaacaacaatatcggtgtccaccccaaggccatcctggactaccccgccgttgacgccgatctgaa<br>gaaggccgtcgagtccgtggcccgcggccatgcatctccccgcgcgttctacgtggacaaggtgaccgaaggcgt<br>ggcaacgattgccgccgctttctggcccaagcccgtgatcgtgcgcatgtccgacttcaagtccaacgaataccgca<br>agctcatcggcggcagccgttacgagcccgaggaagagaaccccatgctgggcttccgcggtgcagcgcgttacat<br>ctcggcagagttcggcgaagccttcaagatggaatgcgaagccctgctccgcgtgcgtgaggacatgggtctgacc<br>aacgtcaagatcatgatccccttcgtgcgtaccctgggccaggccaagcgcgtgactgagctgctggccgaaaacg<br>gcctcaagcgcggcgaaaatggcctgcagctgatcatgatgtgcgaagtgccttccaacgccgtgctggccgagga<br>gttcctcgaatacttcgacggcttctccgtgggctccaacgacctgacccagctgaccctgggcctggatcgcgactc<br>cggtctggagctgctggccgccgacttcgacgagcgcgaccccgccgtcaagaagctgctggctcgcgccatcaa<br>ggcctgccgcgatcagaacaagtatgtgggcatctgcggccaaggcccttcggaccaccccgacttcgccaagtgg<br>ctggccgatgagggcatctcctccatctccctgaaccctgacagcgtggtctccacctggcagaagctggctgaataa

Supplementary Table 23 | Nucleotide sequences of primers used for plasmid construction.

Name	Sequence (5'→3')	Description
pET28a-F	ATCTCTTCuGAGCACCACCACCACC	Amplification of pET-28a(+)-TEV for USER cloning, forward
pET28aNt-R	ATGGCCCuGAAAATAAAGATTCTCGCC GCT	Amplification of pET-28a(+)-TEV for USER cloning with Nt His-tag, reverse
pET28aCt-R	AGAGCCCAuGGTATATCTCCTTCTTAAA G	Amplification of pET-28a(+)-TEV for USER cloning with Ct His-tag, reverse
*Mdh*1281-F	ATGGGCTCuAGCAAGAAGCCCGTCCGT GT	Amplification of PD1281 for USER cloning into pET28a(+)-TEV in-phase with Ct His-tag, removes ATG, forward
*Mdh*1281-R	AGAAGAGAuCAGCAGGTGCTTGACGCC GTCTT	Amplification of PD1281 for USER cloning into pET28a(+)-TEV in-phase with Ct His-tag, removes Stop codon, reverse

*me*0934_1/2-F	AGGGCCAuACGCAAAATTTAAGCAGTGCG	Amplification of PD0934 fragment 1 for USER cloning into pET28a(+)-TEV in-phase with Nt His-tag, removes ATG, forward
*me*0934_1/2-R	AGCTTGuGCAACTGCTGGGGCGATA	Amplification of PD0934 fragment 1 for USER cloning into pET28a(+)-TEV in-phase with Nt His-tag, reverse
*me*0934_2/2-F	ACAAGCuGCGGCCGAATCAGGAGTA	Amplification of PD0934 fragment 2 for USER cloning into pET28a(+)-TEV in-phase with Nt His-tag, forward
*me*0934_2/2-R	AGAAGAGAuTCAGGAACGAAGAGCAATAGC	Amplification of PD0934 fragment 2 for USER cloning into pET28a(+)-TEV in-phase with Nt His-tag, reverse
*me*5092_1/2-F	AGGGCCAuTCCGACACCAAGCCCATTACC	Amplification of PD5092 fragment 1 for USER cloning into pET28a(+)-TEV in-phase with Nt His-tag, removes ATG, forward
*me*5092_1/2-R	AGGTACuCGGGGCCGAAGCTCAGCACCTC	Amplification of PD5092 fragment 1 for USER cloning into pET28a(+)-TEV in-phase with Nt His-tag, reverse
*me*5092_2/2-F	AGTACCuGATTCCCAAGCCCTTCGA	Amplification of PD5092 fragment 2 for USER cloning into pET28a(+)-TEV in-phase with Nt His-tag, forward
*me*5092_2/2-R	AGAAGAGAuTTAACGGCTGACGTTGGC	Amplification of PD5092 fragment 2 for USER cloning into pET28a(+)-TEV in-phase with Nt His-tag, reverse
*idh*1820_1/2-F	AGGGCCAuAGTACGCAGCAACCCACCAT	Amplification of PD1820 fragment 1 for USER cloning into pET28a(+)-TEV in-phase with Nt His-tag, removes ATG, forward
*idh*1820_1/2-R	ACGTCGTuGGGCGAGTGGAAGTTGGTGAT	Amplification of PD1820 fragment 1 for USER cloning into pET28a(+)-TEV in-phase with Nt His-tag, reverse
*idh*1820_2/2-F	AACGACGuGATCGTGGATGCTTCCATGC	Amplification of PD1820 fragment 2 for USER cloning into pET28a(+)-TEV in-phase with Nt His-tag, forward
*idh*1820_2/2-R	AGAAGAGAuTTACACCGACGCGATGGC	Amplification of PD1820 fragment 2 for USER cloning into pET28a(+)-TEV in-phase with Nt His-tag, reverse
*idh*1824-F	AGGGCCAuAGCACGTCCCAGCACATCCA	Amplification of PD1824 for USER cloning into pET28a(+)-TEV in-phase with Nt His-tag, removes ATG, forward
*idh*1824-R	AGAAGAGAuTCAGTCCATCATCGAGATCACCACA	Amplification of PD1824 for USER cloning into pET28a(+)-TEV in-phase with Nt His-tag, reverse
*4hb3mon*2627-F	AGGGCCAuCGTACACAAGTCGCCATCATC	Amplification of PD2627 for USER cloning into pET28a(+)-TEV in-phase with Nt His-tag, removes ATG, forward
*4hb3mon*2627-R	AGAAGAGAuTTACACCAGAGGCAGACCCA	Amplification of PD2627 for USER cloning into pET28a(+)-TEV in-phase with Nt His-tag, reverse
*peps3828*_1/2-F	AGGGCCAuTCTCAACTCTTCGAAGCGACC	Amplification of PD3828 fragment 1 for USER cloning into pET28a(+)-TEV in-phase with Nt His-tag, removes ATG, forward
*peps3828*_1/2-R	AAGCCTuCTTCATGACGGGCTCCCAGTT	Amplification of PD3828 fragment 1 for USER cloning into pET28a(+)-TEV in-phase with Nt His-tag, reverse

*peps3828*_2/2-F	AAGGCTuCGGCCATCGTCACCAACCGCGG	Amplification of PD3828 fragment 2 for USER cloning into pET28a(+)-TEV in-phase with Nt His-tag, forward
*peps3828*_2/2-R	AGAAGAGAuTTATTCAGCCAGCTTCTGCC	Amplification of PD3828 fragment 2 for USER cloning into pET28a(+)-TEV in-phase with Nt His-tag, reverse

Supplementary Table 24 | Primer sequences used for strain engineering.

Primer	Sequence
oALC831	TAGATGCCTGCAAGTCGAGCGGC
oALC832	TTCCTCCGGCCAATACAAGCGCATG
oALC833	AAGAGGAATACCGCGCCATGATG
oALC834	TTCGCCACACATCAATGACAATGGG
oALC837	GTGATGAAGGAACGCTTGCTGTCG
oALC839	GATCAGACCTGGAATTGTGAGCGG
oALC840	TTCGTTTTGACTAGATGGTTGCCC
oALC841	GAGAGCACCAGATCCACCTTGC
oALC874	ATCTGTGGGCGGTGAGATGTGG
oALC875	TGGAAGAAGTCGCCGTTGGG
oALC880	CGACAGGAATGTGCAGGGATCG
oALC882	CCTGTGGGTGACGTATTTCATGGG
oALC883	CGTCTATCTAAAAAGTTCAGTGCGCG
oALC884	TCACTACTACGACGACACCAAGCG
oALC885	AGACGATGGGGATGTTGACCG
oALC886	ATGAGCCACCAGTTGCAGGG
oALC887	ATACAGATCGTCGTAGCGGGCC
