# Supplementary material for: Complex regulation in a Comamonas platform for diverse aromatic carbon metabolism
Source: Nat Chem Biol. 2023 Feb 6;19(5):651–62. doi: 10.1038/s41589-022-01237-7 (PMC10154247; doi:10.1038/s41589-022-01237-7)
Supplement: Supplementary file 2 — Reporting Summary [file 41589_2022_1237_MOESM2_ESM.pdf]

nature research

Corresponding author(s): Ludmilla Aristilde

Last updated by author(s): Nov 14, 2022

Reporting Summary

Nature Research wishes to improve the reproducibility of the work that we publish. This form provides structure for consistency and transparency in reporting. For further information on Nature Research policies, see our Editorial Policies and the Editorial Policy Checklist.

Statistics

For all statistical analyses, confirm that the following items are present in the figure legend, table legend, main text, or Methods section.

n/a	Confirmed	
☐	☒	The exact sample size (*n*) for each experimental group/condition, given as a discrete number and unit of measurement
☐	☒	A statement on whether measurements were taken from distinct samples or whether the same sample was measured repeatedly
☐	☒	The statistical test(s) used AND whether they are one- or two-sided<br>*Only common tests should be described solely by name; describe more complex techniques in the Methods section.*
☒	☐	A description of all covariates tested
☒	☐	A description of any assumptions or corrections, such as tests of normality and adjustment for multiple comparisons
☐	☒	A full description of the statistical parameters including central tendency (e.g. means) or other basic estimates (e.g. regression coefficient) AND variation (e.g. standard deviation) or associated estimates of uncertainty (e.g. confidence intervals)
☐	☒	For null hypothesis testing, the test statistic (e.g. *F*, *t*, *r*) with confidence intervals, effect sizes, degrees of freedom and *P* value noted<br>*Give P values as exact values whenever suitable.*
☒	☐	For Bayesian analysis, information on the choice of priors and Markov chain Monte Carlo settings
☒	☐	For hierarchical and complex designs, identification of the appropriate level for tests and full reporting of outcomes
☐	☒	Estimates of effect sizes (e.g. Cohen's *d*, Pearson's *r*), indicating how they were calculated

Our web collection on statistics for biologists contains articles on many of the points above.

Software and code

Policy information about availability of computer code

Data collection	Details of all data collection are provided in the methods section. RNA sequencing was conducted at NUseq Core Facility at Northwestern University. All liquid chromatography mass spectrometry for metabolomics and proteomics were conducted on in-house instruments at Northwestern University and Chicago University as described in the method section. Protein assays were conducted at the Novo Nordisk Foundation Center for Biosustainability.
Data analysis	Details of data analysis and related software are provided in the methods section. All isotopologue measurements were extracted using MAVEN Version 2011.6.17 and corrected for the natural abundance of 13C using IsoCor v2. Quantification of intracellular metabolite pools and extracellular substrate depletion were conducted on ThermoScientific XcaliburTM 3.0 Quan Browser. OpenFlux2 was used for parallel labeling flux modeling. Protein-level relative abundances and standard errors were calculated in R 4.2.2. using the Arm postprocessing scripts for diDO-IPTL data (github.com/waldbauerlab). Kallisto version 0.46.0 was used to map reads to the Comamonas testosteroni KF-1 genome. Amuser tool 2 was used to design USER-primers. HPLC data was processed using the Chromeleon™ Chromatography Data System (CDS) Software 7.2.9 (Thermo Fisher Scientific).

For manuscripts utilizing custom algorithms or software that are central to the research but not yet described in published literature, software must be made available to editors and reviewers. We strongly encourage code deposition in a community repository (e.g. GitHub). See the Nature Research guidelines for submitting code & software for further information.

April 2020

Data

Policy information about availability of data

All manuscripts must include a data availability statement. This statement should provide the following information, where applicable:

- Accession codes, unique identifiers, or web links for publicly available datasets
- A list of figures that have associated raw data
- A description of any restrictions on data availability

The data supporting the findings in this study are available within the paper and supplementary information. Additionally, proteomic mass spectral data are available via ProteomeXchange under accession number PXD029813 and the MassIVE repository (massive.ucsd.edu) under accession number MSV000088418. Stable isotope assisted metabolomics LC-HRMS data are available in MetaboLights repository (www.ebi.ac.uk/metabolights) under the accession MTBLS3947. The RNA sequencing datasets generated during this study are available at the Gene Expression Omnibus with accession number GSE192852. NCBI Reference Sequence for C. testosteroni KF-1 was NZ_AAUJ02000001.1 (https://www.ncbi.nlm.nih.gov/nuccore/NZ_AAUJ02000001).

Field-specific reporting

Please select the one below that is the best fit for your research. If you are not sure, read the appropriate sections before making your selection.

☒ Life sciences ☐ Behavioural & social sciences ☐ Ecological, evolutionary & environmental sciences

For a reference copy of the document with all sections, see nature.com/documents/nr-reporting-summary-flat.pdf

Life sciences study design

All studies must disclose on these points even when the disclosure is negative.

Sample size	Sample sizes are noted in the figure legends and methods. No statistical methods were employed to determine the sample size. Triplicate is considered generally as an accepted standard for the minimum number of replicates for these experiments, which are labor, reagent, and cost intensive, to have conclusive evidence.
Data exclusions	The flux through malic enzyme was removed from the correlation analysis comparing flux to the substrate to product ratio. This was done to show the point that metabolite pools were correlated strongly to fluxes with the exception of malic enzyme, which indicated additional control on the flux through malic enzyme.
Replication	Each experiment had a minimum of 3 replicates. Isotope switch experiments were conducted with independent biological replicates on separate days and with separate initial biological stocks. All attempts at reproduction were successful.
Randomization	We are not conducting experiments on animals, humans, or from sources with innate variation. All experiments were conducted with bacteria derived from glycerol stocks of Comamonas testosteroni KF-1 purchased from ATCC.
Blinding	Blinding was not employed in this study because unintentional bias would not impact the collection or interpretation of the type of data collected in this manuscript.

Reporting for specific materials, systems and methods

We require information from authors about some types of materials, experimental systems and methods used in many studies. Here, indicate whether each material, system or method listed is relevant to your study. If you are not sure if a list item applies to your research, read the appropriate section before selecting a response.

Materials & experimental systems

n/a	Involved in the study	
☒	☐	Antibodies
☒	☐	Eukaryotic cell lines
☒	☐	Palaeontology and archaeology
☒	☐	Animals and other organisms
☒	☐	Human research participants
☒	☐	Clinical data
☒	☐	Dual use research of concern

Methods

n/a	Involved in the study	
☒	☐	ChIP-seq
☒	☐	Flow cytometry
☒	☐	MRI-based neuroimaging
